# Oral microbiome as a diagnostic biomarker for pancreatic cancer: a systematic review and meta-analysis of diagnostic accuracy

**DOI:** 10.1080/20002297.2025.2571188

**Published:** 2025-10-21

**Authors:** Maryam Koopaie, Mahnaz Fatahzadeh, Sajad Kolahdooz

**Affiliations:** a Department of Oral Medicine, School of Dentistry, Tehran University of Medical Sciences, Tehran, Iran; b Division of Oral Medicine, Department of Oral Medicine, Rutgers School of Dental Medicine, Newark, NJ, USA; c Universal Scientific Education and Research Network (USERN), Tehran University of Medical Sciences, Tehran, Iran

**Keywords:** Biomarker, chronic pancreatitis, diagnosis, meta-analysis, microbiome, mouth, pancreatic cancer, saliva, systematic review

## Abstract

**Background:**

This systematic review and meta-analysis aim to assess the diagnostic accuracy of the oral microbiome in detecting pancreatic cancer.

**Methods:**

A comprehensive search of relevant studies was conducted using key terms across multiple databases. The methodological quality of the included studies was assessed using the Quality Assessment Tool for Diagnostic Accuracy Studies-2 (QUADAS-2). Diagnostic accuracy metrics were calculated including specificity, sensitivity, likelihood ratios, and diagnostic odds ratio (DOR). Subgroup analyses were performed to explore the effects of oral sample collection methods, bacterial taxonomy, and oral microbiome classifications.

**Results:**

This systematic review included nine studies, comprising 188 study units with 6601 subjects The pooled specificity, sensitivity, and diagnostic odds ratio (DOR) for the use of a single oral microbiome were 0.70 (95% CI: 0.68−0.71), 0.65 (95% CI: 0.64−0.67), and 4.85 (95% CI: 4.11−5.74), respectively. Subgroup meta-analysis revealed that using multiple oral microbiome approaches could increase the DOR to 16.33. Subgroup analysis was performed based on bacterial phylum classification, multiple oral microbiomes, sampling methods, bacterial taxonomy, and subgenus-level taxonomy (*g_Streptococcus* and *g_Prevotella*).

**Conclusions:**

Oral microbiome holds promise as a diagnostic biomarker for pancreatic cancer, supporting its potential as a noninvasive tool for the screening and early detection of this malignancy.

## Introduction

Pancreatic cancer (PC), one of the most aggressive malignancies, has earned the reputation of being one of the deadliest cancers due to the difficulty in early detection [1,2]. Although PC ranks 12th in global incidence, it ranks as the seventh leading cause of cancer-related death worldwide, reflecting its poor prognosis and challenges in early detection [3]. The frequently vague and nonspecific symptoms in the initial stages render early diagnosis of PC a significant challenge [4–6]. Early diagnosis, however, is key to successful outcomes, and prognosis significantly depends on the disease stage at diagnosis [7]. The likelihood of survival is dramatically increased with early detection [6,8], and the survival rate of patients with early-stage disease is far superior to that of those with advanced-stage disease [8,9]. Diagnostic techniques for PC are available, but the invasive nature renders some of these approaches difficult [10].

Notwithstanding advancements in imaging modalities, their sensitivity for detecting early-stage PC remains limited [11,12]. While more advanced techniques offer higher diagnostic accuracy [13,14], they are invasive, require specialised expertise, and are highly operator-dependent [15,16]. Consequently, their use is impractical for widespread population-based screening. The limitations of these conventional and advanced diagnostic tools underscore the pressing need for innovative, noninvasive, and reliable biomarkers that can facilitate early PC detection [17], particularly in high-risk or asymptomatic individuals. Furthermore, chronic pancreatitis (CP), as a persistent inflammatory disease of the pancreas, is not only regarded as a significant risk factor for the development of PC but also presents a diagnostic challenge due to its overlapping clinical and radiological features with malignancy [18,19]. This overlap complicates the early and accurate differentiation between benign and malignant pancreatic conditions. Under these circumstances, there is a compelling need to identify novel, noninvasive biomarkers that can effectively distinguish PC from CP and healthy individuals, particularly at early stages of disease progression.

Recently, saliva has garnered increasing interest as a clinically relevant, noninvasive diagnostic biofluid [20]. The ease of collection, patient compliance, and potential for repeated sampling make saliva a practical and scalable medium for early disease detection and longitudinal health monitoring [21]. Saliva contains diverse biomolecules, including genomic DNA, mRNA, non-coding RNAs, proteins, metabolites, and microbial constituents from the oral cavity [22,23]. This complex composition renders saliva a valuable matrix for assessing systemic and local pathophysiological changes [24]. Concurrent advances in oral microbiome science have further highlighted the diagnostic potential of saliva by identifying the oral cavity as a dynamic microbial ecosystem with the capacity to reflect and influence systemic health [25]. The oral microbiome, comprised of bacteria, fungi, and viruses, is key in maintaining mucosal homeostasis [26]. However, disturbances in this community, referred to as dysbiosis, have been implicated in the aetiology of gastrointestinal disorders [27,28]. In recent years, the role of oral microbiota in cancer biology has gained significant attention, particularly in malignancies such as colorectal [29], gastric [30], and PC [31,32].

Histologically, the lobular architecture and parenchymal organisation of the parotid gland closely resemble the pancreas, and the morphology of parotid acinar cells is notably similar to that of pancreatic acinar cells [33,34]. Functionally, the parotid gland primarily produces salivary amylase and shares a high degree of exocrine resemblance to the pancreas, which secretes lipase and amylase, and the parotid gland primarily secretes salivary amylase [34,35]. A growing body of recent research supports a significant association between the oral microbiome and PC [36–40], including its potential utility in early detection [41–43]. Mechanistic and epidemiological evidence also increasingly point to a link between oral microbiota dysbiosis and PC development [36,40]. Specific pathogenic taxa, including *Porphyromonas gingivalis*, *Fusobacterium nucleatum*, and *Aggregatibacter actinomycetemcomitans*, have been associated with pancreatic tumorigenesis through several proposed mechanisms [44–47]. These include promoting chronic systemic inflammation, modulating immune responses, producing genotoxic metabolites, and translocating bacteria or their virulence factors to pancreatic tissue. Dysbiosis not only cultivates a pro-inflammatory microenvironment but may also directly induce DNA damage in pancreatic epithelial cells, thereby accelerating oncogenic transformation and tumour progression [48,49]. This convergence of microbiome science and oncology has led to the concept of the oncobiome, a cancer-associated microbial signature with promising potential for early detection [50,51]. The oral and gut microbiomes are increasingly recognised as functional players in carcinogenesis [52,53]. Similarly, emerging evidence suggests that the oral microbiome demonstrates one of the highest correlations with PC among various malignancies [54]. Therefore, distinct microbial signatures associated with PC may offer promising avenues for developing noninvasive biomarkers [55]. Such biomarkers could improve early detection, facilitate risk stratification, and inform microbiome-targeted therapeutic interventions. Given the often late-stage diagnosis and poor prognosis associated with PC, the clinical relevance of microbiome-based diagnostics is substantial [56]. Future research focused on the functional dynamics of the oral and gut oncobiome may uncover preventive, diagnostic, and therapeutic strategies for mitigating cancer risk and improving patient outcomes. Although the oral microbiome has gained considerable attention as a promising noninvasive biomarker for diagnosing PC [45,47,53,57], its reported diagnostic accuracy across individual studies remains inconsistent and fragmented.

This systematic review and diagnostic meta-analysis aims to evaluate the current evidence on the diagnostic performance of oral microbiome profiles for pancreatic carcinoma. By quantitatively assessing key diagnostic indicators and exploring methodological and taxonomic sources of heterogeneity, this study seeks to clarify the current landscape and guide future research toward developing practical noninvasive diagnostic tools for the early detection of pancreatic cancer. By offering a structured and integrative appraisal of the current literature, this review provides a necessary foundation for clarifying the diagnostic relevance of oral microbial signatures in the context of PC.

## Materials and methods

### Protocol and registration

The Preferred Reporting Items for a Systematic Review and Meta-analysis of Diagnostic Test Accuracy Studies (PRISMA-DTA) approach was used for the current systematic review and diagnostic meta-analysis [58] (Supplementary File 1), following the methodological guidance outlined in the Cochrane Handbook for Systematic Reviews of Diagnostic Test Accuracy [59]. The full details of this process have been meticulously documented and registered on the PROSPERO website (Registered ID: CRD42024589244).

### Study outline and search methodology

The PubMed search approach was structured using Medical Subject Headings (MeSH) terminology, with keywords derived from three fundamental concepts: ‘pancreatic neoplasms’, ‘microbiota’, ‘saliva’, and/or ‘oral cavity’. A thorough search was conducted using key terms across several research databases, including the Cochrane Library, Embase, LIVIVO, MEDLINE, Ovid, Web of Science, Scopus, and Google Scholar. All relevant databases were searched without limitations on language or date, with the search concluded in October 2023 and the latest update performed in January 2024. The search strategy employed for each database is provided in Supplementary File 2, Table S1. Studies retrieved through the search were imported into EndNote X 8.1 for reference management, with duplicates identified and excluded from the dataset.

### Eligibility criteria

Observational studies were eligible for inclusion if they evaluated the oral microbiome composition in patients with PC, confirmed by histopathological diagnosis. The index test was defined as the characterisation of the oral microbiome using validated microbiological or sequencing techniques. Studies were required to include a control group of healthy individuals or patients with chronic pancreatitis. The primary outcome was the diagnostic accuracy of oral microbiome profiles for differentiating PC from control groups, assessed through the availability of data sufficient to construct two-by-two contingency tables (true positive (TP), true negative (TN), false positive (FP), and false negative (FN)). Studies were excluded if they involved *in vitro* or *in vivo* experimental models, were review articles, meta-analyses, case reports, editorials, or letters, lacked an appropriate control group, or did not report extractable diagnostic accuracy data.

### Selection and screening of studies

A two-stage screening process was employed to identify eligible studies. Initially, the titles and abstracts of all retrieved studies were independently reviewed by two reviewers. Any disagreements were resolved through discussions with a third reviewer to reach a consensus. Full-text articles were then assessed individually by the authors, and only those deemed relevant were enrolled in the systematic review. Any remaining discrepancies were addressed through comprehensive discussions among the authors until a consensus was achieved.

### Data extraction

Relevant information gathered from each eligible study encompassed authors’ names, year of publication, country, PC stage, study phase, temperature of oral sample storage, oral sampling method, analytical and diagnostic methods (including sequencing techniques, platforms, taxonomic reference databases, and comparison models for PC diagnosis), Linnaean binomial classification of the identified oral microbial markers, sample size (TP, FP, TN, FN), as well as demographic characteristics of participants, including age and sex. In instances where the confusion matrix was not explicitly reported in the original manuscripts, essential diagnostic accuracy data were reconstructed through graphical data extraction using GetData Graph Digitiser (GetData Graph Digitiser 2.24) (Supplementary File 3). Subsequently, the extracted data were subjected to statistical and bioinformatics analysis to derive parameters necessary for constructing receiver operating characteristic (ROC) curves. Youden's index served as the method for calculating sensitivity (Sn) and specificity (Sp) [60]. All bacterial taxa were cross-referenced and verified using two established taxonomy databases: NCBI Taxonomy Browser [61] and the Genome Taxonomy Database (GTDB) [62]. This step ensured that pooled taxonomic assignments were harmonised and interoperable across datasets, particularly when aggregating data at genus and higher taxonomic levels.

### Research quality

The diagnostic accuracy studies in this systematic review and meta-analysis were assessed for methodological quality utilising the Quality Assessment Tool for Diagnostic Accuracy Studies-2 (QUADAS-2) [63]. To ensure robustness and inter-rater reliability, two reviewers independently assessed the quality of each study. Disagreements regarding the quality ratings were resolved through discussion, and a consensus was achieved. This process aimed to ensure consistency in the quality assessment and minimise any potential bias in evaluating study validity.

### Data analysis

Diagnostic test characteristics and diagnostic odds ratio (DOR) were calculated using the split component synthesis method, along with 95% confidence intervals (95% CI). Hierarchical summary receiver operating characteristic (HSROC) curves were further generated to visualise the trade-off between Sn and Sp. Additionally, the I-squared (I²) statistic was employed to quantify potential heterogeneity across studies [64]. STATA Version 17.0 (Stata Corp), R software package (Version 4.2.0), Meta-DiSc 1.4 (Madrid, Spain), MetaDTA: Diagnostic Test Accuracy Meta-Analysis v2.1.1 (https://crsu.shinyapps.io/MetaDTA/) [65], and GraphPad Prism 9.5.0 (GraphPad Software) were used for statistical and bioinformatics analyses. The positive likelihood ratio (PLR), negative likelihood ratio (NLR), and diagnostic odds ratio (DOR) were computed based on the confusion matrix. Subgroup analyses were conducted to compare the diagnostic performance across different oral sampling methods, bacterial taxonomic levels, oral microbiome classifications, and types of control groups (healthy individuals and patients with CP) in relation to patients with PC. A random effects meta-analysis was conducted to synthesise the findings and derive overall estimates of diagnostic test characteristics and the DOR estimate. Area under the curve (AUC) and summary receiver operating characteristic (SROC) curve were plotted. In this meta-analysis, we used the likelihood matrix of meta-analysis to synthesise the findings from individual studies and summarise the ability of a test to revise the prior probability of disease. Deeks' funnel plot asymmetry test was used to assess publication bias in the included studies.

## Results

### Study selection

**Figure 1.**
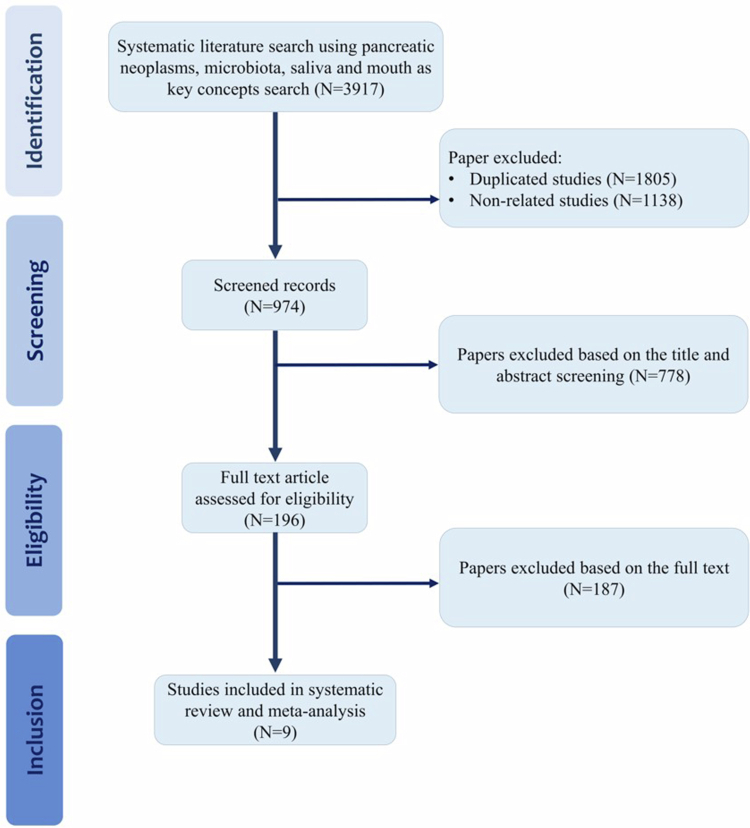
Schematic representation of the study selection process for the systematic review and meta-analysis.

A comprehensive flow diagram outlining the study selection process is provided in Figure 1. An initial exhaustive search of the database yielded 3917 studies. Duplicate entries (*n* = 1805) were detected and removed using EndNote X 8.1 software. After screening the titles and abstracts, 1138 studies were excluded due to their lack of relevance to the research question. Following this step, 778 studies from the remaining 974 were excluded after thoroughly reviewing their titles and abstracts. The full texts of the remaining 196 publications were subsequently reviewed in detail. This final selection stage led to the exclusion of an additional 187 studies. As shown in Figure 1, nine studies met the inclusion criteria and were ultimately included in the systematic review and meta-analysis [41,66–73].

### Study characteristics

Nine studies comparing the oral microbiome of PC patients to the control group published between 2012 and 2023 were included in our review (Table 1).

**Table 1.** Overview of data and characteristics derived from studies included in the systematic review and meta-analysis.

	Bibliographic details			Type and stages of diseases		Study phase, Oral sampling, and storage condition			Analytical and diagnostic methods				Sample size			Age and sex	
No.	Authors	Year	Country	Type of diseases*	Stages of PC**	Phase of study	Oral sampling method	Temperature of oral sample storage	Sequencing techniques***	Platform^✳^	Taxonomy reference^✳✳^	Comparison model for PC diagnosis	PC cases	CP cases	Healthy control	age and sex cases^✳✳✳^	age and sex control
1	Farrell et al. [66]	2012	USA	PC	resectable PC	discovery	unstimulated saliva	−80 °C	16S rRNA gene-based microarray	HOMIM	HOMIM	multiple oral microbiome	10	-	10	66.5 8 M, 2F	66.4 8 M, 2F
				PC and CP	resectable PC	validation	unstimulated saliva	−80 °C	qPCR	HOMIM	qPCR	multiple oral microbiome	28	27	28	69.9 17 M, 11F	65.1 18 M, 10F
2	Torres et al. [69]	2015	USA	PC	-	discovery	saliva	−80 °C	16S rRNA gene sequencing, QIIME v1.8.0	Illumina MiSeq	OTU clustering, qPCR	multiple oral microbiome	8	-	22	71.1 6 M, 2F	- 12 M, 10F
3	Lu et al. [70]	2019	China	PC (PHC)	30 stage I	discovery	tongue coating microbiome	−80 °C	16S rRNA gene sequencing	Illumina MiSeq	RDP classifier, Rarefaction analysis	single oral microbiome & multiple oral microbiome	30	-	25	50.80 21 M, 9F	48.16 20 M, 5F
4	Vogtmann et al. [71]	2020	Iran	PDAC	stage I, II, III, IV	discovery	saliva	−70 °C	16S rRNA gene QIIME 2	Illumina MiSeq	HOMIM v14.51	single oral microbiome	273	-	285	-	-
5	Wei et al. [72]	2022	China	PDAC	16 I-IIB, 18 III-IV 17 unresectable PC 17 resectable PC	discovery	saliva	−80 °C	Internal transcribed spacer (ITS1) region of ribosomal RNA	Illumina	FUNGuild database	single oral mycobiota	34	-	35	60.97 19 M, 15F	60.51 20 M, 15F
6	Kartal et al. [73]	2022	Spanish cohort	PDAC and CP	8 I, 22 II, 6 III, 7 IV	discovery	mouthwash	−80 °C	Shotgun metagenomic sequencing	Illumina HiSeq	mOTU v2.5	single oral microbiome	43	12	43	about 70	about 70
7	Nagata et al. [67]	2022	Spanish cohort	PDAC	8 I, 22 II, 6 III, 7 IV	validation	mouthwash	−80 °C	Shotgun metagenomic sequencing	Illumina HiSeq X	mOTUs2 v2.1.1	multiple oral microbiome	43	-	45	about 70	about 70
			Japanese cohort	PDAC	20 early stages (13 I, 7 II) and 27 late stages (17 resectable)	discovery	stimulated saliva	−80 °C	Shotgun metagenomic sequencing	Illumina HiSeq X	mOTUs2 v2.1.1	multiple oral microbiome	47	-	235	about 70 26 M, 21F	about 70 130 M, 105F
8	Chen et al. [68]	2023	China	PC and CP	30 I, II and 7 III, IV	discovery: 20 PC vs. 16 HC	unstimulated saliva	−80 °C	16S rRNA gene sequencing	Illumina MiSeq	RDP classifier via QIIME	multiple oral microbiome	37	15	36	57.19 19 M, 18F	56.41 18 M, 18F
						validation: 17 PC vs. 20 HC											
9	Zeng et al. [41]	2023	China (Japanese cohort)	PDAC	20 early stages and 27 late stages	discovery	stimulated saliva	−80 °C	Shotgun metagenomic sequencing	llumina HiSeq X	mOTUs2 v2.1.1 and VFDB**	multiple oral microbiome	47	-	235	about 70 26 M, 21F	about 70 130 M, 105F

*PC: pancreatic cancer, CP: chronic pancreatitis, PDAC: pancreatic ductal adenocarcinoma, PHC: pancreatic head carcinoma. **Staging is based on the TNM. ***16S rRNA: 16S ribosomal RNA, qPCR: Quantitative polymerase chain reaction or Quantitative real-time PCR, QIIME: Quantitative Insights Into Microbial Ecology. ^✳^HOMIM: Human oral microbe identification microarray. ^✳✳^OUT: Operational taxonomic unit, RDP classifier: Ribosomal database project classifier, mOTU = marker gene-based operational taxonomic unit, VFDB: Virulence factors of pathogenic bacteria database. ^✳✳✳^F: Female, M: Male

In this meta-analysis, each study unit is defined as an independent evaluation of a single biomarker comparing case and control groups. If a primary study independently assesses *N* biomarkers, it contributes *N* study units to the meta-analysis. Given *k* primary studies, where the *i-th* study evaluates *x*_*i*_ biomarkers separately, the total number of study units included is calculated as $\sum_{i=1}^{k}x_{i}$​. A total of one hundred eighty-eight study units were included in our meta-analysis. This comprised 6601 subjects (3594 PC patients and 3007 noncancerous controls, including 2780 healthy individuals and 227 CP patients) (Supplementary File 4). These study units either compared the oral microbiome between PC patients and various control groups, using single oral microbiome comparisons (133 study units compared PCs to healthy controls, 48 study units compared PCs to CPs) and multiple oral microbiome comparisons (7 study units compared PCs vs. healthy controls). In this review, 98 unique oral microbes and two fungi were examined based on species classification. Among the nine included studies, eight conducted an examination of oral bacteria [41,66–71,73], and one involved oral fungal organisms [74] (Supplementary File 4). In addition to including healthy individuals as controls, three of the nine studies in this review used patients with CP as control subjects [66,68,73] (Table 1). Among the nine studies included in the systematic review, two used unstimulated saliva [66,68], three examined saliva [69,71,72], two used mouthwash [41,73], one examined tongue coating microbiome [70], and one study assessed both mouthwash (Spanish cohort) and stimulated saliva (Japanese cohort) [67]. In five studies, a combination of more than one oral bacterium was used as a biomarker in the analyses [41,66–69], whereas in three studies, only one type of oral bacterium was used [71–73], and in one study, both single and multiple types oral bacteria were used as markers for PC diagnosis [70] (Table 1). All oral samples were stored at −80 °C [41,66–70,72,73], except for one study where the oral samples were stored at −70 °C [71]. Various sequencing methods were employed for the analysis of the oral microbiome, including 16S rRNA gene-based microarray (one study) [66], qPCR (two studies) [66,69]], and 16S rRNA gene sequencing (four studies) [68–71] using platforms such as Illumina MiSeq and Illumina HiSeq X. Additionally, shotgun metagenomic sequencing (three studies) [41,67,73] was performed using Illumina HiSeq and Illumina HiSeq X platforms. These studies utilised a variety of databases and methods for taxonomic classification, including HOMIM, RDP classifier, OTU clustering, rarefaction analysis, FUNGuild database, and mOTU.

### Quality assessment

**Figure 2.**
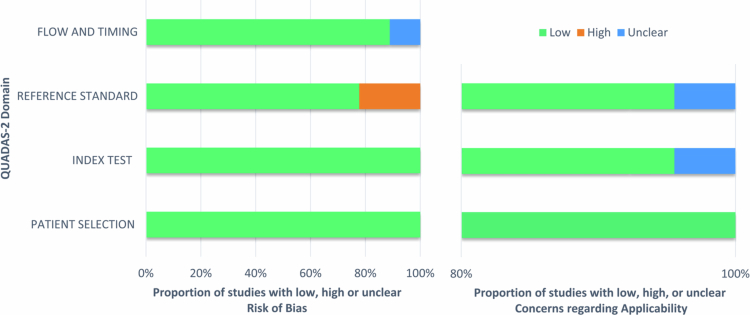
Assessment of the overall methodological quality of the included studies using QUADAS−2.

Studies identified as having a high risk of bias presented concerns regarding the reference standard. Issues with the reference standard were also reported in research assessed as having an unclear risk of applicability. The research categorised as having a low risk of bias reflected both appropriate patient selection and a valid index test. In contrast, studies categorised as having a low risk of applicability reflected patient selection (Figure 2). The flow and timing of participants were identified in research categorised as having an unclear risk of bias (Supplementary File 5).

### Diagnostic odds ratio (DOR) of a single oral microbiome

**Figure 3.**
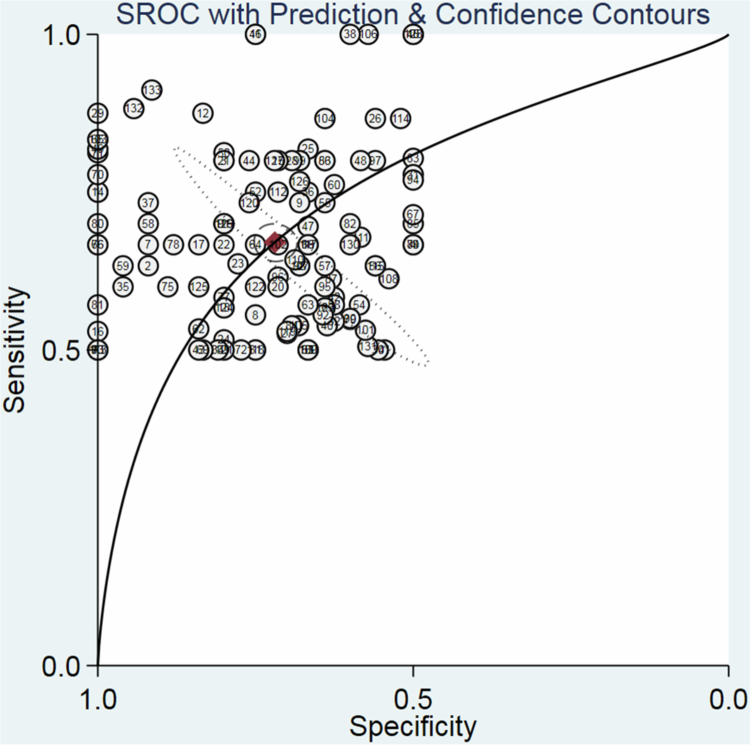
SROC curve of the use of a single oral microbiome in the diagnosis of PC.

The overall specificity and sensitivity for diagnosing pancreatic cancer (PC) versus controls using a single oral microbiome were 0.70 (95% CI: 0.68–0.71) and 0.65 (95% CI: 0.64–0.67), respectively (Figure 3). The combined PLR and NLR were 2.03 (95% CI: 1.87−2.20) and 0.50 (95% CI: 0.47−0.54), respectively. The pooled DOR was 4.85 (95% CI: 4.11−5.74) (Supplementary File 6 (Figures), Figure S1), with an AUC of 0.74 (95% CI: 0.73−0.75) (Figure S2). HSROC curves were used to evaluate the overall DOR associated with the oral microbiome for PC, taking into account the sample sizes of the study units with 95% CI (Figure S2). The likelihood matrix for diagnosing PC based on the single oral microbiome is shown in Figure S3. Figure S4 depicts an evaluation of publication bias using Deeks' funnel plot method in the meta-analysis of the single oral microbiome for PC diagnosis. The funnel plot shows the relationship between the DOR and the inverse of the square root of the effective sample size for each included study unit.

### Subgroup analysis

Subgroup analysis was conducted considering the phylum classification of bacteria (Supplementary File 1, Figure S5-S21), multiple oral microbiome (Figure S22-S24), Oral sampling method (Figure S25-S27), bacterial taxonomy (Figure S28-S41), subgenus-level taxonomy (*g_Streptococcus*)^1^^1^*k_Bacteria | p_Firmicutes (Bacillota) | c_Bacilli | o_Lactobacillales | f_Streptococcaceae | g_Streptococcus* (Figure S42, S43), and subgenus-level taxonomy (*g_Prevotella*)^2^^2^*k_Bacteria | p_Bacteroidetes | c_Bacteroidia | o_Bacteroidales | f_Prevotellaceae | g_Prevotella*
(Figure S44, S45). In addition, a meta-analysis was conducted on PC versus CP based on the single oral microbiome (Figure S46, S47), followed by a subgroup analysis based on common genus levels (*g_Streptococcus*^1^ and *g_Prevotella*^2^) (Figure S48-S51).

### Heterogeneity test

A Spearman's correlation coefficient of 0.12 (*p* = 0.166) was observed, suggesting a possible threshold effect on diagnostic accuracy. The single oral microbiome-based test has high diagnostic accuracy for pancreatic cancer (*α* = 1.553, *p* < 0.001), with no significant effect of threshold variation (*β* = −0.146, *p* = 0.137). Heterogeneity is moderate (Tau² = 0.223), and the model converged after 20 REML iterations (Supplementary File 2, Table S2). The I^2^ heterogeneity of Sp, Sn, PLR, NLR, and DOR were 33.7% (*p* < 0.0001), 37.0% (*p* < 0.0001), 15.3% (*p* < 0.076), 19.7% (*p* = 0.029), and 26.4% (*p* = 0.004), respectively.

#### Phylum classification of bacteria

The classification of the oral microbiome based on bacterial phyla for subgroup analysis of PC diagnosis is shown in Figure S5. The classification of a single oral microbiome was performed in nine groups, including:

A: *k_Bacteria | p_Actinobacteria*,

B: *k_Bacteria | p_ Firmicutes (Bacillota)*,

C: *k_Bacteria | p_Bacteroidetes*,

D: *k_Bacteria | p_Fusobacteria*,

E: *k_Bacteria | p_Proteobacteria*,

F: *k_Bacteria | p_Pseudomonadota*,

G: *k_Bacteria | p_Saccharibacteria*,

H: *k_Bacteria | p_Spirochaetes*,

I: *k_Fungi | p_Ascomycota*.

A forest plot of the subgroup analysis of the oral microbiome in PC diagnosis using phylum classification is shown in Figure S6. The pooled DOR values for each subgroup were arranged in ascending order (from lowest to highest). The SROC curve of the oral microbiome for PC diagnosis based on phylum level is shown in Figure S7. The pooled DOR of the bacteria in group A (7.30, 95% CI: 4.83−11.02) surpassed that of group E (5.15, 95% CI: 3.42−7.74), that of group E outperformed that of group D (4.97, 95% CI: 1.99−12.37), that of group D was superior to that of group B (4.76, 95% CI: 3.81−5.93), and that of group B was greater than that of group C (3.34, 95% CI: 2.54−4.39) (Supplementary File 2, Table S3).

#### k_Bacteria | p_Actinobacteria

Forest plot of the subgroup analysis of the oral microbiome in the diagnosis of PC using the phylum bacteria (*k_Bacteria | p_Actinobacteria*) are shown in Figure S8. The studies were divided into three subgroups based on bacterial classes: A) *c_Actinobacteria*, B) *c_Coriobacteriia*, and C) *c_Actinomycetia*. The overall Sn, Sp, DOR, and AUC of *c_Actinobacteria* for PC diagnosis were 0.66 (95% CI: 0.59−0.73), 0.80 (95% CI: 0.73–0.86), 8.08 (95% CI: 4.30–15.16), and 0.76 (95% CI: 0.71−0.81), respectively (Figure S8 and Table S3). Results indicated that the *c_Actinobacteria* group exhibited the highest DOR, followed by *c_Actinomycetia* (7.43, 95% CI: 3.29−16.79) and *c_Coriobacteriia* (3.73, 95% CI: 0.44−31.51). SROC curves were plotted in various colours for each bacterial class (Figure S9). The evaluation of publication bias using Deeks' funnel plot in a subgroup analysis of the oral microbiome (*k_Bacteria | p_Actinobacteria*) depicts no significant impact on the research findings (Figure S10).

#### k_Bacteria | p_Firmicutes (Bacillota)

Forest plot of the subgroup analysis of the oral microbiome according to the bacterial phylum (*k_Bacteria | p_Firmicutes (Bacillota)*) is shown in Figure S11. Meta-analysis of the phylum-level bacterial classification (*k_Bacteria | p_Firmicutes* (*Bacillota*)) revealed a pooled Sn of 0.66 (95% CI: 0.63–0.69), Sp of 0.73 (95% CI: 0.70–0.76), DOR of 4.76 (95% CI: 3.81–5.93), and an AUC of 0.73 (95% CI: 0.71−0.75) for PC diagnosis (Table S3). The subgroup analysis was divided into five subgroups based on bacterial class: A: *c_Bacilli*, B: *c_Clostridia*, C: *c_Erysipelotrichia*, D: *c_Negativicutes*, and E: *c_Tissierellia*. Subgroup analysis revealed that *c_Clostridia* presented the highest DOR (7.49, 95% CI: 5.20−10.80), followed by *c_Negativicutes* (3.83, 95% CI: 1.85−7.93), and *c_Bacilli* (3.42, 95% CI: 2.46−4.77) (Figure S12). As shown in Figure S13, Deeks' funnel plot analysis indicated that publication bias had no significant effect on the subgroup analysis results

#### k_Bacteria | p_Bacteroidetes

The forest plot of the subgroup analysis of the oral microbiome, based on the bacterial phylum (*k_Bacteria | p_Bacteroidetes*), is shown in Figure S14. Meta-analysis of the phylum-level bacterial classification (*k_Bacteria | p_Bacteroidetes*) revealed an overall Sn of 0.66 (95% CI: 0.62–0.70), Sp of 0.63 (95% CI: 0.58–0.68), DOR of 3.34 (95% CI: 2.54–4.39), and an AUC of 0.69 (95% CI: 0.67−0.71) for PC diagnosis (Table S3). Subgroup analysis on the class of bacteria (*k_Bacteria | p_Bacteroidetes | c_Bacteroidia*) was performed as follows: A: *o_Bacteroidales*, B: *o_Bacteroidales | f_Prevotellaceae*, C: *o_Bacteroidales | f_Porphyromonadaceae*, D: *o_Bacteroidales | f_Rikenellaceae*, E: *o_Bacteroidales | f_Bacteroidaceae*, F: *o_Bacteroidales | f_Tannerellaceae* (Table S3). The subgroup meta-analysis results indicated that D had the highest DOR for PC (5.57, 95% CI: 0.62−49.96), followed by C (5.02, 95% CI: 2.55−9.87), and E (2.95, 95% CI: 1.26−6.93) (Figure S14 and Table S3). SROC curve, using various colours for each bacterial subgroup, is presented in Figure S15. Deeks' funnel plot asymmetry test was employed to assess publication bias, as shown in Figure S16.

#### k_Bacteria | p_Fusobacteria

The SROC curve and forest plot of the subgroup analysis of the oral microbiome according to the bacterial phylum (*k_Bacteria | p_Fusobacteria*) are shown in Figures S17 and S18, respectively. The meta-analysis revealed a pooled Sn of 0.73 (95% CI: 0.63–0.82), Sp of 0.63 (95% CI: 0.51–0.74), DOR of 4.97 (95% CI: 1.99–12.37), and AUC of 0.75 (95% CI: 0.74−0.76) for PC diagnosis (Table S3).

#### k_Bacteria | p_Proteobacteria

**Figure 4.**
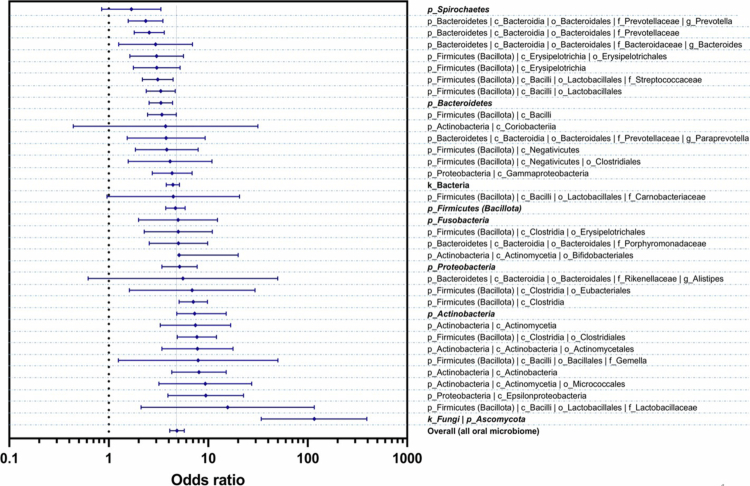
Summary of DOR results of the subgroup analysis (ascending order) based on the oral microbiome taxonomy for PC diagnosis.

A forest plot of the subgroup analysis of the oral microbiome according to the bacterial phylum (*k_Bacteria | p_Proteobacteria*) is shown in Figure S19. Pooled Sn of 0.64 (95% CI: 0.57–0.70), Sp of 0.74 (95% CI: 0.68–0.80), DOR of 5.15 (95% CI: 3.42–7.74), and an AUC of 0.76 (95% CI: 0.73−0.79) for PC diagnosis were obtained (Table S3). SROC curves were plotted using two colours for the bacterial subgroup, and the circle's diameter is presented as the study sample size (Figure S20). Deeks' funnel plot asymmetry test for evaluating publication bias is demonstrated in Figure S21. A summary of the DOR results of the subgroup analysis of PC diagnosis (ascending order) using oral microbiome taxonomy order is shown in Figure 4.

### Multiple oral microbiome

**Figure 5.**
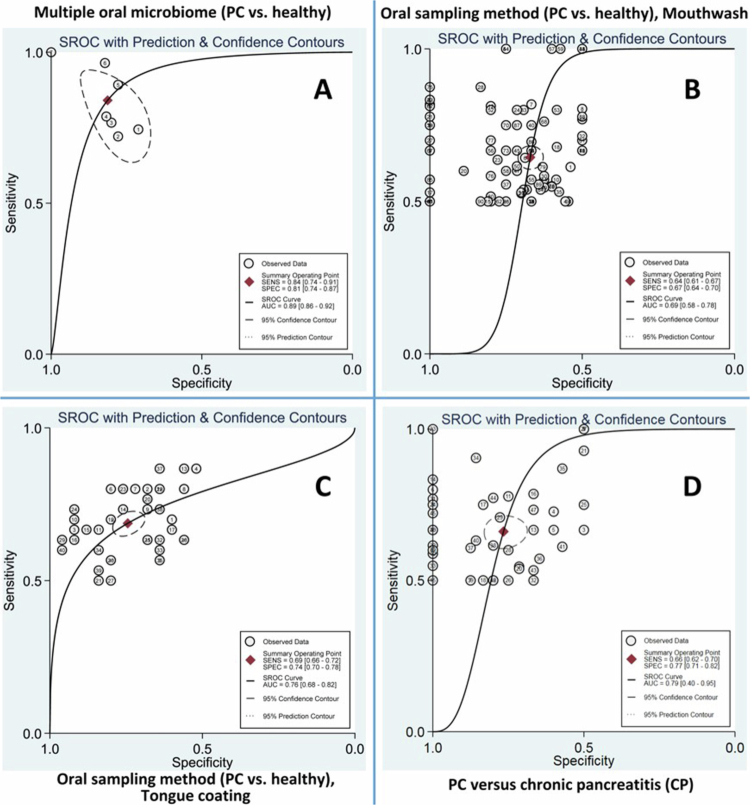
SROC curve of subgroup meta-analysis for PC diagnosis; A: multiple oral microbiomes (PC vs. healthy), B: mouthwash use (PC vs. healthy), C: tongue coating use (PC vs. healthy), and D: oral microbiome use (PC vs. chronic pancreatitis (CP)).

Figure S22 displays a forest plot of subgroup analyses investigating the DOR of multiple oral microbiome across different countries for PC diagnosis. Meta-analysis of using multiple oral microbiome revealed a pooled Sn of 0.81 (95% CI: 0.75–0.86), Sp of 0.78 (95% CI: 0.74–0.81), DOR of 16.32 (95% CI: 8.37–31.86), and an AUC of 0.86 (95% CI: 0.84−0.88) (Table S4). SROC curve (Figure 5A) and Deeks' funnel plot asymmetry test (Figure S24) were plotted.

### Oral sampling method

Subgroup analysis of the oral microbiome based on the oral sampling method (mouthwash, tongue coating microbiome, and saliva) was performed (Figure S25). The pooled DOR of using mouthwash, the tongue coating microbiome, and saliva for PC diagnosis were 3.45 (95% CI: 2.81−4.23), 6.19 (95% CI: 4.97–7.72), and 24.62 (95% CI: 0.67–908.06), respectively (Table S4). The pooled Sn, Sp, and AUC of mouthwash for PC diagnosis were 0.64 (95% CI: 0.61–0.67), 0.67 (95% CI: 0.64–0.70), and 0.70 (95% CI: 0.69–0.71), respectively (Figure 5B and Table S4). The overall Sn, Sp, and AUC of the tongue coating microbiome for PC diagnosis were 0.68 (95% CI: 0.66–0.71), 0.73 (95% CI: 0.70–0.76), and 0.77 (95% CI: 0.76–0.78), respectively (Figure 5C and Table S4).

### Bacterial taxonomy

Subgroup analysis of the oral microbiome based on the bacterial taxonomy (Figure S28) at six levels was performed. A summary of the DORs of the subgroup analysis for PC diagnosis using bacterial taxonomy is shown in Figure S29.

#### Phylum level

Subgroup analysis of the oral microbiome was conducted at the phylum level (Figure S30). The combined Sn, Sp, DOR, and AUC at the phylum level for PC diagnosis were 0.77 (95% CI: 0.68–0.84), 0.71 (95% CI: 0.61–0.80), 8.86 (95% CI: 4.71–16.64), and 0.82 (95% CI: 0.79–0.85), respectively (Figure S31 and Table S4).

#### Class level

Figure S32 presents a subgroup analysis of the oral microbiome, stratified by class. The pooled Sn, Sp, DOR, and AUC at the class level were 0.68 (95% CI: 0.61–0.74), 0.79 (95% CI: 0.72–0.85), 8.26 (95% CI: 4.72–14.46), and 0.79 (95% CI: 0.75–0.83), respectively (Figure S33 and Table S4).

#### Order level

Subgroup analyses of the oral microbiome at the order level were performed (Figure S34). The overall Sn, Sp, DOR, and AUC of the order level of the oral microbiome for PC diagnosis were 0.71 (95% CI: 0.65–0.76), 0.74 (95% CI: 0.68–0.80), 7.31 (95% CI: 4.44–12.05), and 0.79 (95% CI: 0.76–0.82), respectively (Figure S35 and Table S4).

#### Family level

Figure S36 shows the results of the subgroup analysis of the oral microbiome at the family level. The combined Sn, Sp, DOR, and AUC of the order level for PC diagnosis were 0.67 (95% CI: 0.62–0.72), 0.73 (95% CI: 0.68–0.78), 5.69 (95% CI: 3.88–8.34), and 0.75 (95% CI: 0.72–0.78), respectively (Figure S37 and Table S4).

#### Genus level

The overall Sn, Sp, DOR, and AUC at the genus level for PC diagnosis were 0.64 (95% CI: 0.61−0.68), 0.68 (95% CI: 0.65−0.71), 6.68 (95% CI: 4.11−10.87), and 0.77 (95% CI: 0.74−0.80), respectively (Figures S38, S39, and Table S4).

#### Species level

The pooled Sn, Sp, DOR, and AUC of the species level were 0.64 (95% CI: 0.61–0.67), 0.67 (95% CI: 0.64–0.70), 3.41 (95% CI: 2.77–4.19), and 0.69 (95% CI: 0.68–0.70), respectively (Figure S40, S41, and Table S4).

### Subgenus-level taxonomy

Subgroup analysis based on subgenus-level taxonomy (*k_Bacteria | p_Firmicutes (Bacillota) | c_Bacilli | o_Lactobacillales | f_Streptococcaceae | g_Streptococcus*) revealed overall DOR, Sn, Sp, and AUC values of 3.11 (95% CI: 2.18−4.43), 0.62 (95% CI: 0.58−0.67), 0.67 (95% CI: 0.61−0.73), and 0.68 (95% CI: 0.65−0.71), respectively (Figure S42, S43, and Table S4). Furthermore, subgroup analysis based on the subgenus-level taxonomy (*k_Bacteria | p_Bacteroidetes | c_Bacteroidia | o_Bacteroidales | f_Prevotellaceae | g_Prevotella*) revealed that the combined DOR, Sn, Sp, and AUC equal to 2.35 (95% CI: 1.57−3.50), 0.60 (95% CI: 0.53−0.66), 0.62 (95% CI: 0.54−0.69), and 0.64 (95% CI: 0.61−0.67), respectively (Figure S44, S45 and Table S4).

### Pancreatic cancer (PC) versus chronic pancreatitis (CP)

The combined DOR for distinguishing between PC and CP was 5.78 (95% CI: 4.06−8.23), with a Sn of 0.65 (95% CI: 0.62−0.69), Sp of 0.77 (95% CI: 0.71−0.82), and an AUC of 0.76 (95% CI: 0.74−0.78) (Figure 5D, S46, and Table S4).

### Subgenus-level taxonomy for differentiating PCs from CPs

Subgroup analysis based on subgenus-level taxonomy (*k_Bacteria | p_Firmicutes (Bacillota) | c_Bacilli | o_Lactobacillales | f_Streptococcaceae | g_Streptococcus*) revealed overall DOR, Sn, Sp, and AUC equal to 5.55 (95% CI: 3.23−9.54), 0.64 (95% CI: 0.59−0.70), 0.76 (95% CI: 0.66−0.84), and 0.76 (95% CI: 0.72−0.80), respectively (Figure S48, S49, and Table S4). Furthermore, subgroup analysis based on the subgenus-level taxonomy (*k_Bacteria | p_Bacteroidetes | c_Bacteroidia | o_Bacteroidales | f_Prevotellaceae | g_Prevotella*) revealed that the combined DOR, Sn, Sp, and AUC equal to 6.77 (95% CI: 2.47−18.58), 0.65 (95% CI: 0.55−0.74), 0.79 (95% CI: 0.60−0.92), and 0.80 (95% CI: 0.74−0.86), respectively (Figure S50, S51, and Table S4).

## Discussion

Our systematic review of diagnostic test accuracy included nine eligible studies, comprising 188 study units, 3594 patients with PC, and 3007 non-cancerous controls (including 2780 healthy individuals and 227 CP patients). This systematic review analysed 98 unique species of the oral microbiome, as well as two fungal species, at the species level. According to the Mandrekar criterion [75], findings from our diagnostic meta-analysis (DOR = 4.85 and AUC = 0.74) suggest that the oral microbiome demonstrates ‘acceptable’ diagnostic accuracy. Subgroup meta-analysis further revealed that the highest DOR at the phylum level was observed for *k_Fungi* (DOR = 116.43), followed by *k_Bacteria | p_Actinobacteria* (DOR = 4.41).

Unlike colorectal cancer, which benefits from population-based screening programs such as faecal immunochemical test (FIT) and colonoscopy [76–78], pancreatic cancer lacks effective screening strategies. Emerging liquid biopsy methods, including ctDNA, exosomes, and salivary RNAs, could complement oral microbiome biomarkers, and combining these approaches may improve early detection and triage of high-risk individuals. Yang et al. explored the dysbiosis of the fungal microbiota in gastric cancer, identifying a potential role for *Ascomycota fungi* in the carcinogenesis of gastrointestinal malignancies [79]. In colorectal cancer, evidence supports a shift in co-occurring interactions between fungi, particularly those within the *Ascomycota* phylum, and bacteria, especially those from the *Proteobacteria* phylum, towards more competitive or exclusive interactions [80]. These findings suggest that fungi may contribute to gastrointestinal carcinogenesis by altering the microbial composition of the gut [80–82]. Within *k_Bacteria | p_Actinobacteria*, the highest DOR at the class level was observed for *c_Actinobacteria* (DOR = 8.08). Studies have shown that *Actinobacteria* are more abundant in patients with PC than in healthy individuals [83]. Additionally, patients with long-term survival exhibit an enrichment of specific *Actinobacteria* species, such as *Saccharopolyspora* and *Streptomyces* [84]. At the class level, *k_Bacteria | p_Proteobacteria | c_Epsilonproteobacteria* exhibited the highest DOR of 9.45 among the oral microbiome taxa. Eun et al. reported that the *Epsilonproteobacteria* class was the most common type of bacteria in gastric cancer patients [85]. At the order level, *k_Bacteria | p_Actinobacteria | c_Actinomycetia | o_Micrococcales* exhibited the highest DOR for the oral microbiome, reaching a value of 9.33. Zhu and colleagues demonstrated that the abundance of *Micrococcales* in pancreatic tissue significantly decreased in PC patients compared with healthy individuals [86]. Among the family-level taxa for the oral microbiome, the *Lactobacillaceae* family (*p_Firmicutes (Bacillota) | c_Bacilli | o_Lactobacillales | f_Lactobacillaceae*) presented the highest DOR. The abundance of the genus *Lactobacillus*, which belongs to the *Lactobacillaceae* family, was significantly lower in patients with PC than in noncancer patients [87].

A meta-analysis of multiple oral microbiome biomarkers revealed a pooled DOR of 16.32 and an AUC of 0.86, which was considered ‘excellent’ according to the Mandrekar criterion [75]. The combined DOR for distinguishing between PC and CP was 5.78, with an AUC of 0.76. Combining multiple microbial analyses in a PC diagnostic model can enhance accuracy. Recent studies have explored the role of various microbial signatures in cancer detection, including those from the oral microbiome [41,67,68,88,89]. The pooled DOR for PC detection using mouthwash, tongue coating microbiome, and saliva were 3.45, 6.19, and 24.62, respectively. In our study, the DOR of saliva exceeded that of the tongue coating microbiome, which, in turn, outperformed mouthwash. While Belstrøm et al. found no significant differences in bacterial load between stimulated and unstimulated saliva [90], others have highlighted that the diagnostic potential may vary, with unstimulated saliva often demonstrating superior performance in detecting specific diseases and biomarkers [91–94]. However, there remains a continuing debate regarding the optimal saliva collection method. Some studies suggest stimulated saliva may enhance diagnostic sensitivity, while others report superior diagnostic performance with unstimulated saliva [93,94]. It appears that the choice of saliva type should also take into account the type of salivary biomarkers, specific disease context, and diagnostic objectives [95]. Conversely, a meta-analysis on salivary non-coding PC RNA biomarkers suggested higher DOR for stimulated saliva [93]. While both types of saliva have diagnostic potential, stimulated saliva may offer increased sensitivity because of its increased volume and altered biomarker profiles [96]. However, unstimulated saliva, which reflects the natural state of the oral microbiome, provides a baseline essential for identifying cancer-associated changes. The stability of unstimulated saliva is unaffected by external factors and enhances its diagnostic reliability [97]. The optimal type of saliva may depend on the specific cancer and biomarker. Further research is needed to refine these diagnostic methods and establish standardised protocols for clinical application.

Subgroup analyses based on bacterial taxonomy revealed that the overall DOR for PC diagnosis was highest at the phylum level and lowest at the species level. This suggests that diagnostic methods at the species level might lack sufficient robustness, potentially due to high variability within species. In contrast, genus-level identification appears to ensure a more optimal balance between specificity and sensitivity than species and family level identification, making it a more effective approach for diagnostic purposes. This is because relevant biological differences may be captured without the excessive variability often observed at the species level. In the study by Poore et al., the diagnostic accuracy for various cancers, including PC, was higher at the genus level compared to the species level. They reported that genus-level identification offers an improved balance between specificity and sensitivity [98].

At the subgenus level of taxonomy, two groups, *k_Bacteria | p_Firmicutes (Bacillota) | c_Bacilli | o_Lactobacillales | f_Streptococcaceae | g_Streptococcus* and *k_Bacteria | p_Bacteroidetes | c_Bacteroidia | o_Bacteroidales | f_Prevotellaceae | g_Prevotella*, accounted for the majority of study units. These groups not only demonstrated acceptable accuracy for diagnosing PC but also showed good differentiation between CP and PC. Pourali et al. showed that the abundance of *g_Streptococcus* is significantly higher in patients with PC [99]. This suggests *Streptococcus* may serve as a valuable noninvasive biomarker for the early diagnosis of PC. In a study that compared the salivary microbiota between PC patients and healthy controls, *Streptococcus* demonstrated high accuracy in distinguishing PC patients from healthy subjects [55]. Research also suggests that dysbiosis, or imbalance in the gut microbiota, including *Prevotella*, may contribute to the development of PC [55,100]. These bacterial genera may influence tumorigenesis through mechanisms such as inflammation and immune modulation [84,101,102].

Oral microbiome signatures should be interpreted as adjuncts to established serum biomarkers, not as replacements. CA19-9 remains widely used in the work-up of pancreatic ductal adenocarcinoma [103]. However, its performance is limited by suboptimal sensitivity for early disease, potential false positives in benign pancreatobiliary conditions, and lack of expression in Lewis antigen–negative individuals [103,104]. In this context, combining oral microbiome profiles with CA19-9 is a rational strategy to enhance overall diagnostic accuracy, particularly for risk stratification and surveillance of high-risk individuals. In our review, most primary studies did not report performance metrics for combined microbiome and CA19-9 models; therefore, our quantitative synthesis focuses on microbiome-based indices. We explicitly discuss complementarity in the clinical interpretation and suggest prospective validation of integrated panels (oral microbiome with CA19-9 liquid-biopsy markers) as a priority to determine real-world utility for early detection and triage.

Despite the compelling findings of this study, several methodological limitations should be noted. First, the heterogeneity of the studies included in our review, questionable methodology, study population definition, and covariate measurement could have influenced the overall results and reduced the precision of the estimates. Second, while the studies were assessed for their methodological quality using QUADAS-2, some studies may have inherent methodological limitations that could have affected the findings. Third, the relatively small sample sizes may have led to less reliable and precise estimates. Fourth, publication bias (higher likelihood of publication and access for studies with statistically significant outcomes) may have influenced the results of the meta-analysis [105,106]. Given these limitations, several avenues for future research can be proposed. First, standardising methods for collecting a specimen, such as saliva, mouthwash, or tongue swabs, would enhance the comparability of future studies. Second, employing advanced data analysis techniques, including artificial neural networks and machine learning, could facilitate more precise identification of microbial biomarkers associated with PC. Third, longitudinal studies in various populations could elucidate dynamic changes in the oral microbiome during disease progression and uncover underlying mechanisms.

## Conclusion

This study represents the first comprehensive systematic review and diagnostic meta-analysis assessing the diagnostic accuracy of the oral microbiome in PC detection. Our findings suggest that the oral microbiome may serve as a promising noninvasive biomarker for PC, offering a potential tool to differentiate individuals with and without the disease. Given the growing interest in microbiome-based diagnostics, further large-scale studies are warranted to validate these findings. Such studies should carefully account for various confounders, including age, race, tobacco and alcohol use, smoking, diabetes, chronic pancreatitis, pancreatic inflammation, and family history of PC. These efforts will be crucial in confirming the clinical applicability of the oral microbiome for the early detection of PC.

## Consent for publication

All authors read and approved the final manuscript.

## Supplementary Material

Supplementary material Supplementary File 1 PRISMA DTA Checklist

Supplementary material Supplementary File 2 Tables

Supplementary material Supplementary File 3 Statistical and bioinformatic analysis of studies

Supplementary material Supplementary File 4 Details of study units

Supplementary material Supplementary File 5 QUADAS 2

Supplementary material Supplementary File 6 Figures

## Data Availability

The datasets used and/or analysed during the current study are available in the Supplementary Files and Figures, and more required data will also be available from the corresponding author upon reasonable request.
