# Supplementary material for: Oral microbiome as a diagnostic biomarker for pancreatic cancer: a systematic review and meta-analysis of diagnostic accuracy
Source: J Oral Microbiol. 2025 Oct 21;17(1):2571188. doi: 10.1080/20002297.2025.2571188 (PMC12541924; doi:10.1080/20002297.2025.2571188)
Supplement: Supplementary material — Supplementary File 2 Tables [file ZJOM_A_2571188_SM9042.docx]

**Supplementary Tables Legends**

**Table S1:** Search strategy in databases and Google Scholar search engine.

**Table S2:** Moses' model (D = α + βS) for diagnostic threshold (inverse variance) of PC by oral microbiome.

**Table S3:** Subgroup meta-analysis of oral microbiota for pancreatic cancer diagnosis based on the microbiota taxonomy.

**Table S4:** Subgroup analysis of oral microbiome for pancreatic cancer diagnosis based on various covariates.

**Table S1:** Search strategy in databases and Google Scholar search engine.

| **Database** | **Search**  **(October 2023, updated in January 2024)** |
| --- | --- |
| **Cochrane library**  **(N=37)** | # 1: “saliva” OR “mouth”  # 2: “pancreatic neoplasms”  # 3: “microbiota”  # 4: # 1 AND # 2 AND # 3 |
| **Embase**  **(N=954)** | ('saliva':ab,ti OR 'oral':ab,ti OR 'salivary':ab,ti OR 'mouth':ab,ti) AND ('pancreatic':ab,ti OR 'pancreas':ab,ti) AND ('microbiome':ab,ti OR 'microbial':ab,ti OR 'bacterial':ab,ti OR 'mycobiome':ab,ti OR 'microbiota':ab,ti) |
| **LIVIVO**  **(N=32)** | saliva AND pancreatic AND microbiome  oral AND pancreatic AND microbiome |
| **MEDLINE**  **(N=373)** | (( "saliva"[Title/Abstract] ) OR ("oral"[Title/Abstract]) OR ("salivary"[Title/Abstract]) OR ("mouth"[Title/Abstract])) AND (("microbiome"[Title/Abstract]) OR ("microbiota"[Title/Abstract]) OR ("mycobiome"[Title/Abstract]) OR ("microbial"[Title/Abstract]) OR ("microbiota"[Title/Abstract]) OR ("flora"[Title/Abstract])OR ("bacteria"[Title/Abstract])) AND (("pancreatic"[Title/Abstract]) OR ("pancreas"[Title/Abstract])) |
| **OVID**  **(N=31)** | TS =(saliva AND pancreatic) |
| **Scopus**  **(N=1282)** | ( ( TITLE-ABS-KEY ( oral ) OR TITLE-ABS-KEY ( saliva ) OR TITLE-ABS-KEY ( salivary ) )  AND  ( TITLE-ABS-KEY ( microbiota ) OR TITLE-ABS-KEY ( microbiome ) OR TITLE-ABS-KEY ( mycobiome ) )  AND  (TITLE-ABS-KEY( pancreatic neoplasms ) OR ( pancreatic neoplasms ) OR ( pancreatic neoplasm ) OR ( pancreatic neoplasia ) or ( pancreatic neoplasias ) OR ( pancreatic cancer ) OR ( pancreatic cancers ) OR ( pancreas malignancy ) OR ( pancreas malignant ) OR ( malignant pancreas ) OR ( carcinoma pancreas ) OR ( pancreas carcinoma ) OR ( pancreas tumor ) OR ( pancreas tumors ) OR ( pancreatic adenocarcinoma ) OR ( pancreatic ductal adenocarcinoma ) OR ( pancreatic acinar cell carcinoma ) OR ( adenosquamous carcinoma ) OR ( pancreatic neuroendocrine tumor ) OR ( intraductal neoplasms of pancreas ) OR ( pancreas ))) |
| **Web of Science**  **(N=529)** | ((TS=(saliva) OR TS=(oral) OR TS=(salivary) OR TS=(mouth)) AND ((TS=(microbiome) OR TS=(microbiota) OR TS=(mycobiome) OR TS=(microbial) OR TS=(microbiota) OR TS=(flora) OR TS=(bacteria)) AND (TS=(pancreatic) OR TS=(pancreas)))) |
| **Google Scholar search engine**  **(N=679)** | Search 1: allintitle: oral pancreatic neoplasms  Search 2: allintitle: saliva pancreatic neoplasms  Search 3: allintitle: oral pancreatic cancer  Search 4: allintitle: saliva pancreatic cancer  Search 5: allintitle: saliva microbiota pancreatic neoplasms  Search 6: allintitle: oral microbiota pancreatic neoplasms  Search 7: allintitle: saliva microbiota pancreatic cancer  Search 8: allintitle: oral microbiota pancreatic cancer  Search 9: allintitle: saliva microbiome pancreatic neoplasms  Search 10: allintitle: oral microbiome pancreatic neoplasms  Search 11: allintitle: saliva microbiome pancreatic cancer  Search 12: allintitle: oral microbiome pancreatic cancer |

**Table S2:** Moses' model (D = α + βS) for diagnostic threshold (inverse variance) of PC by oral microbiome.

| **Variation** | **Coefficient** | **Standard Error** | **T** | **p** |
| --- | --- | --- | --- | --- |
| α | 1.553 | 0.088 | 17.618 | 0.000 |
| β | -0.146 | 0.097 | 1.497 | 0.137 |

Inverse Variance

Tau-squared estimate = 0.223 (Convergence is achieved after 20 iterations)

Restricted Maximum Likelihood estimation (REML)

**Table S3:** Subgroup meta-analysis of oral microbiota for pancreatic cancer diagnosis based on the microbiota taxonomy.

|  | **Study units** | **Case / Healthy Control** | **DOR**  **(95% CI)** | **I^2^ (%)** | **chi-squared** | **AUC**  **(95% CI)** | **Sensitivity**  **(95% CI)** | **Specificity**  **(95% CI)** | **PLR**  **(95% CI)** | **NLR**  **(95% CI)** |
| --- | --- | --- | --- | --- | --- | --- | --- | --- | --- | --- |
| **Oral microbiota (PC vs. healthy)** | **133** | **2654 / 2154** | **4.85 (4.11-5.74)** | **26.1** | **179.29** | **0.74 (0.73-0.75)** | **0.65 (0.64-0.67)** | **0.70 (0.68-0.71)** | **2.03 (1.87-2.20)** | **0.50 (0.47-0.54)** |
| ***k_Bacteria*** | **131** | **2586 / 2084** | **4.41 (3.79-5.13)** | **12.7** | **148.93** | **0.71 (0.70-0.72)** | **0.65 (0.63-0.67)** | **0.69 (0.67-0.71)** | **1.88 (1.75-2.02)** | **0.52 (0.49-0.56)** |
| **A : *p_Actinobacteria*** | **21** | **326 / 231** | **7.30 (4.83-11.02)** | **0.0** | **14.86** | **0.79 (0.76-0.82)** | **0.67 (0.62-0.72)** | **0.79 (0.73-0.84)** | **2.69 (2.10-3.46)** | **0.45 (0.38-0.53)** |
| **A : *c_Actinobacteria*** | **7** | **210 / 175** | **8.08 (4.30-15.16)** | **37.9** | **9.66** | **0.76 (0.71-0.81)** | **0.66 (0.59-0.73)** | **0.80 (0.73-0.86)** | **3.17 (2.04-4.92)** | **0.44 (0.35-0.54)** |
| ***o_Actinomycetales*** | **5** | **150 / 125** | **7.79 (3.42-17.74)** | **50.3** | **8.05** | **0.76 (0.67-0.85)** | **0.65 (0.57-0.73)** | **0.80 (0.72-0.87)** | **3.17 (1.84-5.46)** | **0.44 (0.33-0.60)** |
| ***o_Coriobacteriales*** | **1** | **30 / 25** |  |  |  |  |  |  |  |  |
| **B : *c_Coriobacteriia*** | **2** | **16 /5** | **3.73 (0.44-31.51)** | **0.0** | **0.60** | **-** | **0.56 (0.30-0.80)** | **0.80 (0.28-0.99)** | **2.04 (0.49-8.45)** | **0.59 (0.26-1.33)** |
| ***o_Coriobacteriales*** | **2** | **16 / 5** | **3.73 (0.44-31.51)** | **0.0** | **0.60** | **-** | **0.56 (0.30-0.80)** | **0.80 (0.28-0.99)** | **2.04 (0.49-8.45)** | **0.59 (0.26-1.33)** |
| **C : *c_Actinomycetia*** | **12** | **100 / 51** | **7.43 (3.29-16.79)** | **0.0** | **4.20** | **0.80 (0.75-0.85)** | **0.71 (0.61-0.80)** | **0.76 (0.62-0.87)** | **2.50 (1.60-3.19)** | **0.46 (0.33-0.64)** |
| ***o_Bifidobacteriales*** | **4** | **26 / 19** | **5.10 (1.30-19.97)** | **0.0** | **0.89** | **0.74 (0.65-0.83)** | **0.73 (0.52-19.97)** | **0.68 (0.43-0.87)** | **2.03 (1.07-3.87)** | **0.50 (0.25-0.99)** |
| ***o_Micrococcales*** | **7** | **57 / 29** | **9.33 (3.19-27.31)** | **0.0** | **2.85** | **0.83 (0.77-0.89)** | **0.75(0.62-0.86)** | **0.79 (0.60-0.92)** | **2.97 (1.57-5.64)** | **0.41 (0.25-0.65)** |
| **B : *p_Firmicutes (Bacillota)*** | **63** | **1085 / 753** | **4.76 (3.81-5.93)** | **0.0** | **52.94** | **0.73 (0.71-0.75)** | **0.66 (0.63-0.69)** | **0.73 (0.70-0.76)** | **2.03 (1.80-2.30)** | **0.50 (0.46-0.55)** |
| **A : *c_Bacilli*** | **33** | **460 / 294** | **3.42 (2.46-4.77)** | **0.0** | **17.32** | **0.69 (0.66-0.72)** | **0.63 (0.59-0.68)** | **0.69 (0.63-0.74)** | **1.79 (1.49-2.16)** | **0.58 (0.51-0.67)** |
| ***o_Bacillales*** | **3** | **24 / 6** |  |  |  |  |  |  |  |  |
| ***f_Gemella*** | **3** | **24 / 6** | **7.91 (1.25-50.18)** | **0.0** | **0.30** | **0.80 (0.69-0.91)** | **0.75 (0.53-0.90)** | **0.83 (0.36-1.00)** | **2.73 (0.82-9.09)** | **0.37 (0.17-0.80)** |
| ***o_Lactobacillales*** | **30** | **436 / 288** | **3.33 (2.38-4.66)** | **0.0** | **16.20** | **0.69 (0.66-0.72)** | **0.63 (0.58-0.67)** | **0.68 (0.63-0.74)** | **1.78 (1.47-2.14)** | **0.59 (0.51-0.68)** |
| ***f_Aerococcaceae*** | **1** | **6 / 6** |  |  |  |  |  |  |  |  |
| ***f_Carnobacteriaceae*** | **2** | **24 / 14** | **4.45 (0.96-20.64)** | **0.0** | **0.15** | **-** | **0.54 (0.33-0.74)** | **0.79 (0.49-0.95)** | **2.55 (0.88-7.41)** | **0.58 (0.34-1.00)** |
| ***f_Lactobacillaceae*** | **3** | **11 / 14** | **15.67 (2.11-116.56)** | **0.0** | **0.36** | **0.87 (0.78-0.96)** | **0.91 (0.59-1.00)** | **0.79 (0.49-0.95)** | **2.61 (1.17-5.84)** | **0.31 (0.11-0.90)** |
| ***f_Streptococcaceae*** | **24** | **395 / 254** | **3.11 (2.18-4.43)** | **0.0** | **13.10** | **0.68 (0.65-0.71)** | **0.62 (0.58-0.67)** | **0.67 (0.61-0.73)** | **1.71 (1.40-2.08)** | **0.60 (0.52-0.70)** |
| **B : *c_Clostridia*** | **20** | **426 / 358** | **7.49 (5.20-10.80)** | **0.0** | **20.23** | **0.77 (0.75-0.79)** | **0.67 (0.62-0.71)** | **0.78 (0.73-0.82)** | **2.67 (2.09-3.43)** | **0.45 (0.39-0.51)** |
| ***o_Clostridiales*** | **10** | **300 / 250** | **8.59 (4.95-14.92)** | **38.9** | **14.72** | **0.78 (0.75-0.81)** | **0.67 (0.61-0.72)** | **0.80 (0.74-0.84)** | **3.23 (2.12-4.92)** | **0.43 (0.35-0.52)** |
| ***o_Eubacteriales*** | **5** | **34 / 13** | **6.90 (1.61-29.58)** | **0.0** | **0.92** | **0.81 (0.66-0.96)** | **0.68 (0.49-0.83)** | **0.85 (0.55-0.98)** | **2.81 (1.06-7.45)** | **0.44 (0.27-0.74)** |
| ***o_Erysipelotrichales*** | **4** | **62 / 70** | **4.99 (2.27-11.00)** | **0.0** | **0.43** | **0.75 (0.70-0.80)** | **0.71 (0.58-0.82)** | **0.66 (0.53-0.77)** | **1.96 (1.39-2.78)** | **0.50 (0.34-0.74)** |
| **C : *c_Erysipelotrichia*** | **2** |  |  |  |  |  |  |  |  |  |
| ***o_Erysipelotrichales*** | **2** | **37 / 29** | **2.97 (1.08-8.19)** | **0.0** | **0.02** | **-** | **0.65 (0.47-0.80)** | **0.62 (0.42-0.79)** | **1.68 (1.00-2.82)** | **0.57 (0.34-0.96)** |
| **D : *c_Negativicutes*** | **6** | **121 / 52** | **3.83 (1.85-7.93)** | **0.0** | **4.12** | **0.71 (0.66-0.76)** | **0.67 (0.58-0.75)** | **0.67 (0.53-0.80)** | **1.82 (1.22-2.71)** | **0.48 (0.34-0.68)** |
| ***o_Clostridiales*** | **5** | **91 / 27** | **4.14 (1.57-10.91)** | **2.0** | **4.08** | **0.54 (0.38-0.70)** | **0.66 (0.55-0.75)** | **0.74 (0.54-0.89)** | **1.91 (1.05-3.49)** | **0.46 (0.30-0.72)** |
| ***o_Selenomonadales*** | **1** | **30 / 25** |  |  |  |  |  |  |  |  |
| **E : *c_Tissierellia*** | **1** | **4 / 2** |  |  |  |  |  |  |  |  |
| **C : *p_Bacteroidetes*** | **29** | **497 / 464** | **3.34 (2.54-4.39)** | **0.0** | **16.70** | **0.69 (0.67-0.71)** | **0.66 (0.62-0.70)** | **0.63 (0.59-0.68)** | **1.75 (1.53-2.01)** | **0.57 (0.49-0.65)** |
| ***c_Bacteroidia*** | **29** | **497 / 464** |  |  |  |  |  |  |  |  |
| ***A : o_Bacteroidales*** | **29** | **497 / 464** |  |  |  |  |  |  |  |  |
| ***B : f_Prevotellaceae*** | **14** | **324 / 287** | **2.81 (2.01-3.93)** | **0.0** | **9.52** | **0.67 (0.64-0.71)** | **0.63 (0.57-0.68)** | **0.63 (0.57-0.69)** | **1.66 (1.39-1.97)** | **0.61 (0.52-0.72)** |
| ***g_Paraprevotella*** | **2** | **44 / 41** | **3.77 (1.53-9.30)** | **0.0** | **0.01** | **-** | **0.64 (0.48-78)** | **0.68 (0.52-0.82)** | **2.01 (1.21-3.32)** | **0.53 (0.34-0.83)** |
| ***g_Prevotella*** | **11** | **220 / 196** | **2.35 (1.57-3.50)** | **0.0** | **3.99** | **0.64 (0.61-0.67)** | **0.60 (0.53-0.66)** | **0.62 (0.54-0.69)** | **1.51 (1.23-1.87)** | **0.66 (0.54-0.80)** |
| ***C : f_Porphyromonadaceae*** | **3** | **90 / 75** | **5.02 (2.55-9.87)** | **0.0** | **0.62** | **0.72 (0.66-0.78)** | **0.74 (0.64-0.83)** | **0.63 (0.51-0.74)** | **1.98 (1.45-2.71)** | **0.43 (0.29-0.63)** |
| ***D : f_Rikenellaceae*** | **2** | **5 / 14** | **5.57 (0.62-49.96)** | **0.0** | **0.01** | **-** | **0.80 (0.28-0.99)** | **0.64 (0.35-0.87)** | **2.02 (0.93-4.42)** | **0.41 (0.10-1.66)** |
| ***g_Alistipes*** | **2** | **5 / 14** | **5.57 (0.62-49.96)** | **0.0** | **0.01** | **-** | **0.80 (0.28-0.99)** | **0.64 (0.35-0.87)** | **2.02 (0.93-4.42)** | **0.41 (0.10-1.66)** |
| ***E : f_Bacteroidaceae*** | **7** |  | **2.95 (1.26-6.93)** | **0.0** | **1.22** | **0.68 (0.61-0.75)** | **0.65 (0.50-0.79)** | **0.60 (0.47-0.73)** | **1.58 (1.09-2.28)** | **0.60 (0.39-0.93)** |
| ***g_Bacteroides*** | **7** | **46 / 58** | **2.95 (1.26-6.93)** | **0.0** | **1.22** | **0.68 (0.61-0.75)** | **0.65 (0.50-0.79)** | **0.60 (0.47-0.73)** | **1.58 (1.09-2.28)** | **0.60 (0.39-0.93)** |
| ***F : f_Tannerellaceae*** | **1** |  |  |  |  |  |  |  |  |  |
| **D : *p_Fusobacteria*** | **3** | **90 / 75** | **4.97 (1.99-12.37)** | **41.2** | **3.40** | **0.75 (0.74-0.76)** | **0.73 (0.63-0.82)** | **0.63 (0.51-0.74)** | **1.90 (1.24-2.90)** | **0.43 (0.21-0.72)** |
| **E : *p_Proteobacteria*** | **11** | **259 / 213** | **5.15 (3.42-7.74)** | **0.00** | **5.61** | **0.76 (0.73-0.79)** | **0.64 (0.57-0.70)** | **0.74 (0.68-0.80)** | **2.39 (1.87-3.05)** | **0.52 (0.43-0.62)** |
| ***c_Gammaproteobacteria*** | **9** | **199 / 163** | **4.34 (2.74-6.89)** | **0.0** | **3.20** | **0.73 (0.68-0.78)** | **0.60 (0.53-0.67)** | **0.74 (0.67-0.81)** | **2.23 (1.68-2.96)** | **0.55 (0.46-0.67)** |
| ***c_Epsilonproteobacteria*** | **2** | **60 / 50** | **9.45 (3.94-22.65)** | **0.0** | **0.04** | **-** | **0.77 (0.64-0.87)** | **0.74 (0.60-0.85)** | **2.94 (1.81-4.79)** | **0.32 (0.20-0.52)** |
| **F : *p_Pseudomonadot*** | **1** |  |  |  |  |  |  |  |  |  |
| **G : *p_Saccharibacteria*** | **1** |  |  |  |  |  |  |  |  |  |
| **H : *p_Spirochaetes*** | **2** | **303 / 310** | **1.69 (0.85-3.34)** | **44.2** | **1.79** | **-** | **0.52 (0.46-0.58)** | **0.57 (0.51-0.63)** | **1.27 (0.96-1.67)** | **0.76 (0.52-1.01)** |
| ***k_Fungi*** | **2** |  |  |  |  |  |  |  |  |  |
| **I : *p_Ascomycota*** | **2** | **68 / 70** | **116.43 (34.15-392.72)** | **0.00** | **0.01** | **-** | **0.90 (0.80-0.96)** | **0.93 (0.84-0.98)** | **12.31 (5.28-28.73)** | **0.11 (0.05-0.23)** |

CI: confidence interval; PLR: positive likelihood ratio; NLR: negative likelihood ratio; DOR: diagnostic odds ratio; AUC: area under the ROC curve, HC: healthy controls; BC: benign controls, I^2^: Inconsistency (I-square), chi-squared: Heterogeneity.

**Table S4:** Subgroup analysis of oral microbiome for pancreatic cancer diagnosis based on various covariates.

|  | **Study units** | **Case / Control** | **DOR**  **(95% CI)** | **I^2^ (%)** | **chi-squared** | **AUC**  **(95% CI)** | **Sensitivity**  **(95% CI)** | **Specificity**  **(95% CI)** | **PLR**  **(95% CI)** | **NLR**  **(95% CI)** |
| --- | --- | --- | --- | --- | --- | --- | --- | --- | --- | --- |
| **Multiple oral microbiome** | **7** | **236 / 626** | **16.32 (8.37-31.86)** | **53.5** | **12.90** | **0.86 (0.84-0.88)** | **0.81 (0.75-0.86)** | **0.78 (0.74-0.81)** | **3.71 (2.74-5.03)** | **0.26 (0.18-0.38)** |
| ***China*** | **3** | **114 / 296** | **17.67 (9.79-31.92)** | **0.0** | **0.78** | **0.88 (0.86-0.90)** | **0.82 (0.73-0.88)** | **0.81 (0.76-0.85)** | **4.20 (3.23-5.45)** | **0.24 (0.16-0.36)** |
| ***USA*** | **2** | **36 / 50** | **190.49 (27.39-1325.0)** | **0.0** | **0.61** | **-** | **0.97 (0.85-1.00)** | **0.90 (0.78-0.97)** | **10.27 (1.45-72.90)** | **0.50 (0.10-0.23)** |
| **Sampling method (Type of oral samples)** |  |  |  |  |  |  |  |  |  |  |
| ***Saliva*** | **3** | **341 / 355** | **24.62 (0.67-908.07)** | **96.0** | **49.54** | **0.86 (0.59-1.00)** | **0.58 (0.53-0.64)** | **0.64 (0.59-0.69)** | **5.43 (0.67-43.85)** | **0.23 (0.4-1.38)** |
| ***Tongue coating microbiome*** | **40** | **1200 / 1000** | **6.19 (4.97-7.72)** | **18.6** | **47.93** | **0.77 (0.76-0.78)** | **0.68 (0.66-0.71)** | **0.73 (0.70-0.76)** | **2.37 (2.07-2.70)** | **0.45 (0.41-0.49)** |
| ***Mouthwash*** | **90** | **1113 / 799** | **3.45 (2.81-4.23)** | **0.0** | **43.05** | **0.70 (0.69-0.71)** | **0.64 (0.61-0.67)** | **0.67 (0.64-0.70)** | **1.78 (1.59-1.98)** | **0.57 (0.52-0.62)** |
| **Bacterial taxonomy** |  |  |  |  |  |  |  |  |  |  |
| ***Phylum level*** | **4** | **120 / 100** | **8.86 (4.17-16.64)** | **0.0** | **0.03** | **0.82 (0.79-0.85)** | **0.77 (0.68-0.84)** | **0.71 (0.61-0.80)** | **2.47 (1.82-3.37)** | **0.34 (0.24-0.47)** |
| ***Class level*** | **7** | **210 / 175** | **8.26 (4.72-14.46)** | **17.7** | **7.29** | **0.79 (0.75-0.83)** | **0.68 (0.61-0.74)** | **0.79 (0.72-0.85)** | **2.95 (1.99-4.36)** | **0.42 (0.34-0.52)** |
| ***Order level*** | **9** | **120 / 100** | **7.31 (4.44-12.05)** | **29.6** | **11.37** | **0.79 (0.76-0.82)** | **0.71 (0.65-0.76)** | **0.74 (0.68-0.80)** | **2.58 (1.97-3.37)** | **0.40 (0.32-0.51)** |
| ***Family level*** | **13** | **390 / 325** | **5.69 (3.88-8.34)** | **21.2** | **15.23** | **0.75 (0.72-0.78)** | **0.67 (0.62-0.72)** | **0.73 (0.68-0.78)** | **2.34 (1.85-2.96)** | **0.46 (0.39-0.55)** |
| ***Genus level*** | **24** | **905 / 811** | **6.68 (4.11-10.87)** | **73.9** | **88.16** | **0.77 (0.74-0.80)** | **0.64 (0.61-0.68)** | **0.68 (0.65-0.71)** | **2.42 (1.90-3.09)** | **0.43 (0.35-0.53)** |
| ***Species level*** | **87** | **1089 / 793** | **3.41 (2.77-4.19)** | **0.0** | **41.96** | **0.69 (0.68-0.70)** | **0.64 (0.61-0.67)** | **0.67 (0.64-0.70)** | **1.77 (1.59-1.97)** | **0.57 (0.52-0.63)** |
| **Subgenus-level taxonomy (PC vs. healthy control)** | **35** |  |  |  |  |  |  |  |  |  |
| ***(k_Bacteria \| p_Firmicutes (Bacillota) \| c_Bacilli \| o_Lactobacillales \| f_Streptococcaceae \| g_Streptococcus)*** | **24** | **395 / 254** | **3.11 (2.18-4.43)** | **0.0** | **13.10** | **0.68 (0.65-0.71)** | **0.62 (0.58-0.67)** | **0.67 (0.61-0.73)** | **1.71 (1.40-0.98)** | **0.60 (0.52-0.70)** |
| ***(k_Bacteria \| p_Bacteroidetes \| c_Bacteroidia \| o_Bacteroidales \| f_Prevotellaceae \| g_Prevotella)*** | **11** | **220 / 196** | **2.35 (1.57-3.50)** | **0.0** | **3.99** | **0.64 (0.61-0.67)** | **0.60 (0.53-0.66)** | **0.62 (0.54-0.69)** | **1.51 (1.23-1.87)** | **0.66 (0.54-0.80)** |
| **Pancreatic cancer (PC) versus chronic pancreatitis (CP)** | **48** | **704 / 227** | **5.78 (4.06-8.23)** | **0.0** | **18.13** | **0.76 (0.74-0.78)** | **0.65 (0.62-0.69)** | **0.77 (0.71-0.82)** | **2.22 (1.80-2.75)** | **0.49 (0.43-0.55)** |
| **Subgenus-level taxonomy (PC vs. CP)** | **22** |  |  |  |  |  |  |  |  |  |
| ***(k_Bacteria \| p_Firmicutes (Bacillota) \| c_Bacilli \| o_Lactobacillales \| f_Streptococcaceae \| g_Streptococcus)*** | **16** | **310 / 99** | **5.55 (3.23-9.54)** | **0.0** | **8.78** | **0.76 (0.72-0.80)** | **0.64 (0.59-0.70)** | **0.76 (0.66-0.84)** | **2.22 (1.59-3.11)** | **0.49 (0.41-0.59)** |
| ***(k_Bacteria \| p_Bacteroidetes \| c_Bacteroidia \| o_Bacteroidales \| f_Prevotellaceae \| g_Prevotella)*** | **6** | **97 / 29** | **6.77 (2.47-18.58)** | **0.0** | **1.67** | **0.80 (0.74-0.86)** | **0.65 (0.55-0.74)** | **0.79 (0.60-0.92)** | **2.43 (1.30-4.54)** | **0.49 (0.36-0.67)** |

CI: confidence interval; PLR: positive likelihood ratio; NLR: negative likelihood ratio; DOR: diagnostic odds ratio; AUC: area under the ROC curve, HC: healthy controls; BC: benign controls, I2: Inconsistency (I-square), chi-squared: Heterogeneity
