# Supplementary material for: Oral microbiome as a diagnostic biomarker for pancreatic cancer: a systematic review and meta-analysis of diagnostic accuracy
Source: J Oral Microbiol. 2025 Oct 21;17(1):2571188. doi: 10.1080/20002297.2025.2571188 (PMC12541924; doi:10.1080/20002297.2025.2571188)
Supplement: Supplementary material — Supplementary File 6 Figures [file ZJOM_A_2571188_SM9041.pdf]

Supplementary File 6 - (Figures)

Supplementary Figures

Fig S1: Forest plot of meta-analysis of single oral microbiome in diagnosis of pancreatic cancer (PC) versus healthy controls.

Fig S2: Hierarchical summary ROC (HSROC) curve considering the sample size of study units.

Fig S3: Likelihood matrix of the meta-analysis of single oral microbiome in PC diagnosis.

Fig S4: Deeks' funnel plot asymmetry test for publication bias in meta-analysis of PC diagnosis using single oral microbiome.

Fig S5: Oral microbiome classification used in the subgroup meta-analysis of PC diagnosis

Fig S6: Forest plot of subgroup meta-analysis of oral microbiome in the diagnosis of PC using phylum classification.

Fig S7: SROC curve of oral microbiome biomarkers for PC diagnosis based on phylum-level.

Fig S8: Forest plot meta-analysis of oral microbiome (*k_Bacteria* | *p_Actinobacteria*) in the diagnosis of PC versus healthy controls.

Fig S9: SROC curve of oral microbiota (class family of *k_Bacteria* | *p_Actinobacteria*) in the diagnosis of PC versus healthy controls using various colors for each study.

Fig S10: Deeks' funnel plot asymmetry test for publication bias in the meta-analysis of PC diagnosis using oral microbiome (*k_Bacteria* | *p_Actinobacteria*).

Fig S11: Forest plot of subgroup meta-analysis of oral microbiome (*k_Bacteria* | *p_Firmicute*s (*Bacillota*)) in the diagnosis of PC.

Fig S12: SROC curve of oral microbiota (class family of *k_Bacteria* | *p_Firmicutes* (*Bacillota*)) in diagnosis of PC versus healthy controls.

Fig S13: Deeks' funnel plot asymmetry test for publication bias in meta-analysis of PC diagnosis using oral microbiome (*k_Bacteria* | *p_Firmicutes* (*Bacillota*)).

Fig S14: Forest plot of subgroup meta-analysis of oral microbiome (*k_Bacteria* | *p_Bacteroidetes*) in the diagnosis of PC.

Fig S15: SROC curve of oral microbiota (class family of *k_Bacteria* | *p_Bacteroidetes*) in diagnosis of PC versus healthy controls.

Fig S16: Deeks' funnel plot asymmetry test for publication bias in the meta-analysis of PC diagnosis using oral microbiome (class family of *k_Bacteria* | *p_Bacteroidetes*).

Fig S17: SROC curve of oral microbiota (class family of *k_Bacteria* | *p_Fusobacteri*a) in diagnosis of PC versus healthy controls.

Fig S18: Forest plot of meta-analysis of oral microbiome (*k_Bacteria* | *p_Fusobacteria*) in the diagnosis of PC.

Fig S19: Forest plot of subgroup meta-analysis of oral microbiome (*k_Bacteria* | *p_Proteobacteria*) in the diagnosis of PC.

Fig S20: SROC curve of oral microbiota (class family of *k_Bacteria* | *p_Proteobacteria*) in diagnosis of PC versus healthy controls considering study weights of study units.

Fig S21: Deeks' funnel plot asymmetry test for publication bias in meta-analysis of PC diagnosis using oral microbiome (class family of *k_Bacteria* | *p_Proteobacteria*).

Fig S22: Forest plot of the subgroup meta-analysis of oral microbiome (multiple oral microbiome) and country in the diagnosis of PC.

Fig S23: SROC curve of subgroup meta-analysis of multiple oral microbiome for PC diagnosis.

Fig S24: Deeks' funnel plot asymmetry test for publication bias in meta-analysis of PC diagnosis using multiple oral microbiome.

Fig S25: Forest plot of the subgroup meta-analysis of oral sampling method in the diagnosis of PC.

Supplementary Figures

Fig S26: SROC curve of mouthwash saliva in the diagnosis of PC.

Fig S27: SROC curve of tongue coating in the diagnosis of PC.

Fig S28: Bacterial taxonomy in the PC diagnosis

Fig S29: Diagnostic odds ratio for the subgroup meta-analysis of PC diagnosis using bacterial taxonomy.

Fig S30: Forest plot of the meta-analysis of oral microbiome in the diagnosis of PC using phylum-levels.

Fig S31: SROC curve of the meta-analysis of oral microbiome in the diagnosis of PC using phylum-levels.

Fig S32: Forest plot of the meta-analysis of oral microbiome in the diagnosis of PC using class-levels.

Fig S33: SROC curve of the meta-analysis of oral microbiome in the diagnosis of PC using class-levels.

Fig S34: Forest plot of the meta-analysis of oral microbiome in the diagnosis of PC using order-levels.

Fig S35: SROC curve of the meta-analysis of oral microbiome in the diagnosis of PC using order-levels.

Fig S36: Forest plot of the meta-analysis of oral microbiome in the diagnosis of PC using family-levels.

Fig S37: SROC curve of the meta-analysis of oral microbiome in the diagnosis of PC using family-levels.

Fig S38: Forest plot of the meta-analysis of oral microbiome in the diagnosis of PC using genus-levels.

Fig S39: SROC curve of the meta-analysis of oral microbiome in the diagnosis of PC using genus-levels.

Fig S40: Forest plot of the meta-analysis of oral microbiome in the diagnosis of PC using species-levels.

Fig S41: SROC curve of the meta-analysis of oral microbiome in the diagnosis of PC using species-levels.

Fig S42: Forest plot of the meta-analysis of oral microbiome in the diagnosis of PC using subgenus-level taxonomy (*g_Streptococcus*).

Fig S43: SROC curve of the meta-analysis of oral microbiome in the diagnosis of PC using subgenus-level taxonomy (*g_Streptococcus*).

Fig S44: Forest plot of the meta-analysis of oral microbiome in the diagnosis of PC using subgenus-level taxonomy (*g_Prevotella*).

Fig S45: SROC curve of the meta-analysis of oral microbiome in the diagnosis of PC using subgenus-level taxonomy (*g_Prevotella*).

Fig S46: Forest plot of the meta-analysis of single oral microbiome in diagnosis of pancreatic cancer (PC) versus chronic pancreatitis (CP).

Fig S47: SROC curve of the meta-analysis of oral microbiome in the diagnosis of PC versus chronic pancreatitis (CP).

Fig S48: Forest plot of the meta-analysis of single oral microbiome (*g_Streptococcus*) in diagnosis of pancreatic cancer (PC) versus chronic pancreatitis (CP).

Fig S49: SROC curve of the meta-analysis of oral microbiome (*g_Streptococcus*) in the diagnosis of PC versus chronic pancreatitis (CP).

Fig S50: Forest plot of the meta-analysis of oral microbiome (*g_Prevotella*) in diagnosis of pancreatic cancer (PC) versus chronic pancreatitis (CP).

Fig S51: SROC curve of the meta-analysis of oral microbiome (*g_Prevotella*) in the diagnosis of PC versus chronic pancreatitis (CP).

Pancreatic cancer (PC) versus healthy control

using single oral microbiome

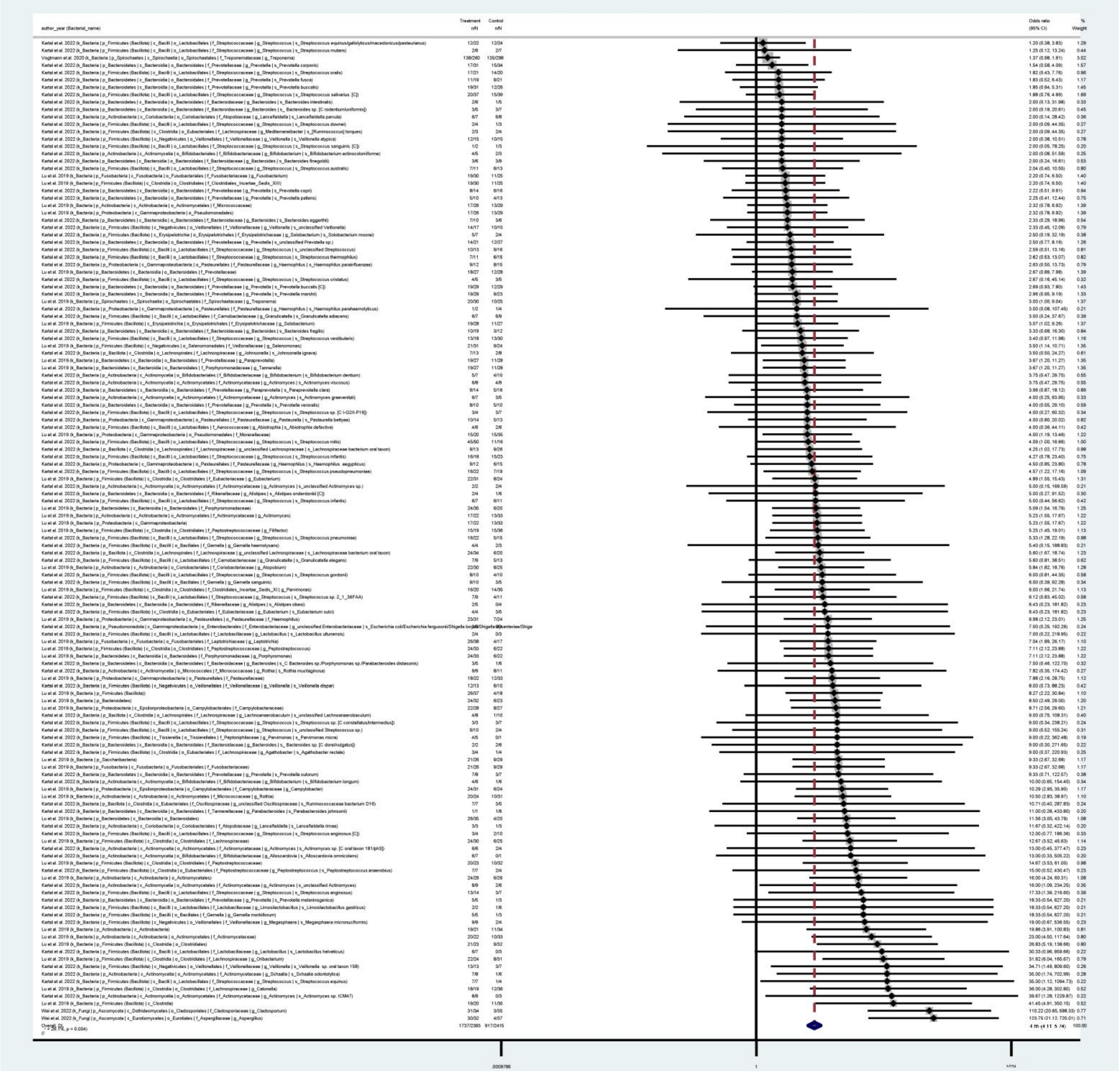

Fig S1: Forest plot of meta-analysis of single oral microbiome in diagnosis of pancreatic cancer (PC) versus healthy controls.

Pancreatic cancer (PC) versus healthy control, using single oral microbiome

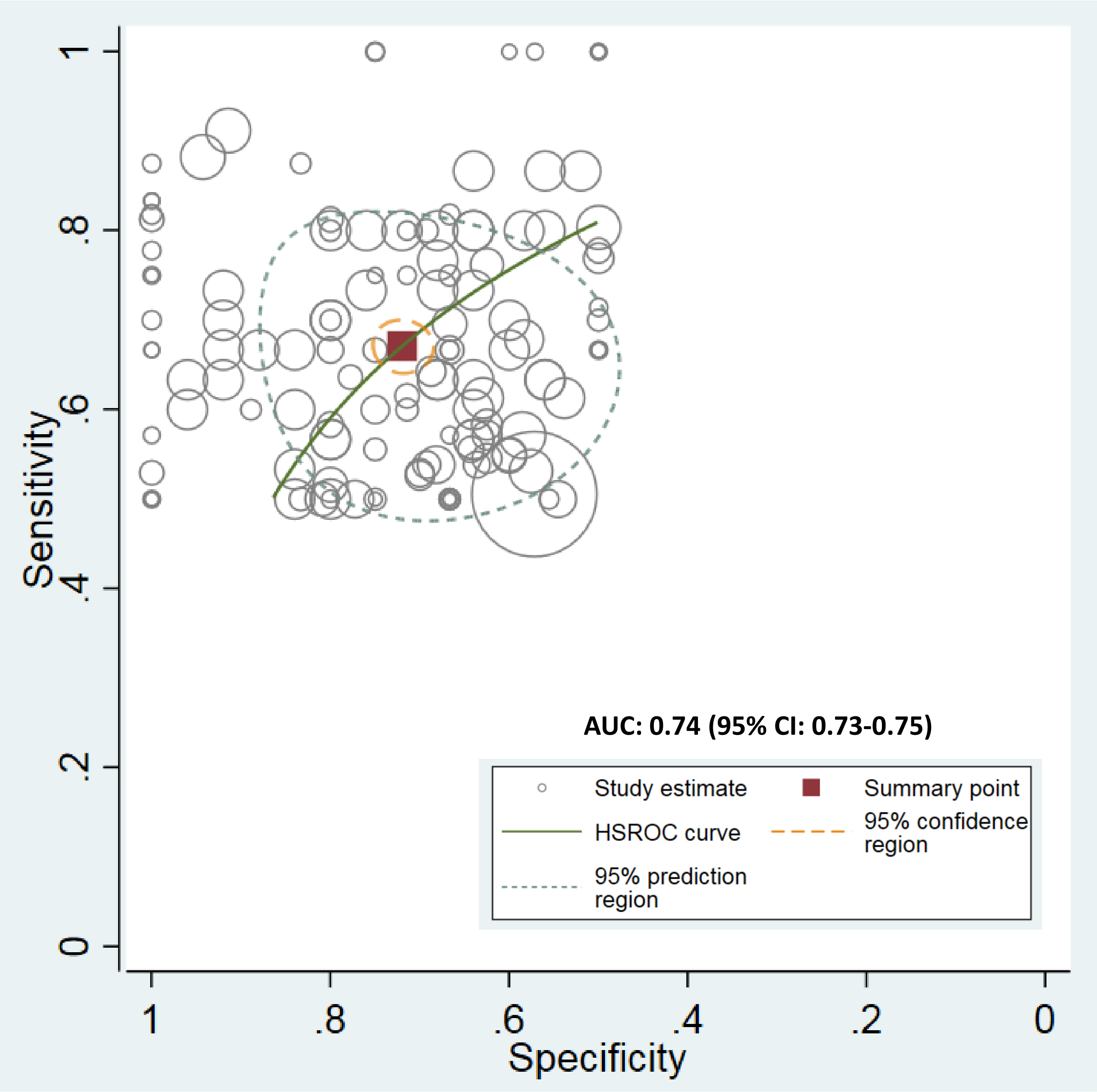

Fig S2: Hierarchical summary ROC (HSROC) curve considering the sample size of study units.

Pancreatic cancer (PC) versus healthy control, using single oral microbiome

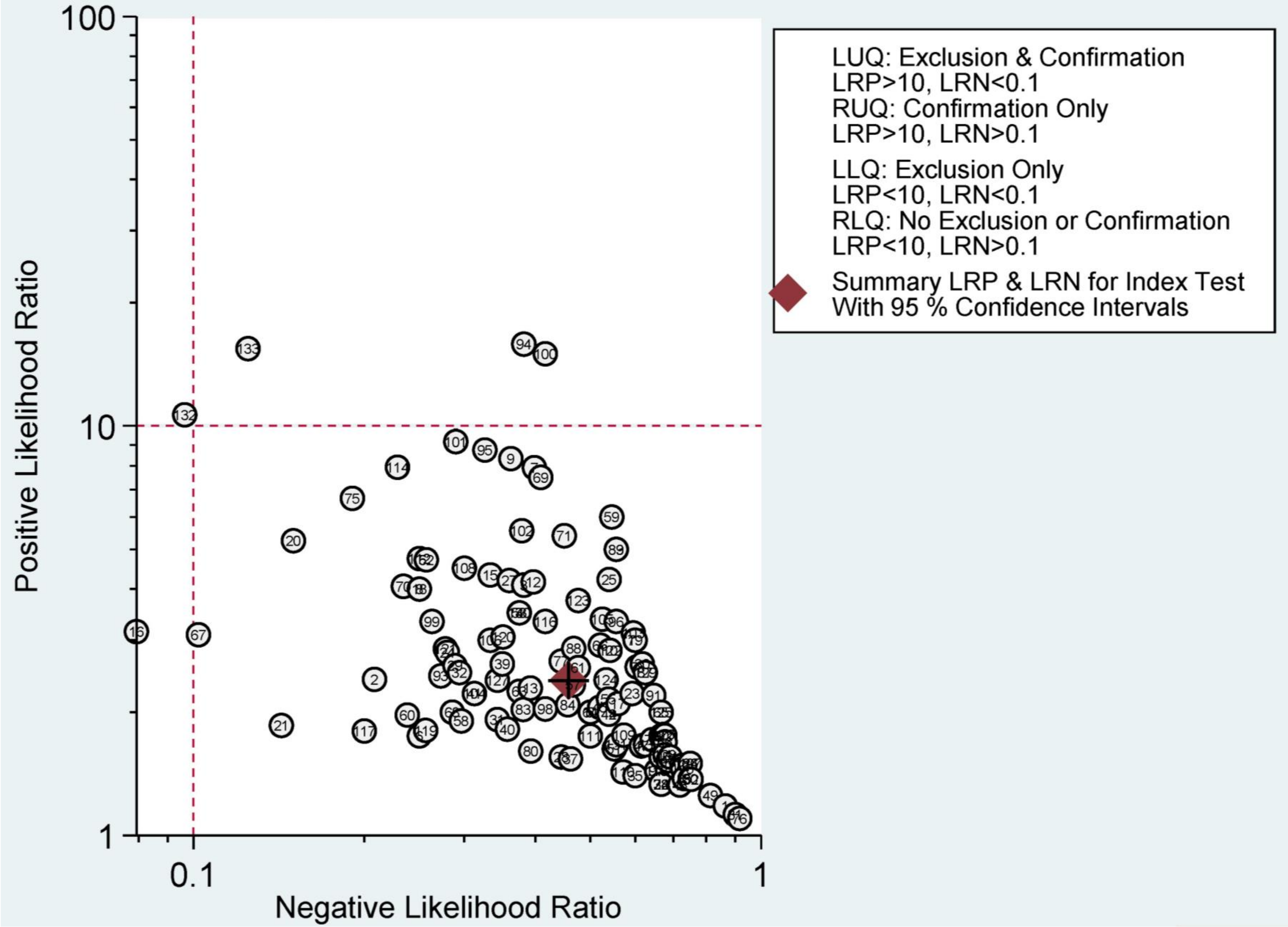

Fig S3: Likelihood matrix of the meta-analysis of single oral microbiome in PC diagnosis.

Pancreatic cancer (PC) versus healthy control, using single oral microbiome

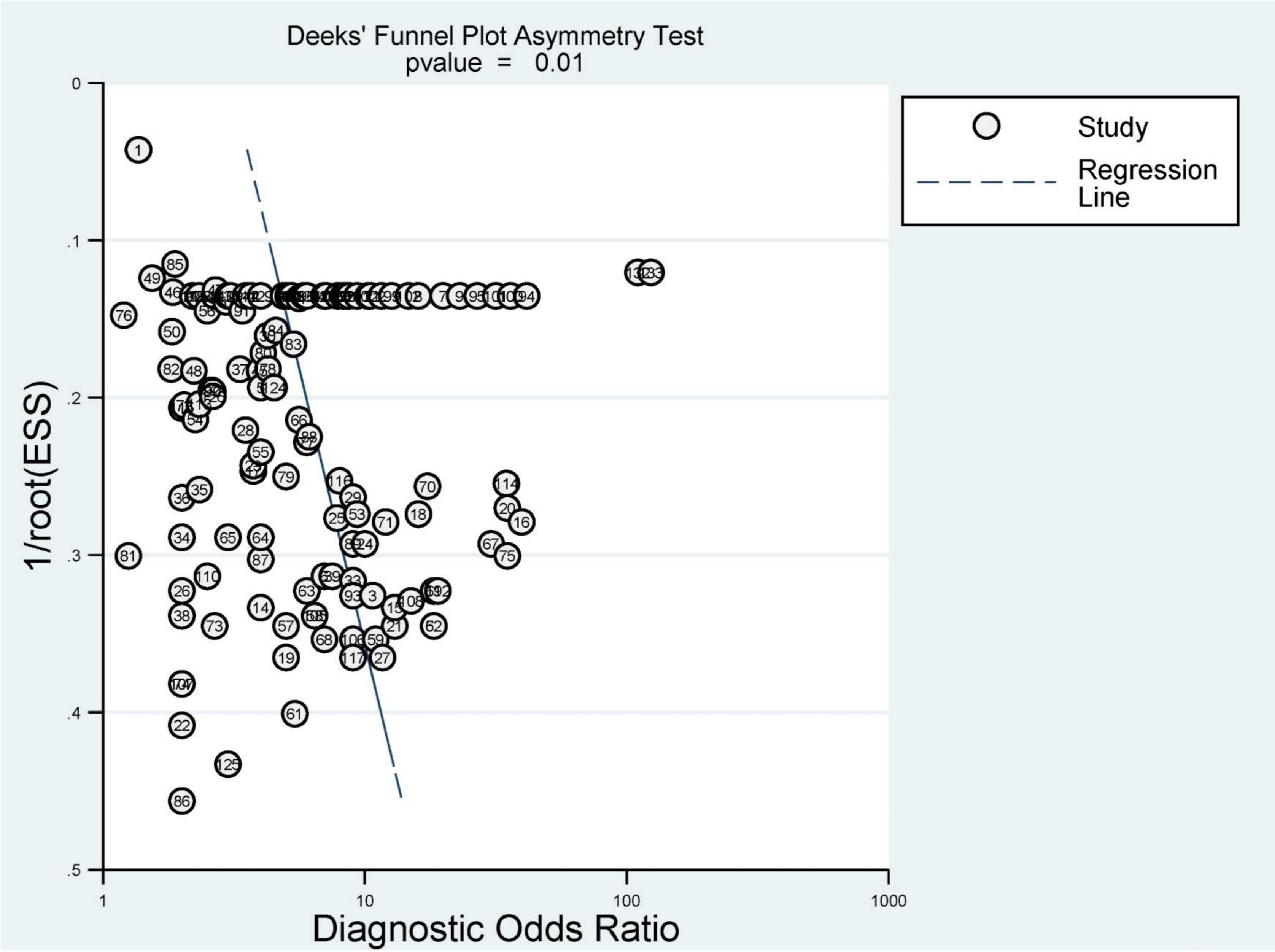

Fig S4: Deeks' funnel plot asymmetry test for publication bias in meta-analysis of PC diagnosis using single oral microbiome.

Oral microbiome classification

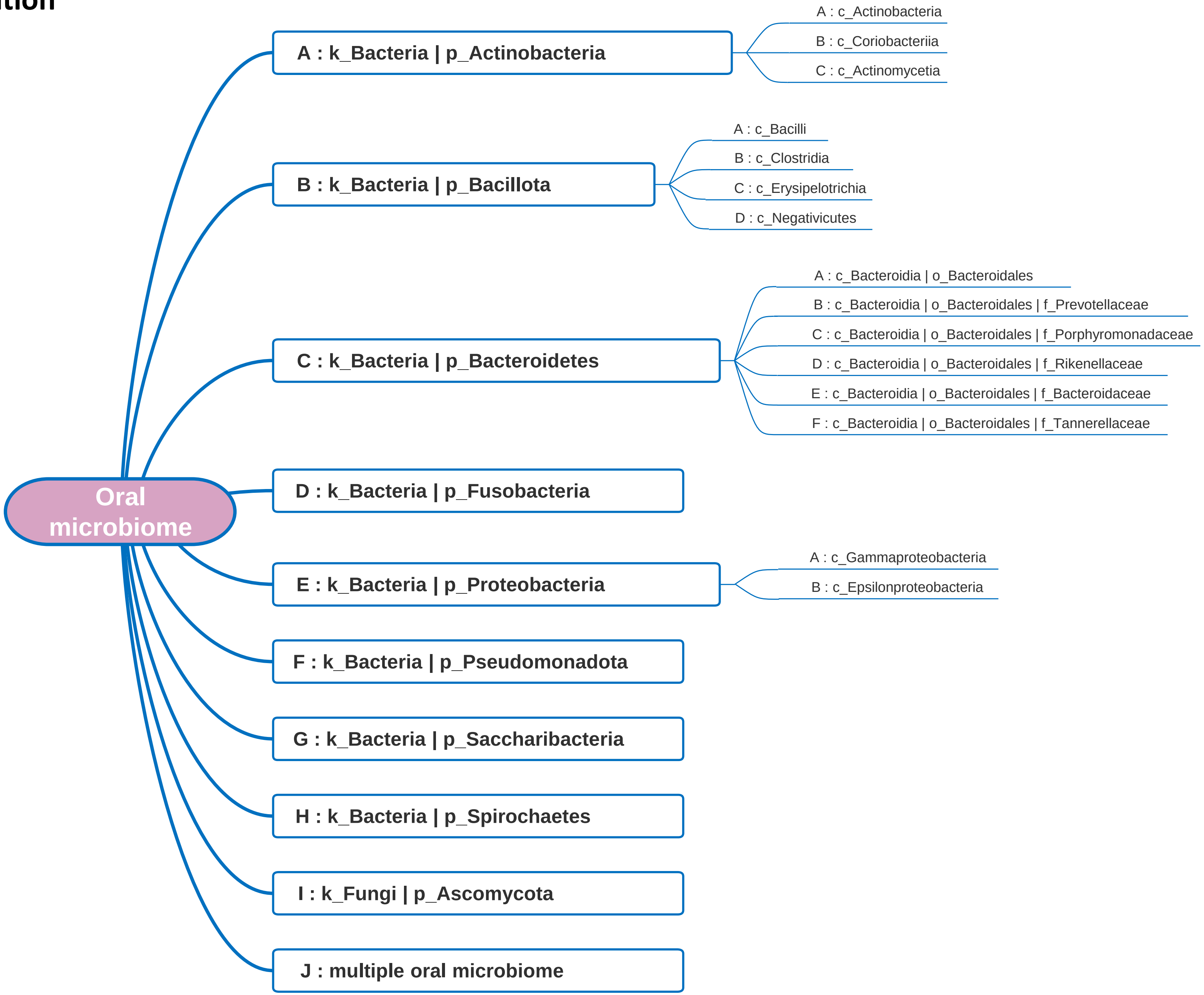

Fig S5: Oral microbiome classification used in the subgroup meta-analysis of PC diagnosis

Subgroup meta-analysis (phylum classification of bacteria)
Pancreatic cancer versus healthy control

A : k_Bacteria | p_Actinobacteria

B : k_Bacteria | p_Bacillota

C : k_Bacteria | p_Bacteroidetes

D : k_Bacteria | p_Fusobacteria

E : k_Bacteria | p_Proteobacteria

F : k_Bacteria | p_Pseudomonadot

G : k_Bacteria | p_Saccharibacteria

H : k_Bacteria | p_Spirochaetes

I : k_Fungi | p_Ascomycota

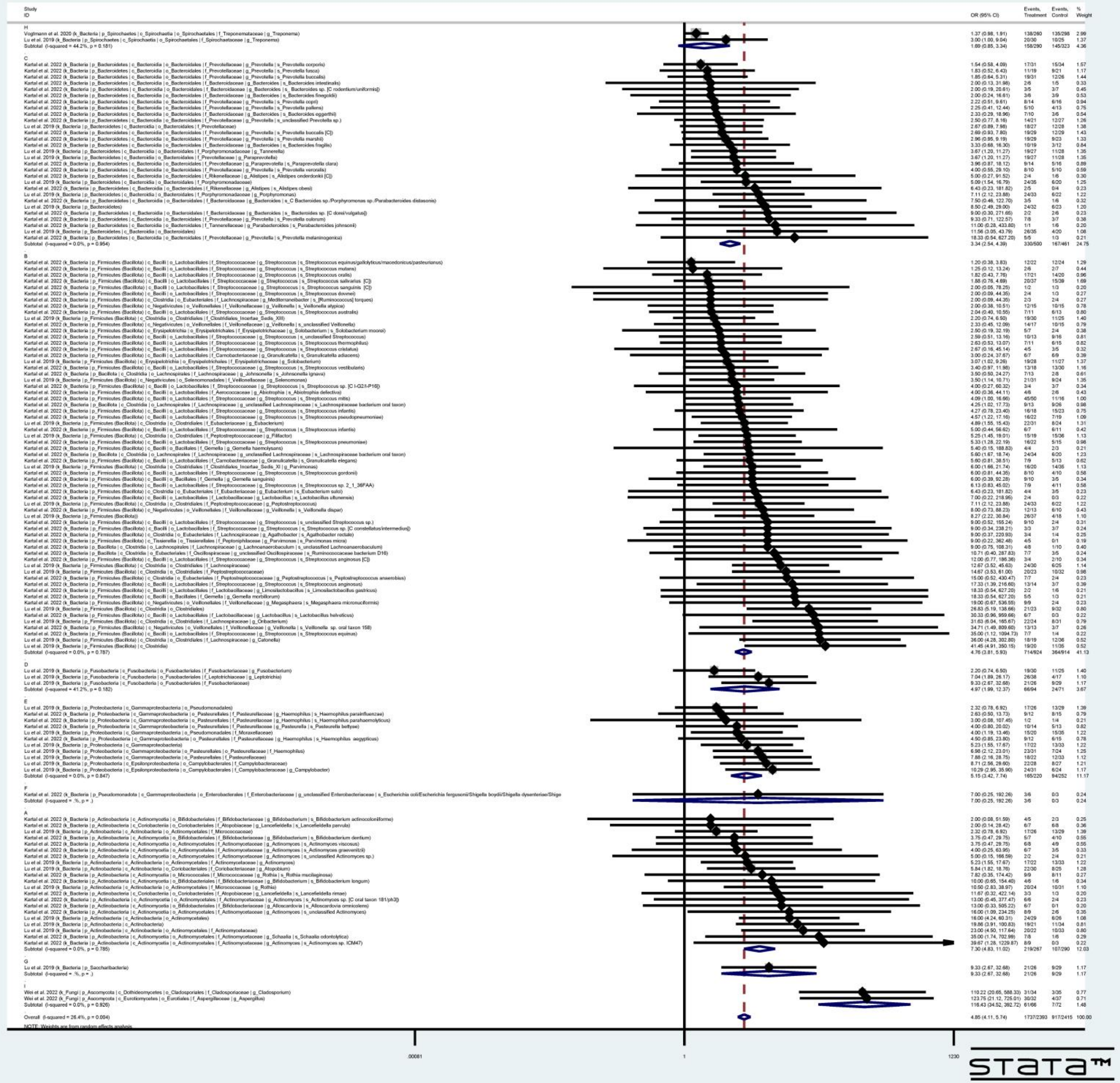

Fig S6: Forest plot of subgroup meta-analysis of oral microbiome in the diagnosis of PC using phylum classification.

Subgroup meta-analysis (phylum classification of bacteria)

Pancreatic cancer versus healthy control

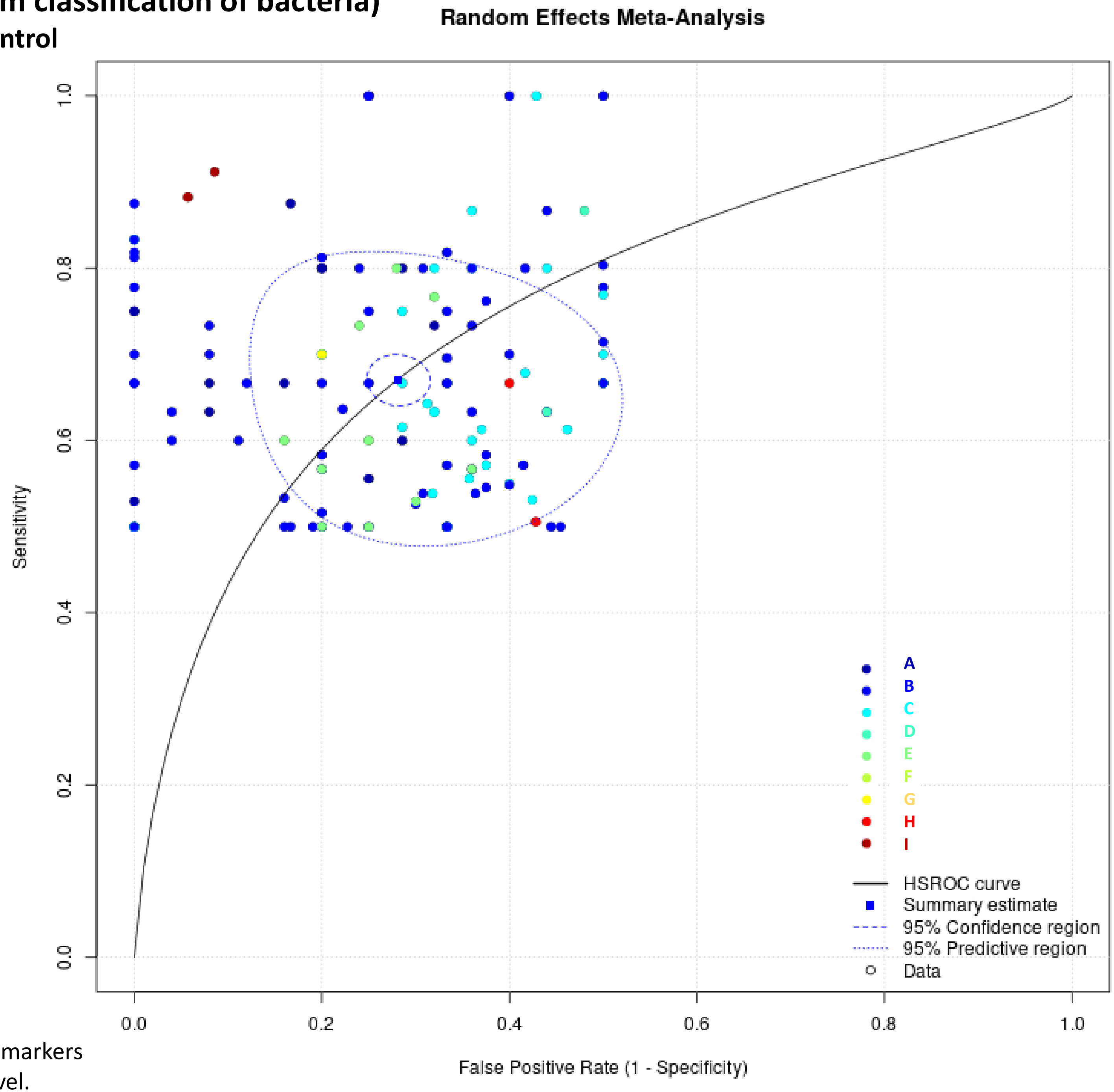

A : k_Bacteria | p_Actinobacteria

B : k_Bacteria | p_Bacillota

C : k_Bacteria | p_Bacteroidetes

D : k_Bacteria | p_Fusobacteria

E : k_Bacteria | p_Proteobacteria

F : k_Bacteria | p_Pseudomonadot

G : k_Bacteria | p_Saccharibacteria

H : k_Bacteria | p_Spirochaetes

I : k_Fungi | p_Ascomycota

Fig S7: SROC curve of oral microbiome biomarkers for PC diagnosis based on phylum-level.

k_Bacteria | p_Actinobacteria

A : c_Actinobacteria

(1 : o_Actinomycetales)

(2 : o_Coriobacteriales)

B : c_Coriobacteriia

(1 : o_Coriobacteriales)

C : c_Actinomycetia

(1 : o_Micrococcales)

(2 : o_Bifidobacteriales)

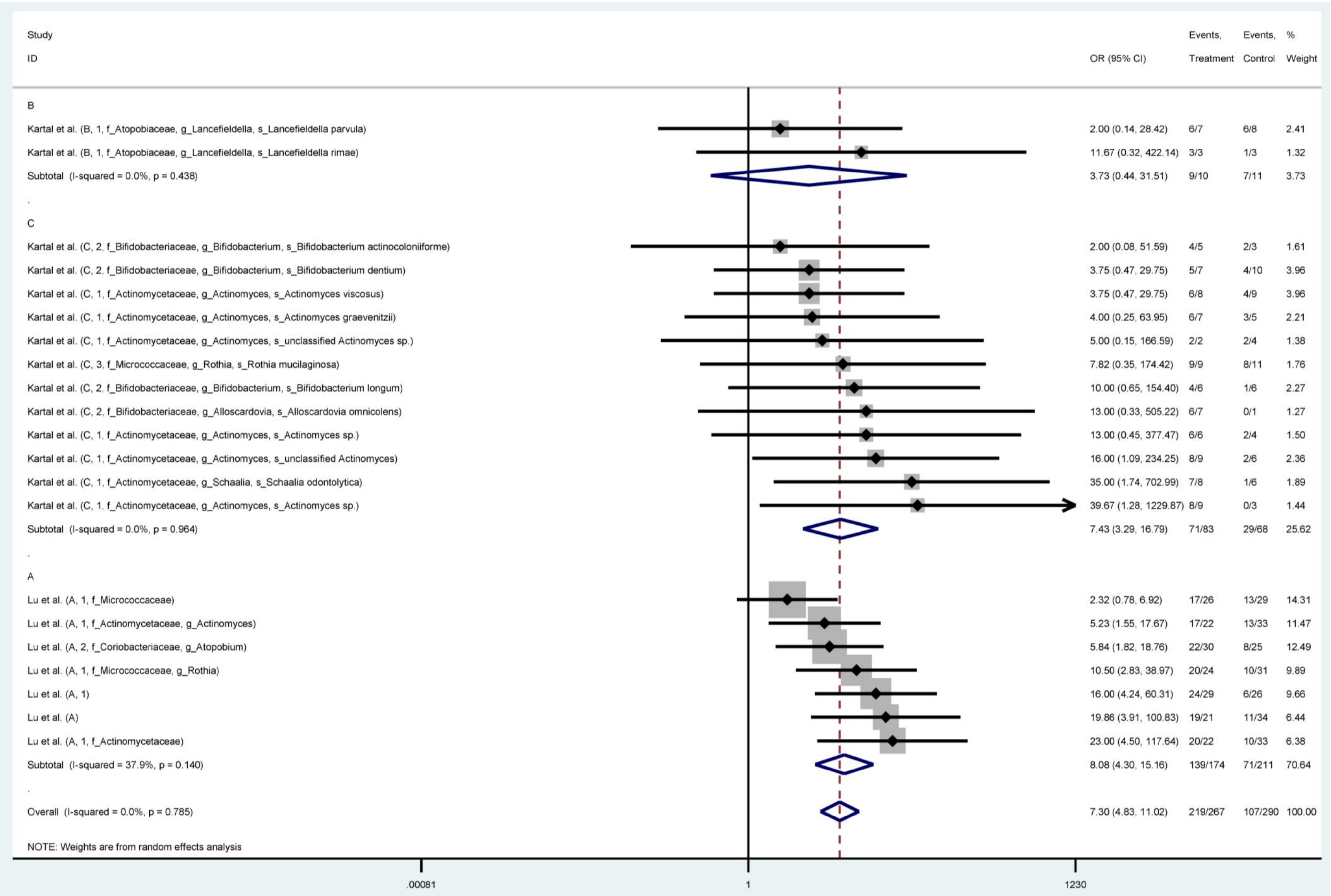

Fig S8: Forest plot meta-analysis of oral microbiome (*k_Bacteria* | *p_Actinobacteria*) in the diagnosis of PC versus healthy controls.

k_Bacteria | p_Actinobacteria

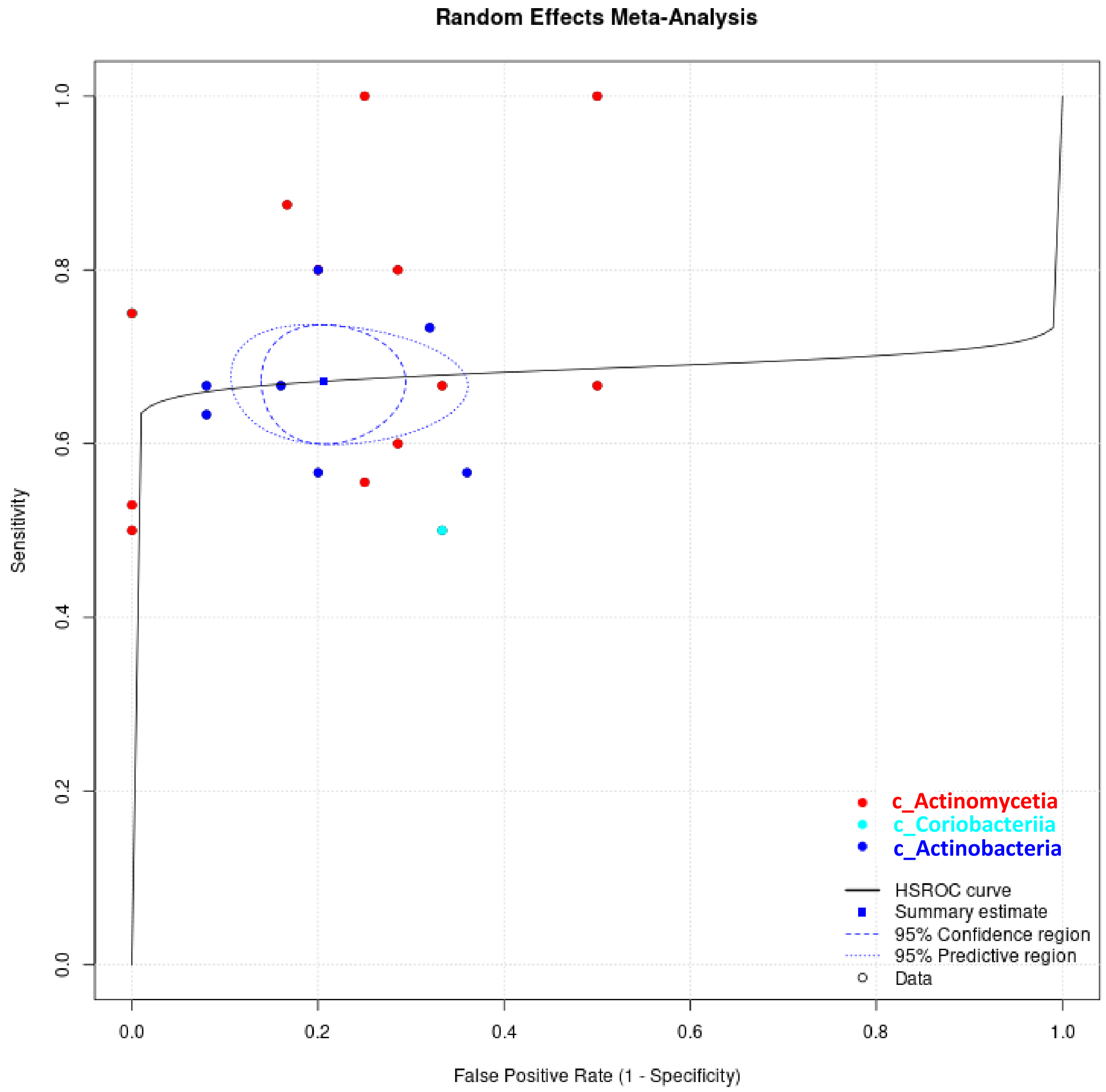

Fig S9: SROC curve of oral microbiota (class family of *k_Bacteria | p_Actinobacteria*) in the diagnosis of PC versus healthy controls using various colors for each study.

k_Bacteria | p_Actinobacteria

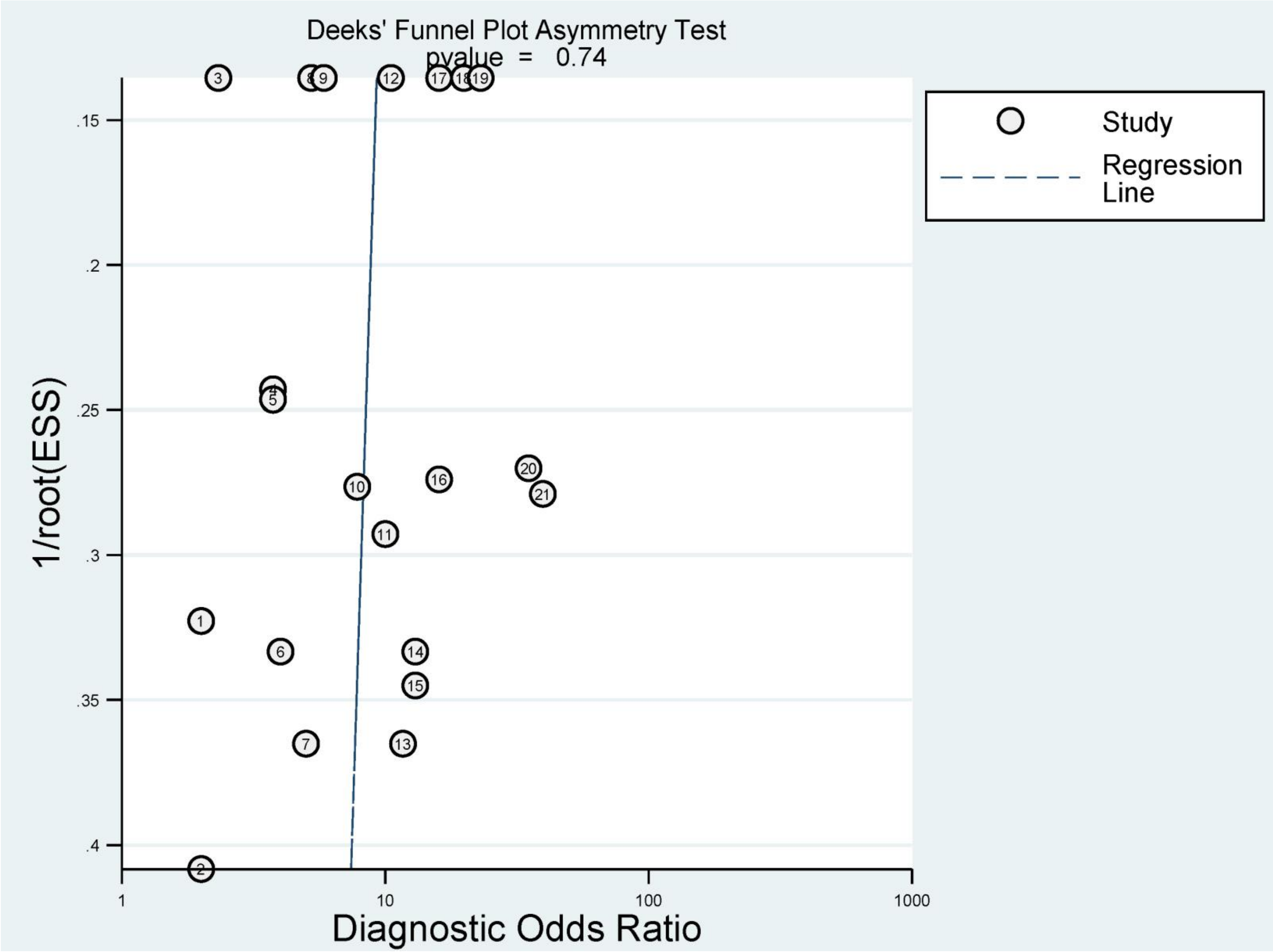

Fig S10: Deeks' funnel plot asymmetry test for publication bias in the meta-analysis of PC diagnosis using oral microbiome (*k_Bacteria | p_Actinobacteria*).

k_Bacteria | p_Firmicutes (Bacillota)

A : c_Bacilli

(1 : o_Bacillales | f_Gemella)

(2 : o_Lactobacillales | f_Aerococcaceae)

(3 : o_Lactobacillales | f_Carnobacteriaceae)

(4 : o_Lactobacillales | f_Lactobacillaceae)

(5 : o_Lactobacillales | f_Streptococcaceae)

B : c_Clostridia

(1 : o_Clostridiales)

(2 : o_Eubacteriales)

(3 : o_Erysipelotrichales)

C : c_Erysipelotrichia

(1 : o_Erysipelotrichales)

D : c_Negativicutes

(1 : o_Clostridiales)

(2 : o_Selenomonadales)

E : c_Tissierellia | o_Tissierellales | f_Peptoniphilaceae | g_Parvimonas | s_Parvimonas micra

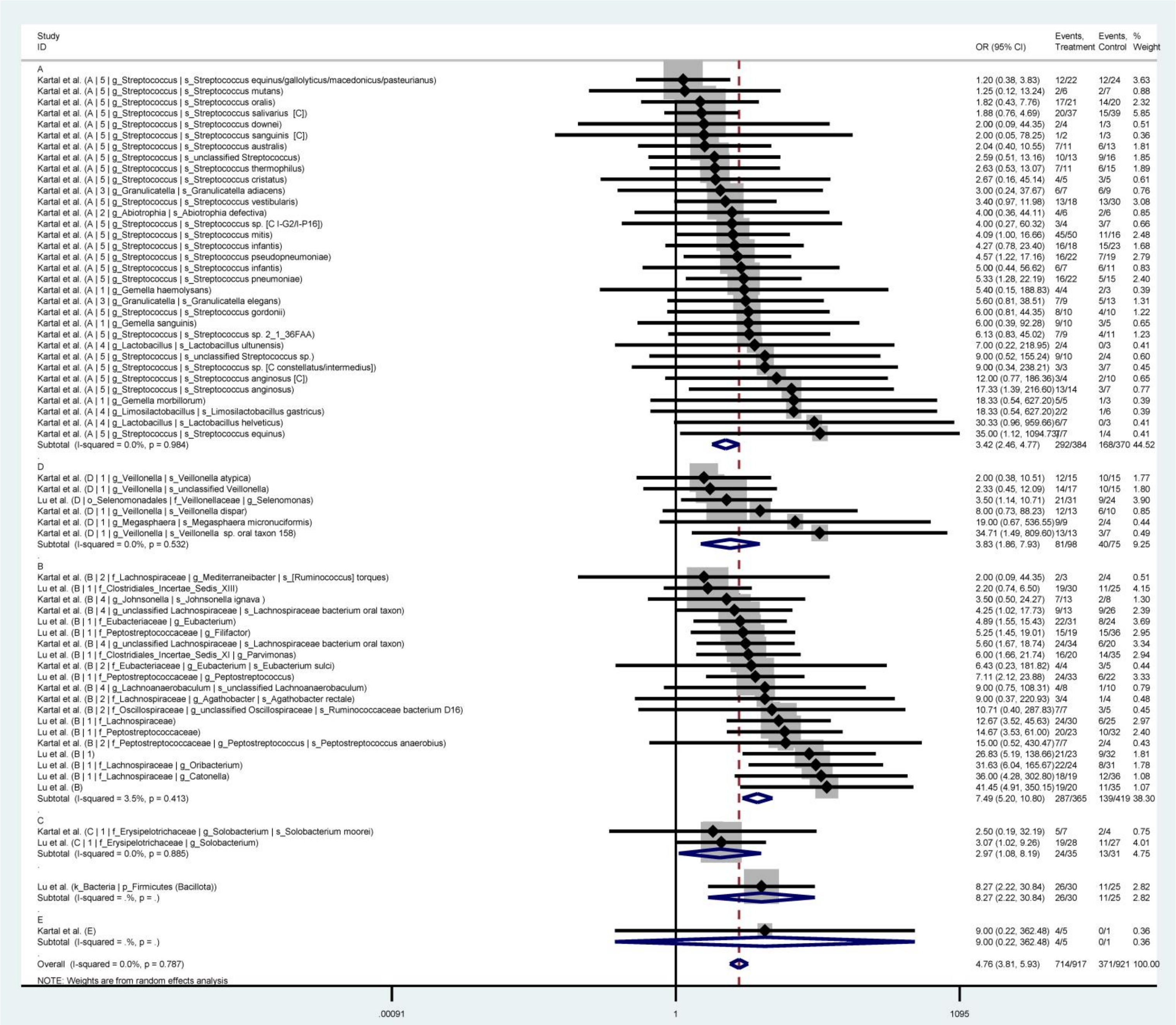

Fig S11: Forest plot of subgroup meta-analysis of oral microbiome (*k_Bacteria* | *p_Firmicutes* (*Bacillota*)) in the diagnosis of PC.

k_Bacteria | p_Firmicutes (Bacillota)

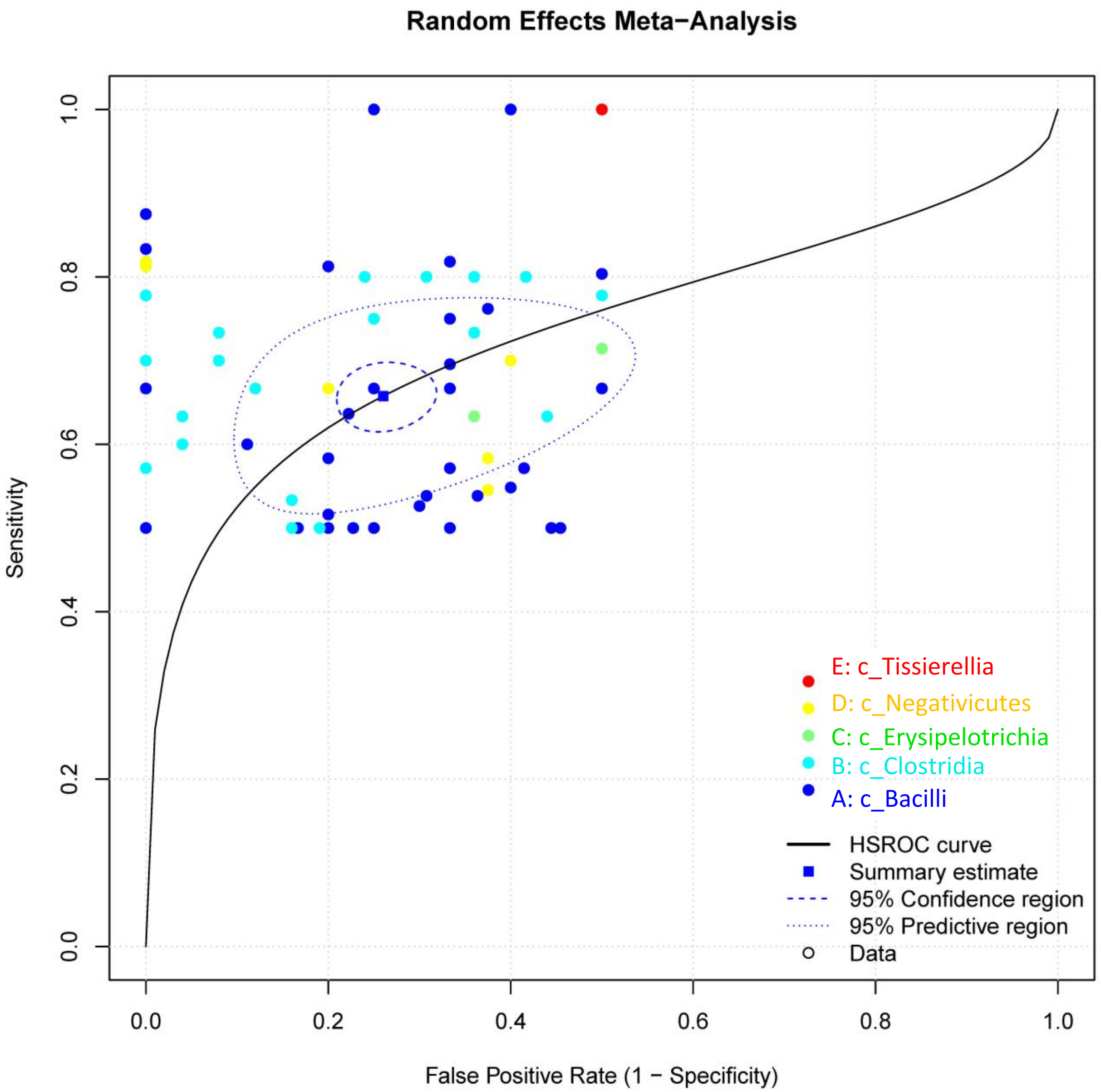

Fig S12: SROC curve of oral microbiota (class family of *k_Bacteria* | *p_Firmicutes* (*Bacillota*)) in diagnosis of PC versus healthy controls.

k_Bacteria | p_Firmicutes (Bacillota)

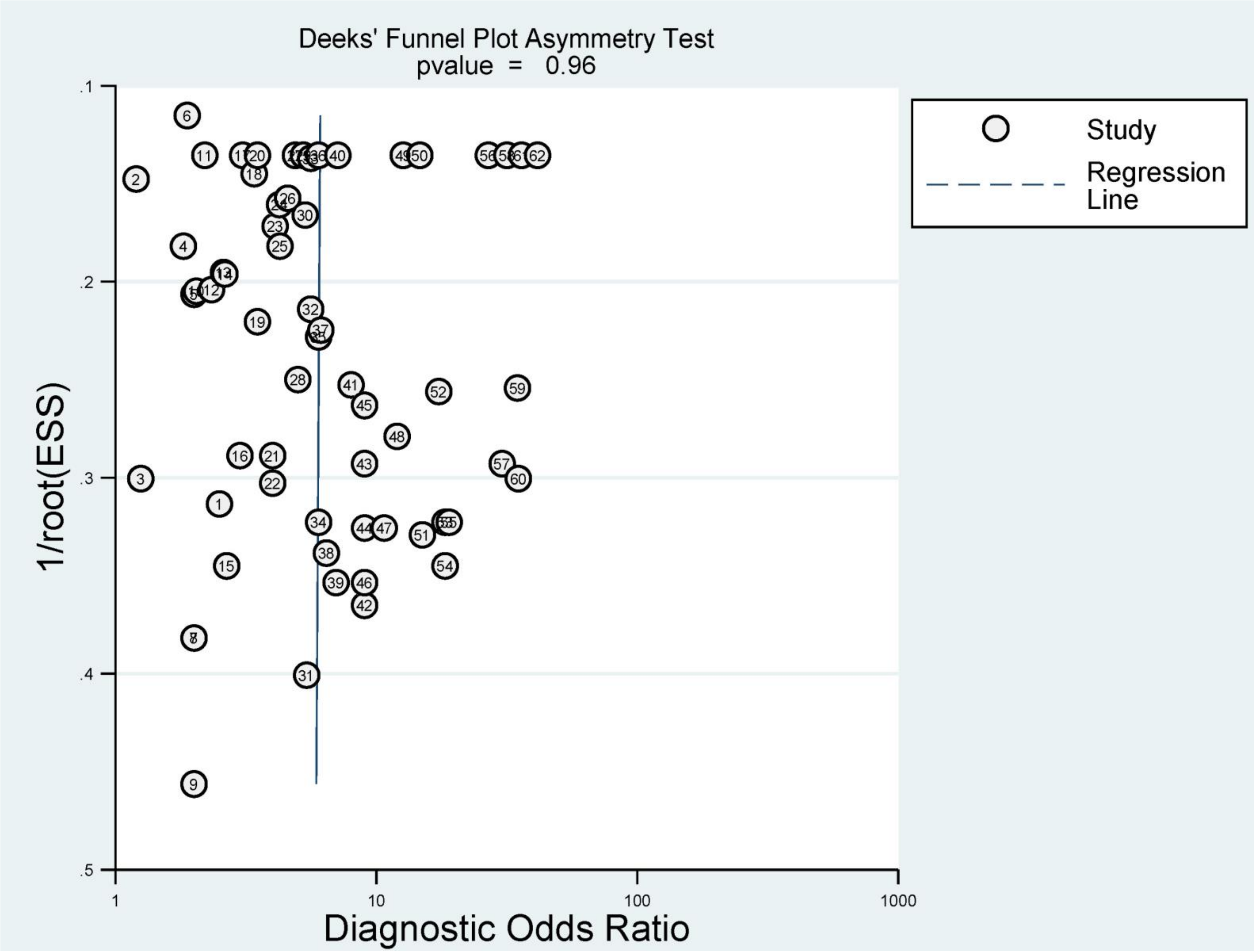

Fig S13: Deeks' funnel plot asymmetry test for publication bias in meta-analysis of PC diagnosis using oral microbiome (*k_Bacteria* | *p_Firmicutes* (*Bacillota*)).

k_Bacteria | p_Bacteroidetes

A : c_Bacteroidia | o_Bacteroidales

B : c_Bacteroidia | o_Bacteroidales | f_Prevotellaceae

(1 : g_Paraprevotella)

(2 : g_Prevotella)

C : c_Bacteroidia | o_Bacteroidales | f_Porphyromonadaceae

D : c_Bacteroidia | o_Bacteroidales | f_Rikenellaceae

(1 : g_Alistipes)

E : c_Bacteroidia | o_Bacteroidales | f_Bacteroidaceae

(1 : g_Bacteroides)

F : c_Bacteroidia | o_Bacteroidales | f_Tannerellaceae

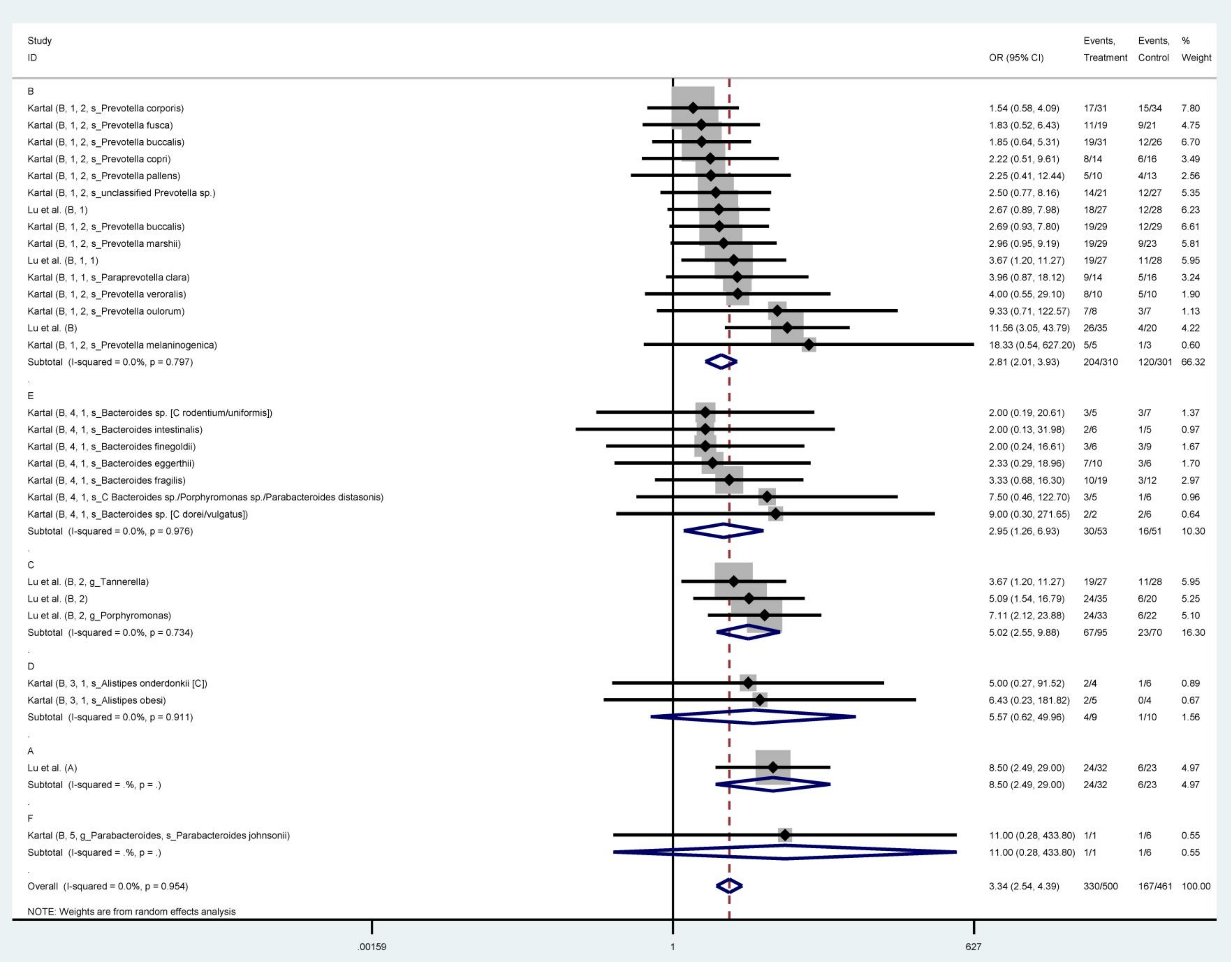

Fig S14: Forest plot of subgroup meta-analysis of oral microbiome (*k_Bacteria* | *p_Bacteroidetes*) in the diagnosis of PC.

k_Bacteria | p_Bacteroidetes

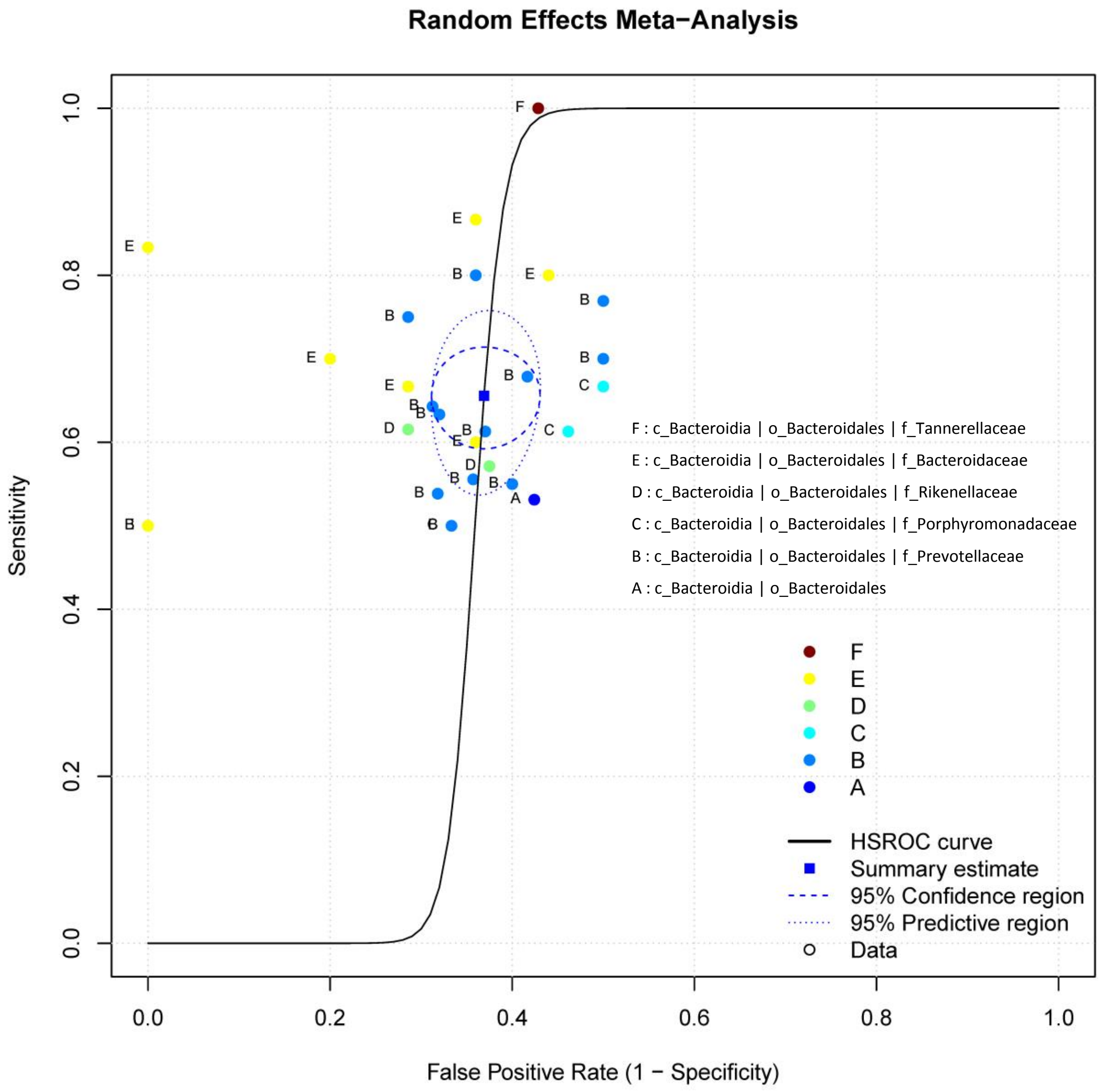

Fig S15: SROC curve of oral microbiota (class family of *k_Bacteria | p_Bacteroidetes*) in diagnosis of PC versus healthy controls.

k_Bacteria | p_Bacteroidetes

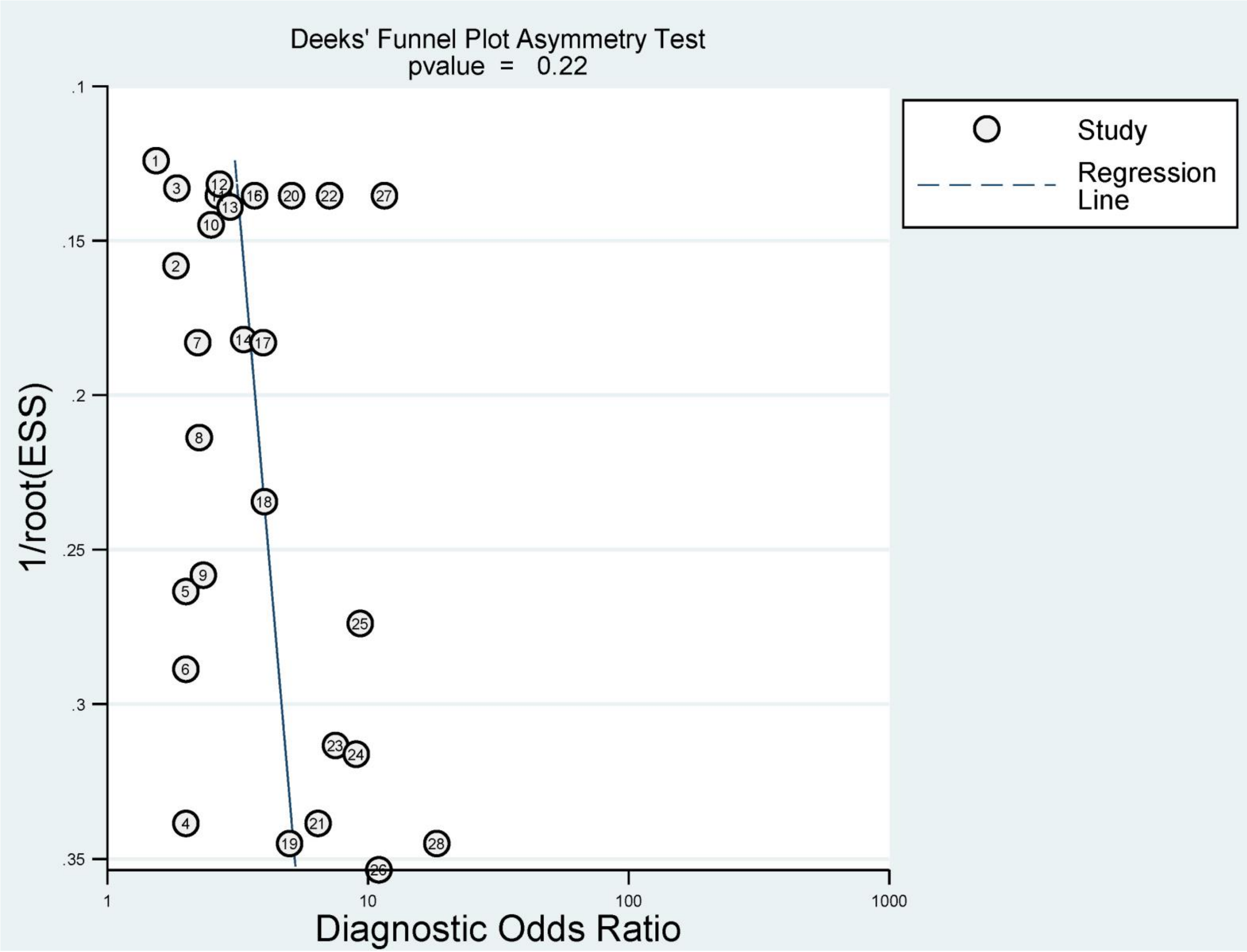

Fig S16: Deeks' funnel plot asymmetry test for publication bias in the meta-analysis of PC diagnosis using oral microbiome (class family of *k_Bacteria* | *p_Bacteroidetes*).

k_Bacteria | p_Fusobacteria

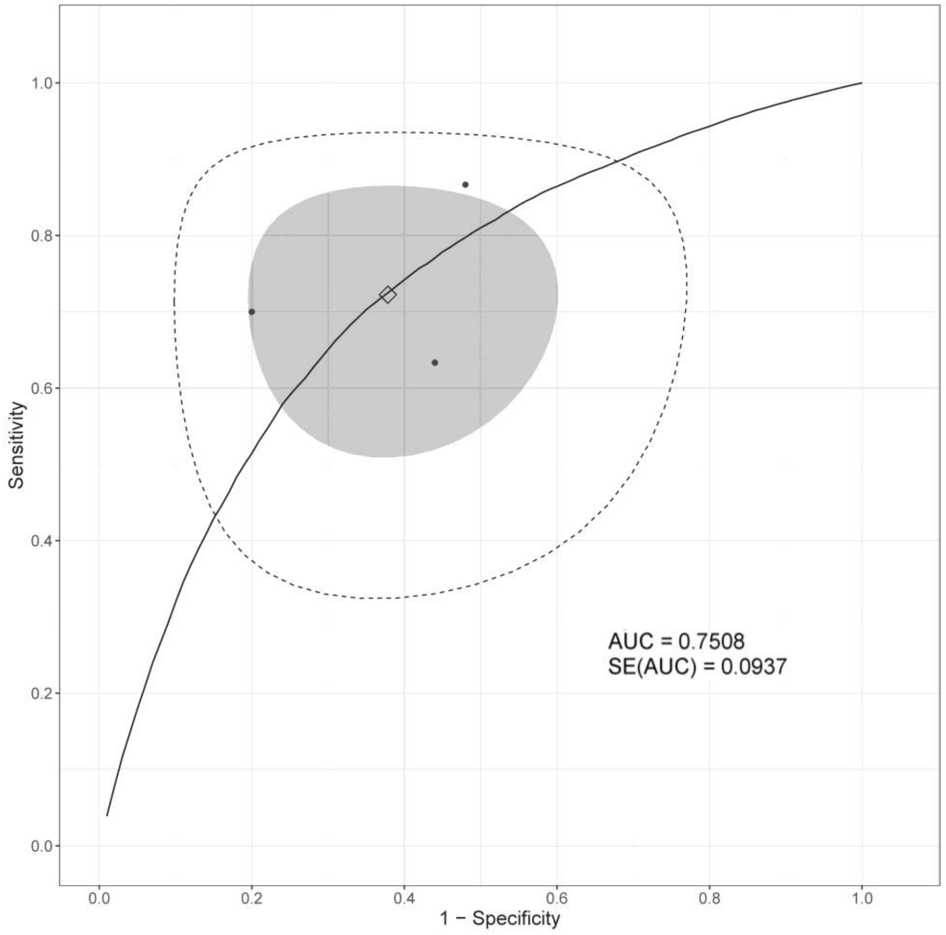

Fig S17: SROC curve of oral microbiota (class family of *k_Bacteria* | *p_Fusobacteria*) in diagnosis of PC versus healthy controls.

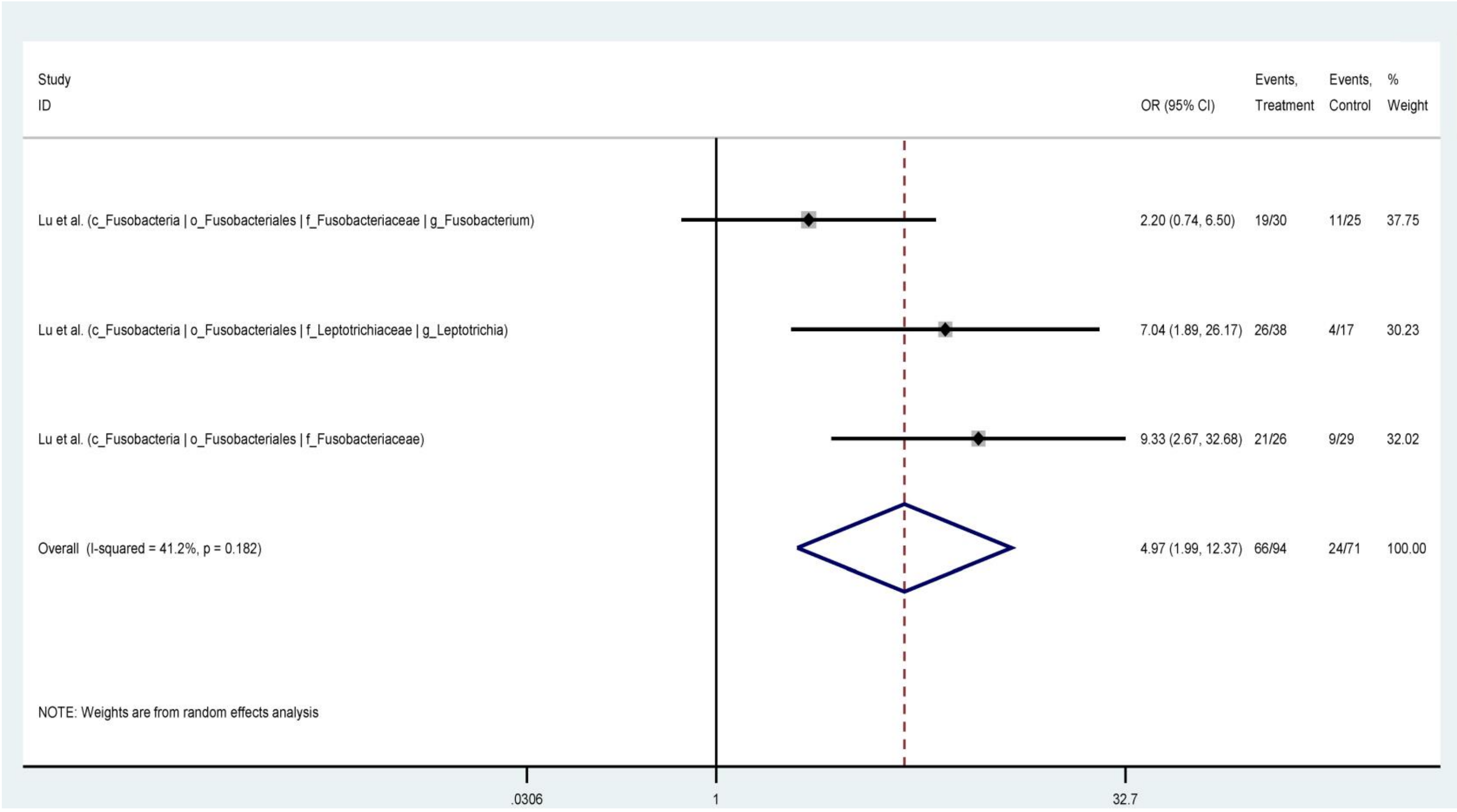

Fig S18: Forest plot of meta-analysis of oral microbiome (*k_Bacteria* | *p_Fusobacteria*) in the diagnosis of PC.

k_Bacteria | p_Proteobacteria

A : c_Gammaproteobacteria

B : c_Epsilonproteobacteria

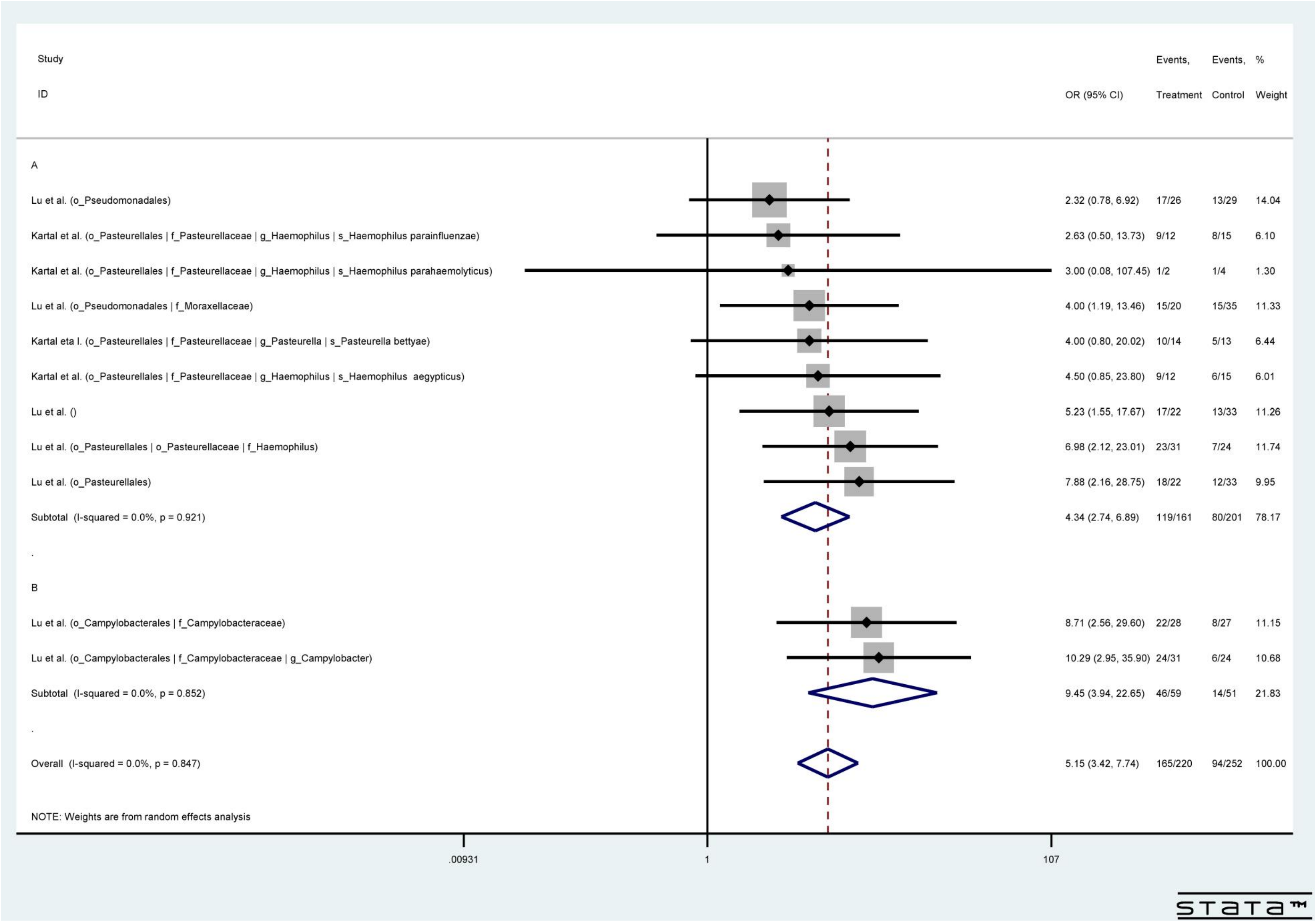

Fig S19: Forest plot of subgroup meta-analysis of oral microbiome (*k_Bacteria | p_Proteobacteria*) in the diagnosis of PC.

k_Bacteria | p_Proteobacteria

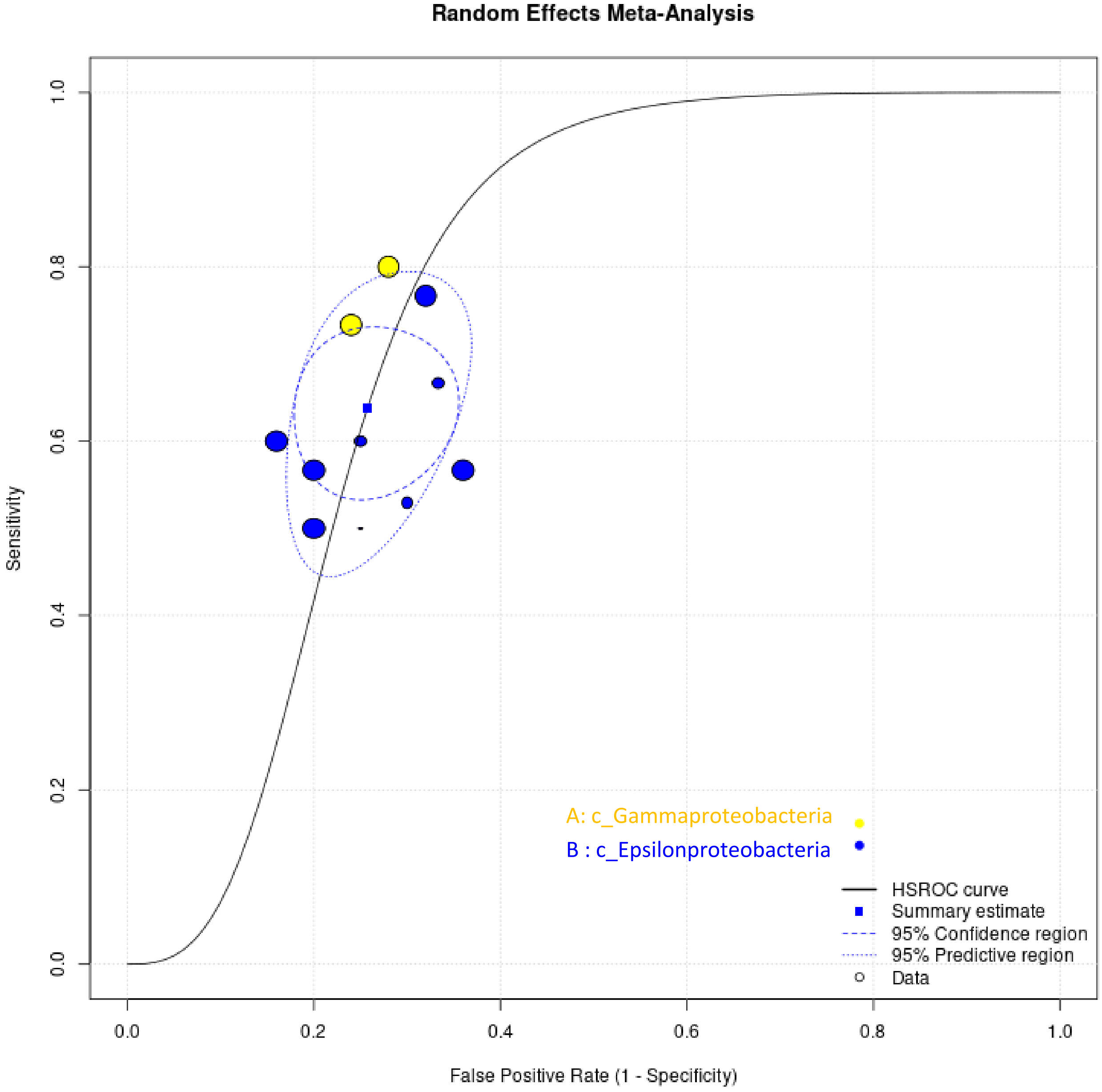

Fig S20: SROC curve of oral microbiota (class family of *k_Bacteria | p_Proteobacteria*) in diagnosis of PC versus healthy controls considering study weights of study units.

k_Bacteria | p_Proteobacteria

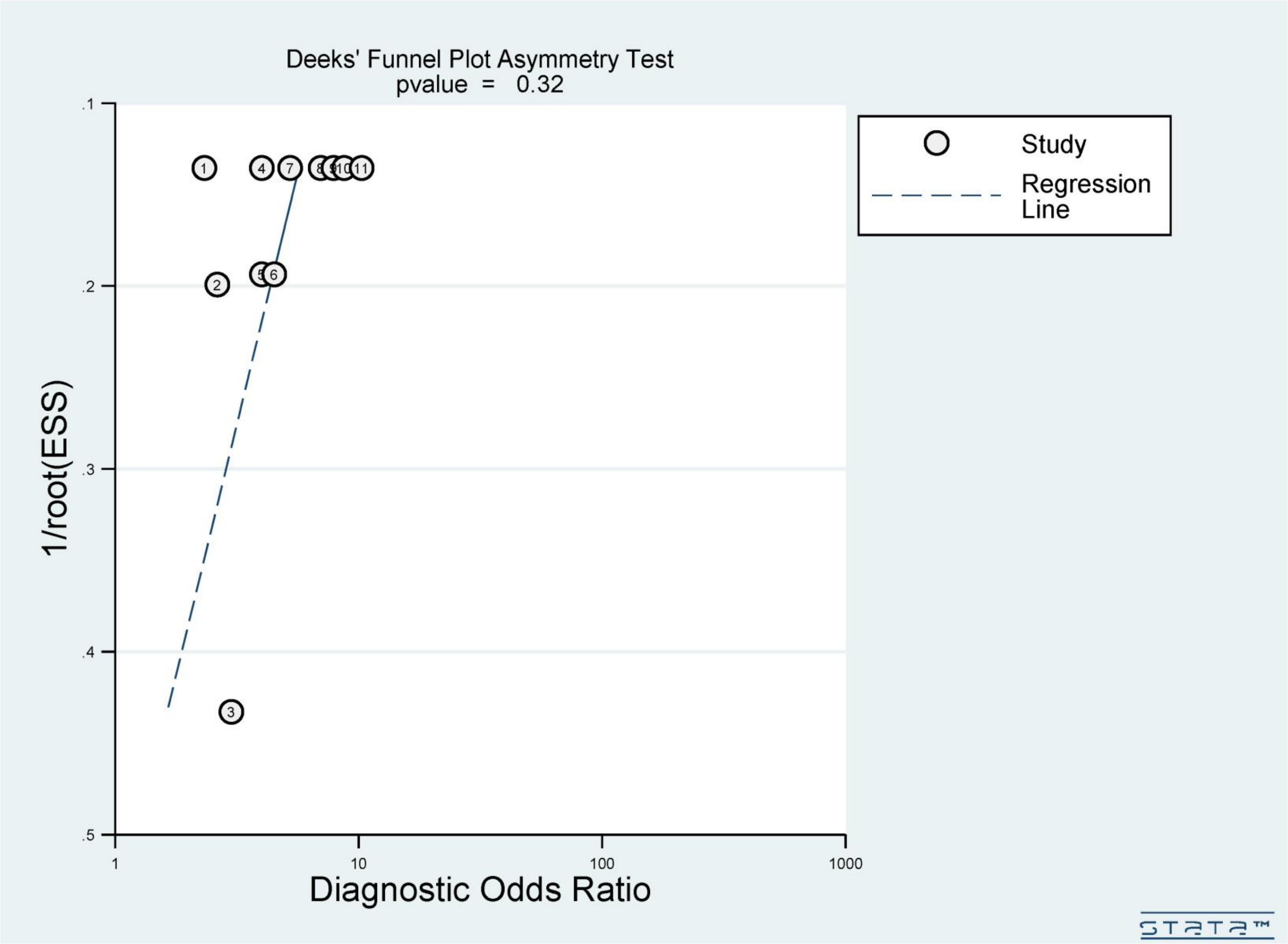

Fig S21: Deeks' funnel plot asymmetry test for publication bias in meta-analysis of PC diagnosis using oral microbiome (class family of *k_Bacteria* | *p_Proteobacteria*).

Multiple oral microbiome

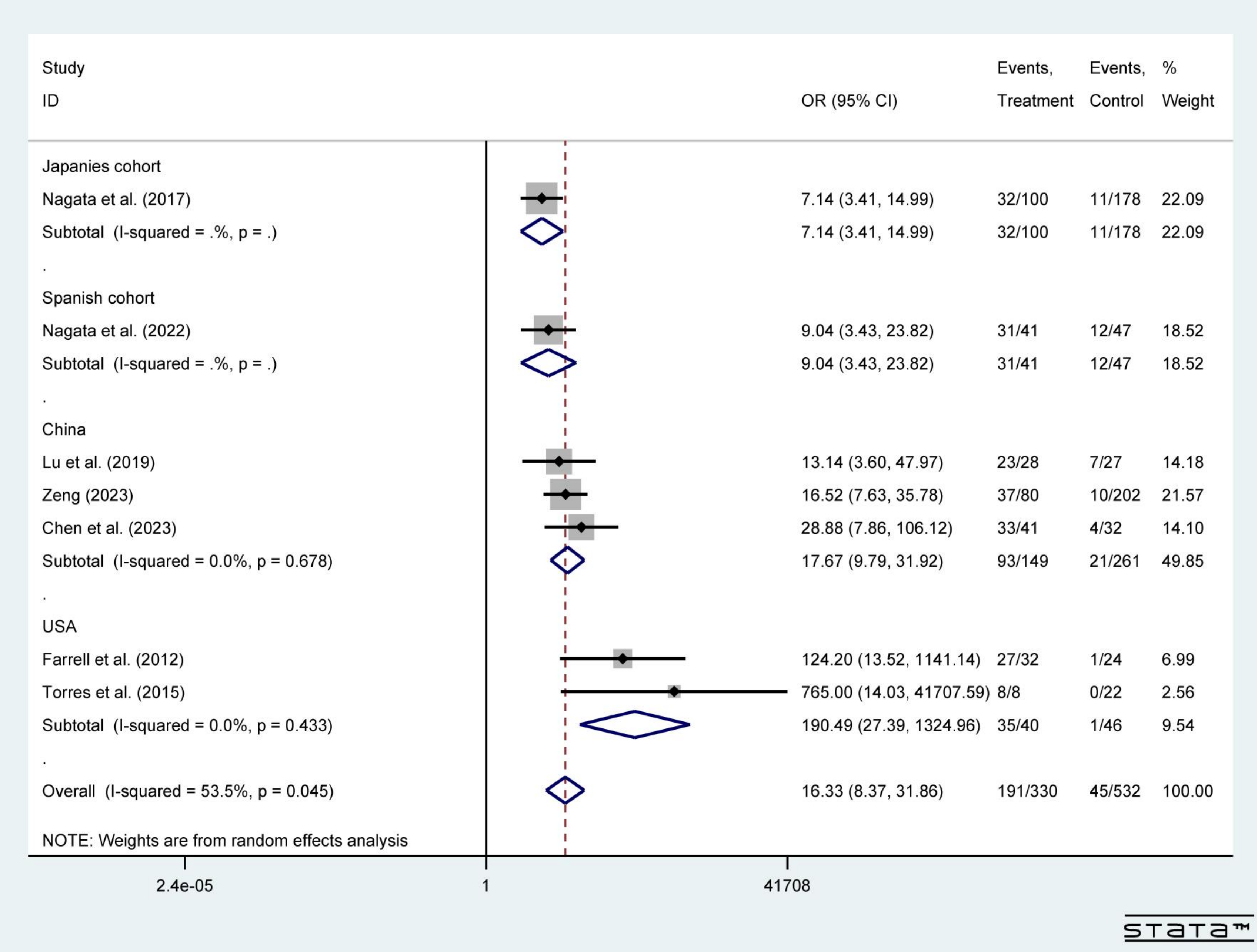

Fig S22: Forest plot of the subgroup meta-analysis of oral microbiome (multiple oral microbiome) and country in the diagnosis of PC.

Multiple oral microbiome

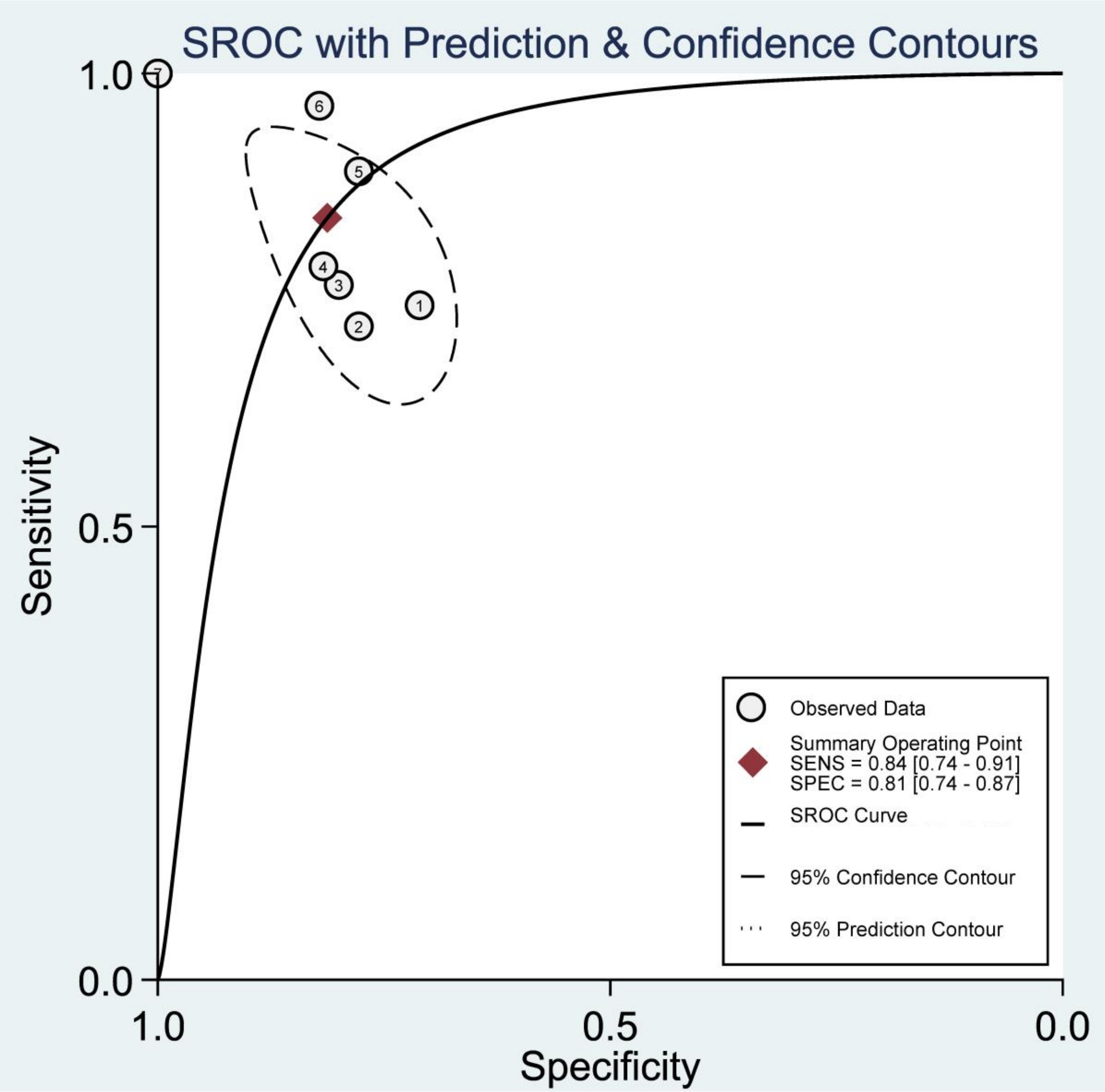

Fig S23: SROC curve of subgroup meta-analysis of multiple oral microbiome for PC diagnosis.

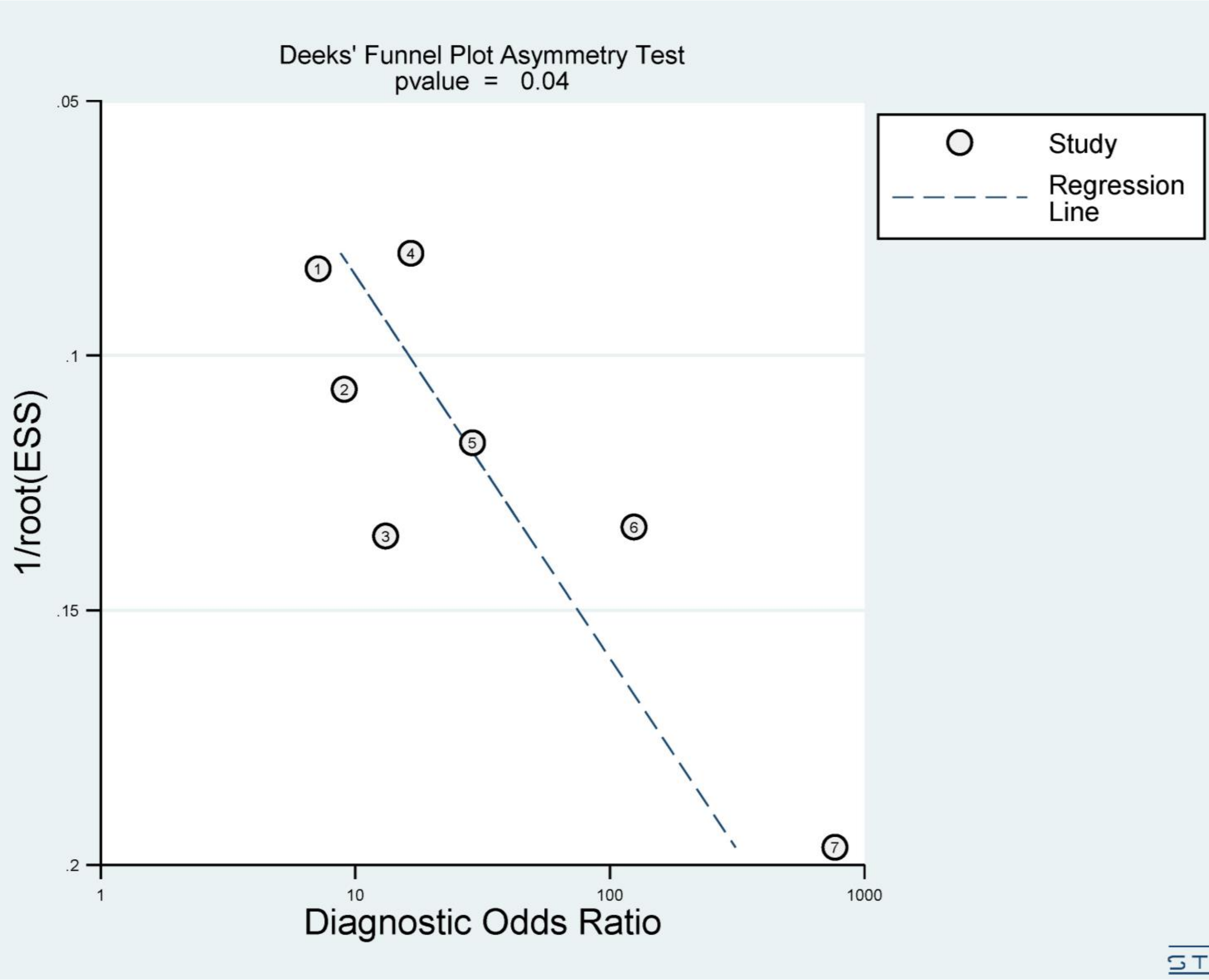

Fig S24: Deeks' funnel plot asymmetry test for publication bias in meta-analysis of PC diagnosis using multiple oral microbiome.

Oral sampling method (PC vs. healthy)

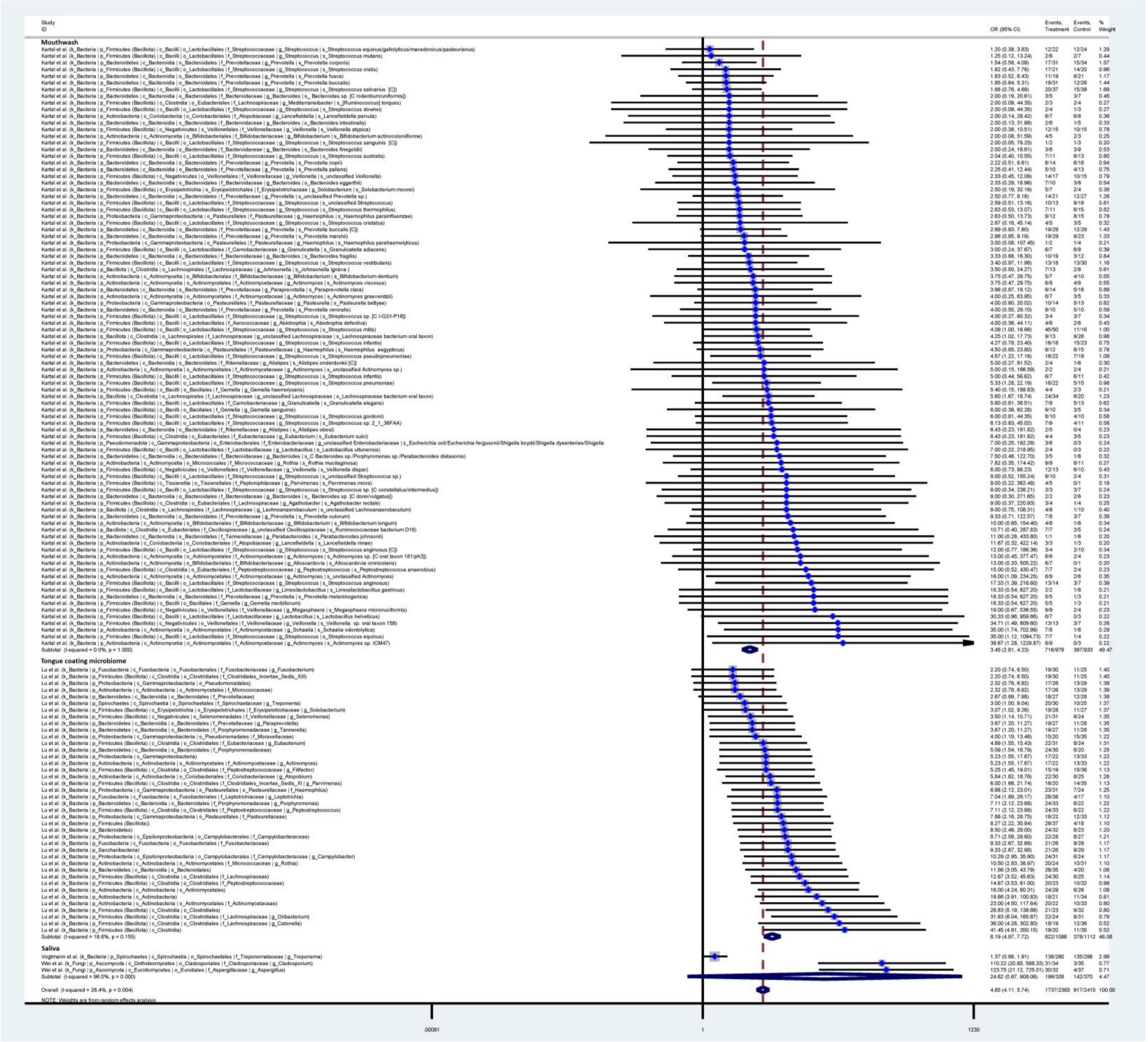

Fig S25: Forest plot of the subgroup meta-analysis of oral sampling method in the diagnosis of PC.

Oral sampling method (PC vs. healthy)

Mouthwash

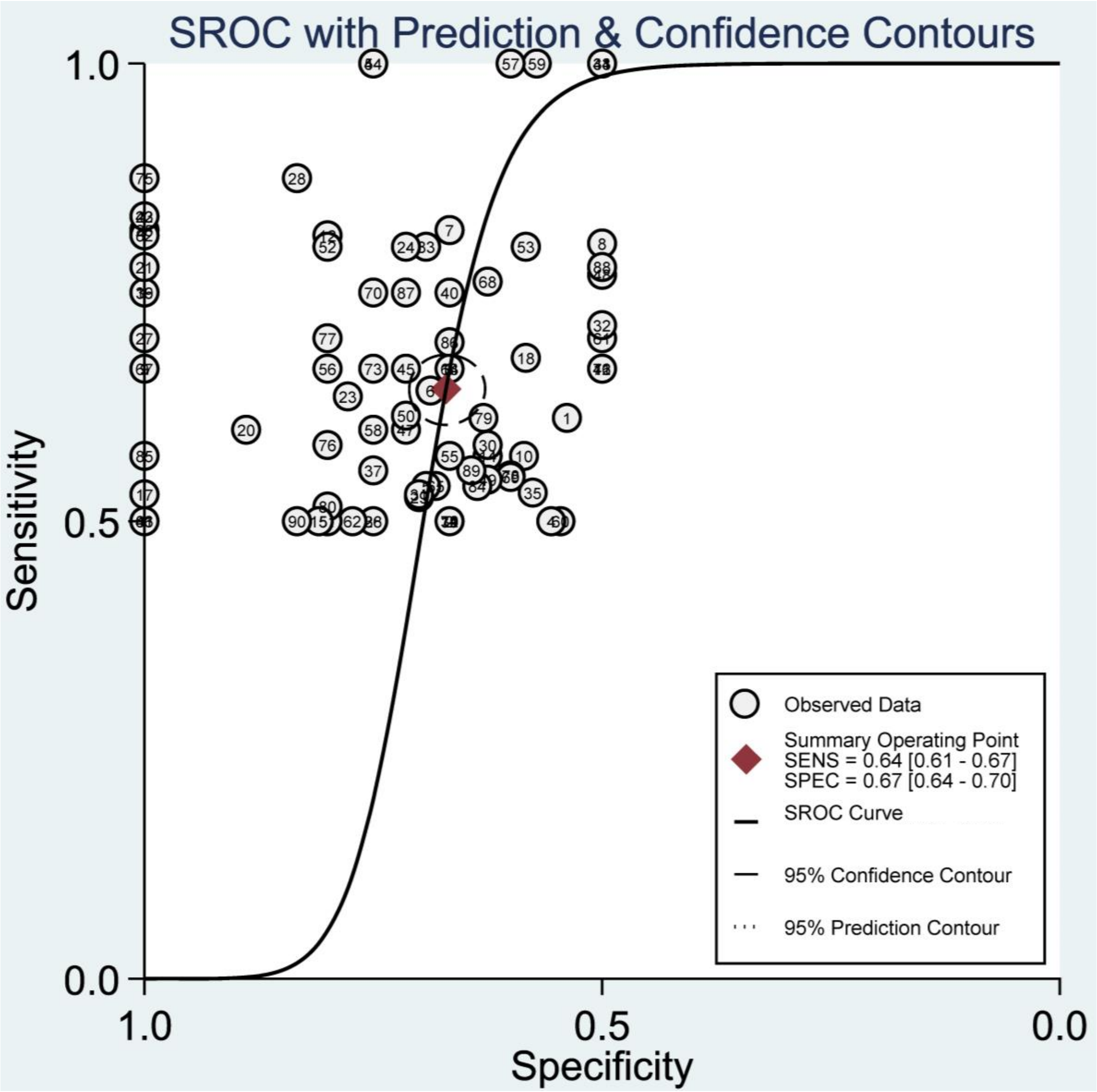

Fig S26: SROC curve of mouthwash in the diagnosis of PC.

Tongue coating

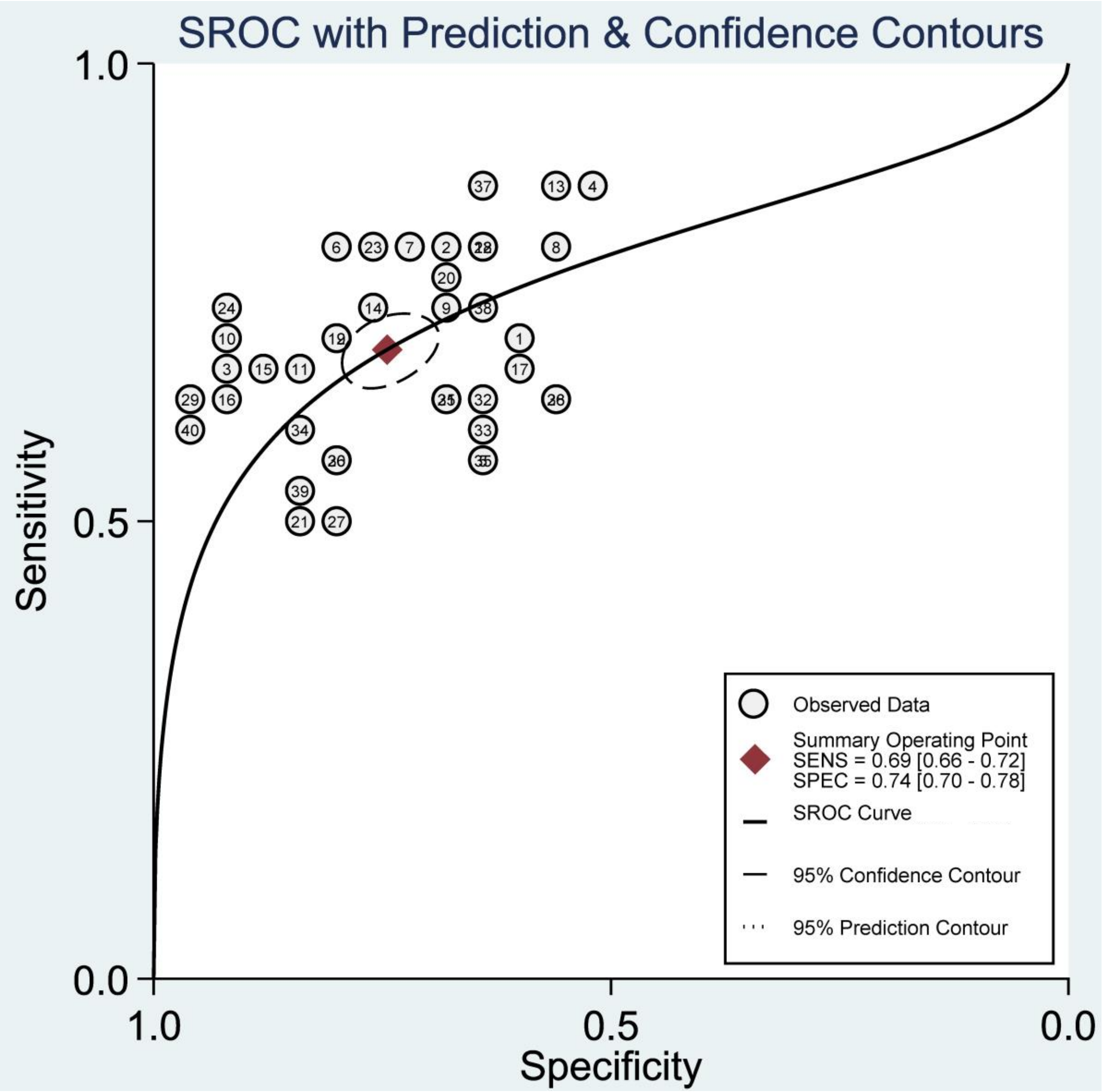

Fig S27: SROC curve of tongue coating in the diagnosis of PC.

Bacterial taxonomy

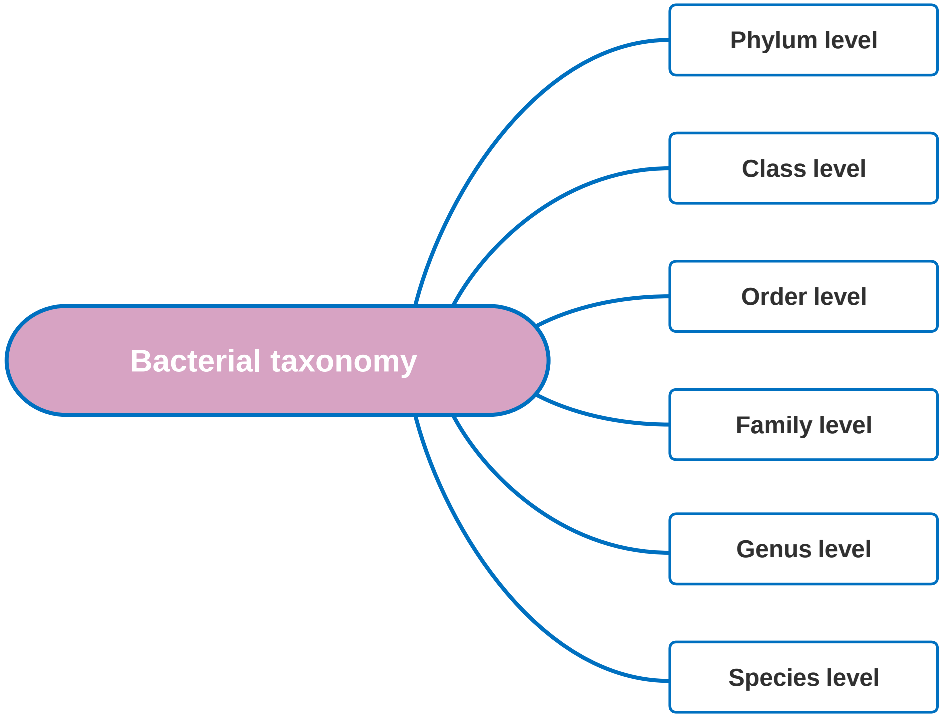

Fig S28: Bacterial taxonomy in PC diagnosis

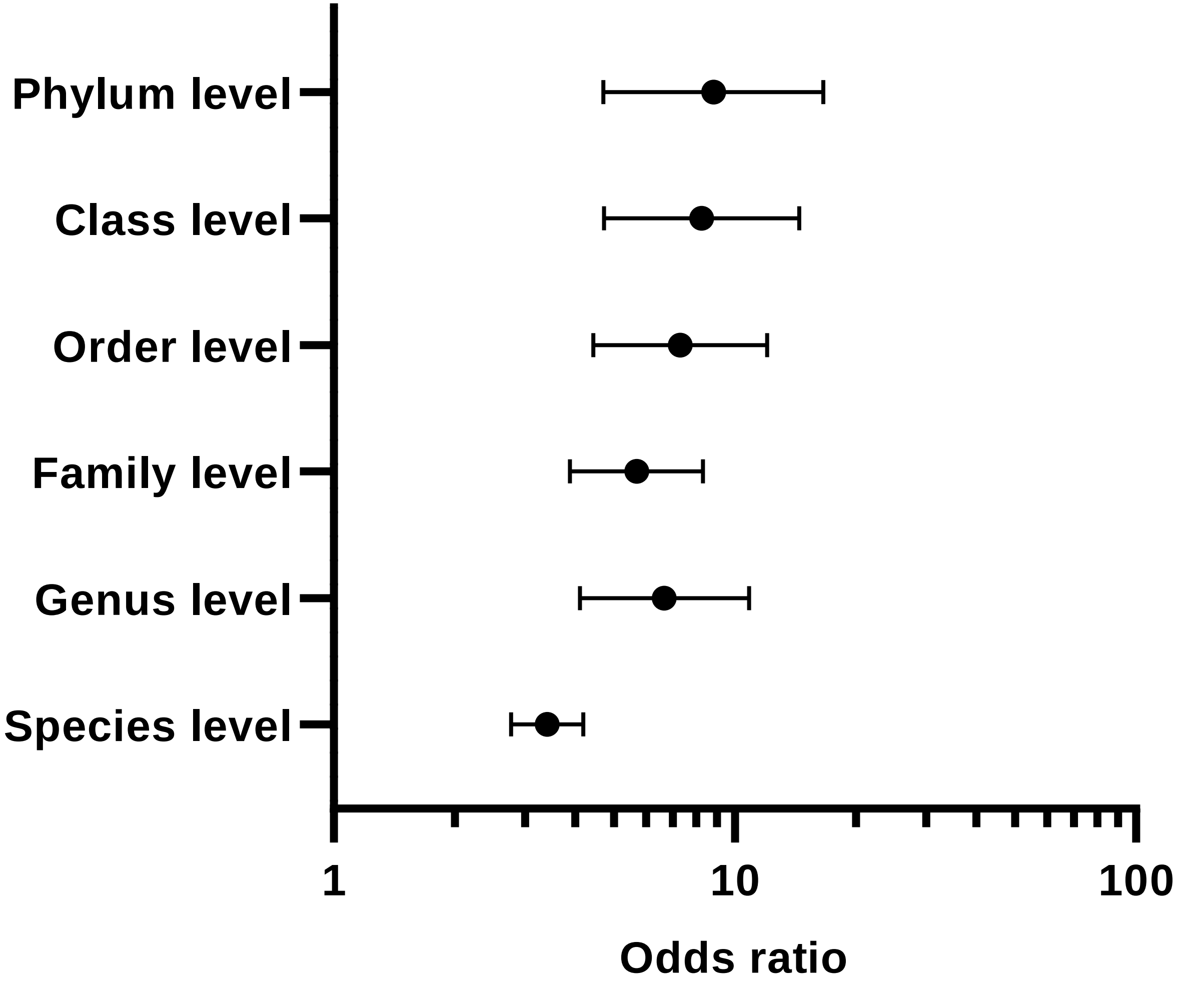

Fig S29: Diagnostic odds ratio for the subgroup meta-analysis of PC diagnosis using bacterial taxonomy.

Bacterial taxonomy, Phylum levels

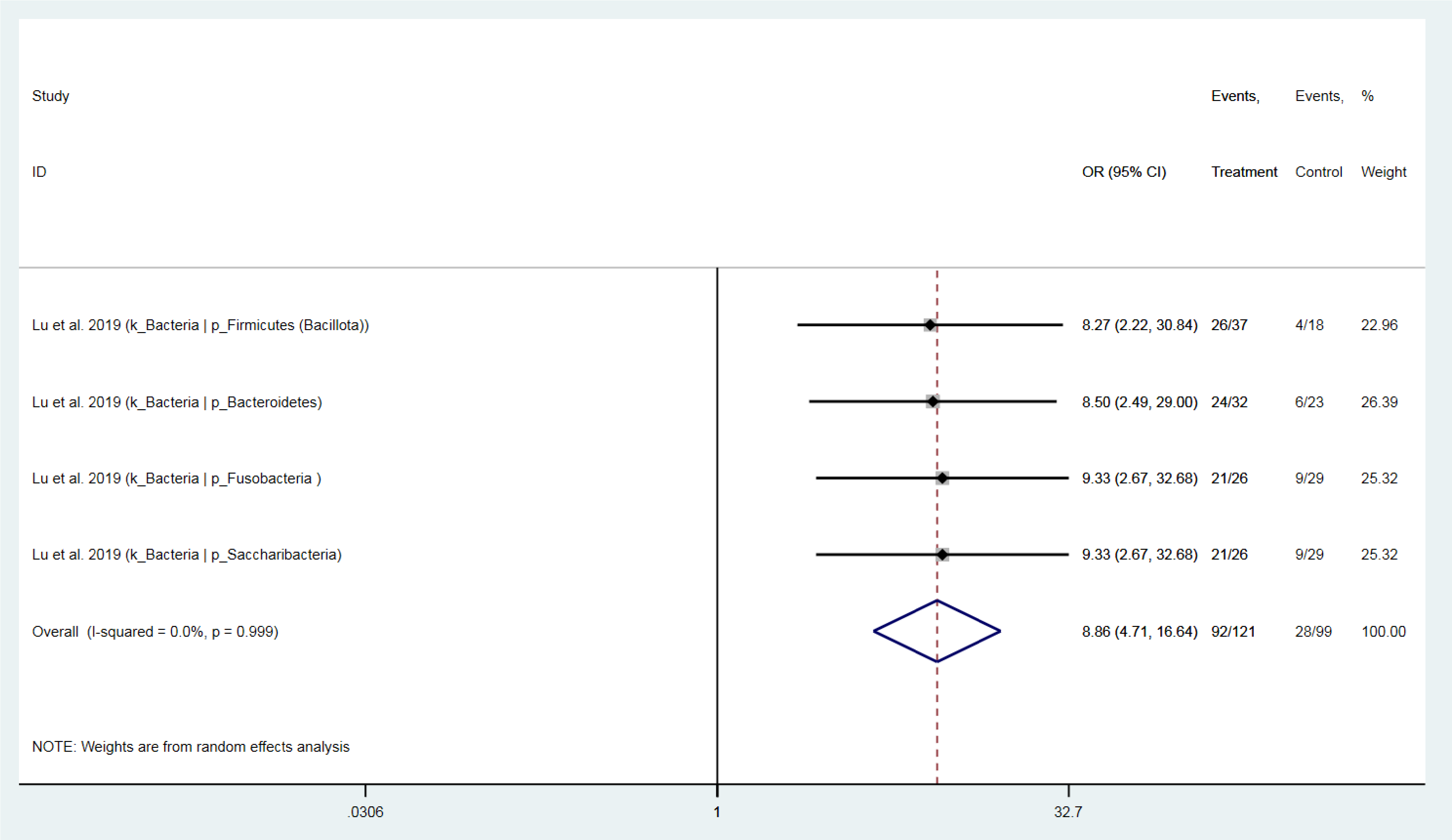

Fig S30: Forest plot of the meta-analysis of oral microbiome in the diagnosis of PC using phylum-levels.

Bacterial taxonomy, Phylum levels

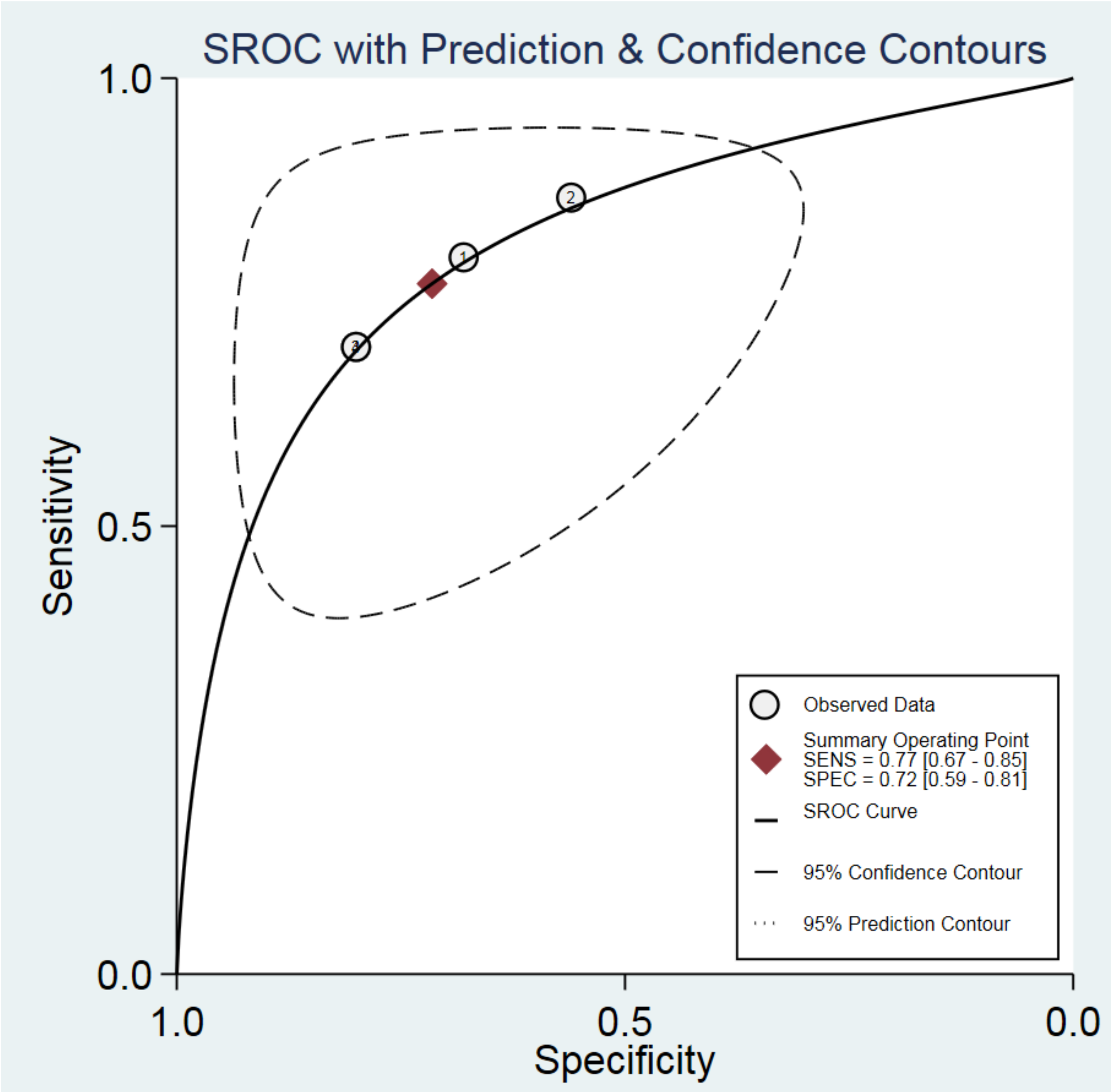

Fig S31: SROC curve of the meta-analysis of oral microbiome in the diagnosis of PC using phylum-levels.

Bacterial taxonomy, Class level

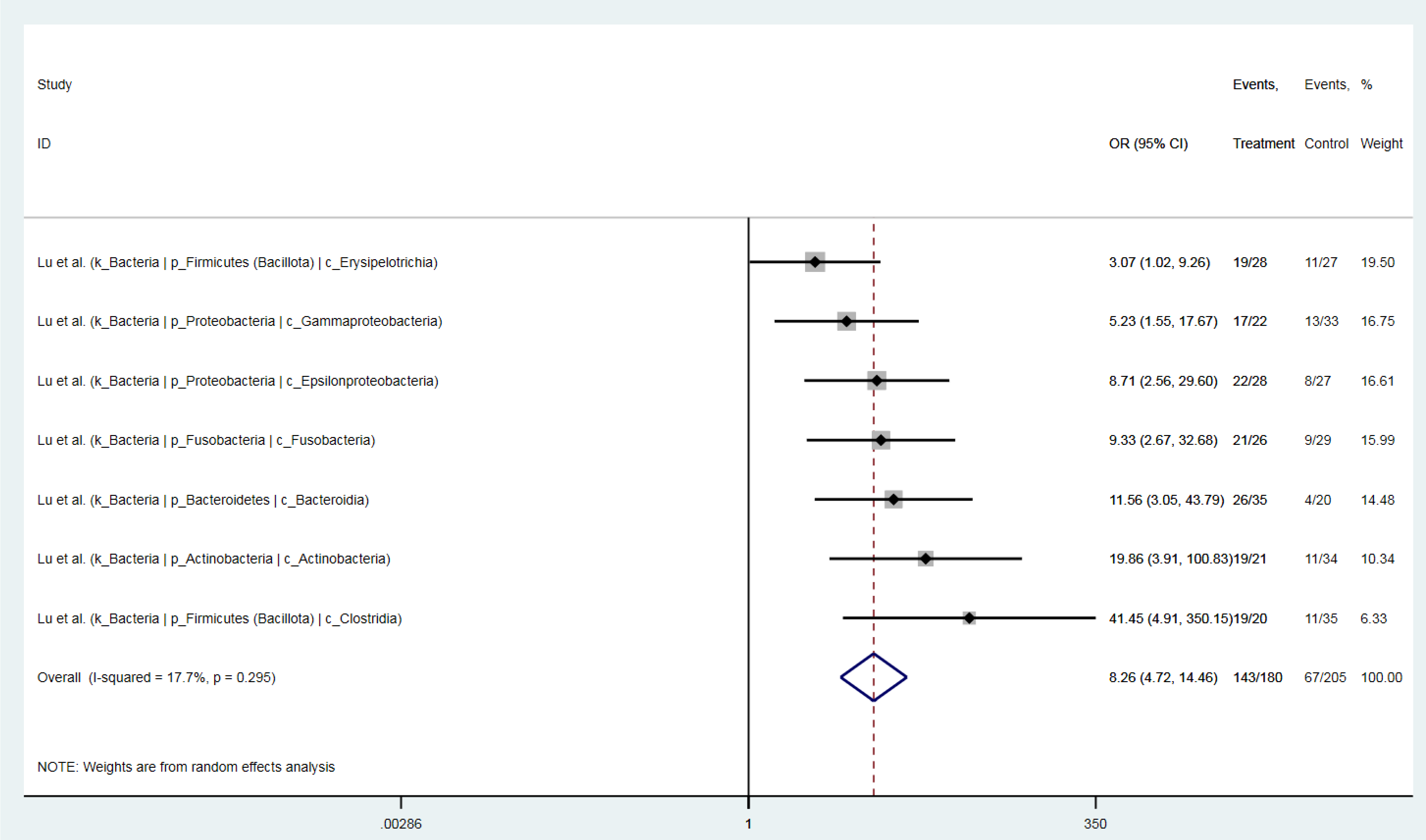

Fig S32: Forest plot of the meta-analysis of oral microbiome in the diagnosis of PC using class-levels.

Bacterial taxonomy, Class level

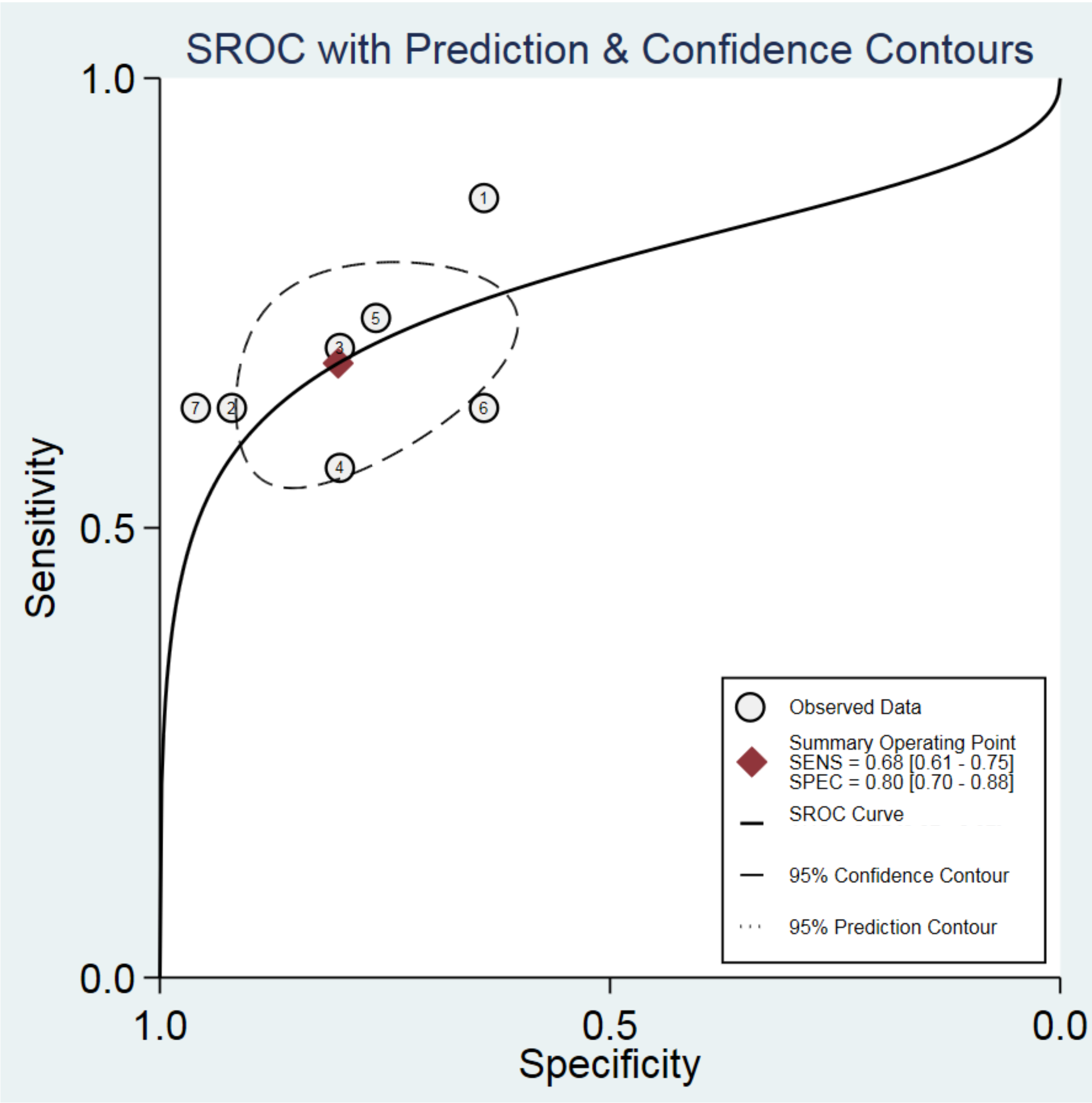

Fig S33: SROC curve of the meta-analysis of oral microbiome in the diagnosis of PC using class-levels.

Bacterial taxonomy, Order level

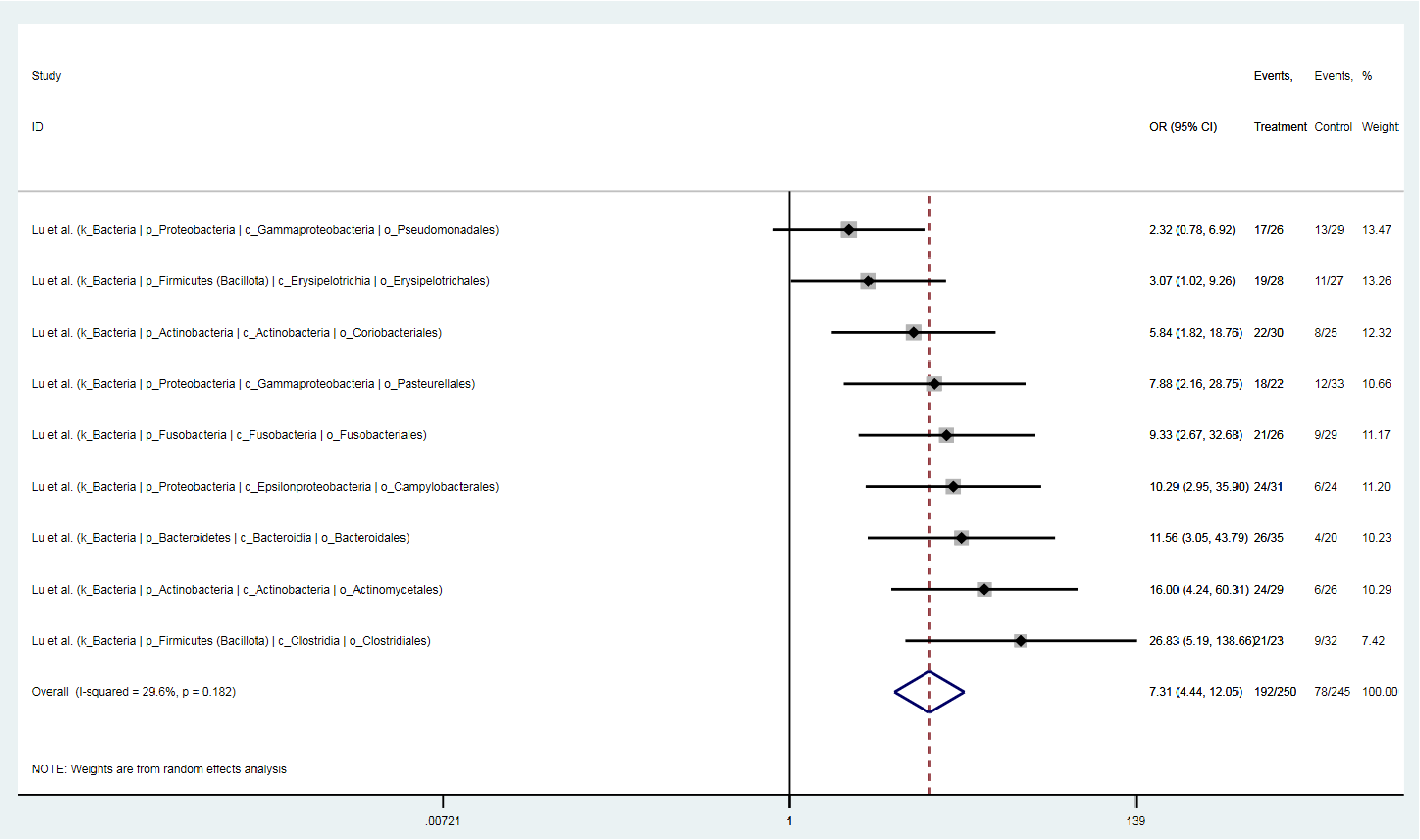

Fig S34: Forest plot of the meta-analysis of oral microbiome in the diagnosis of PC using order levels.

Bacterial taxonomy, Order level

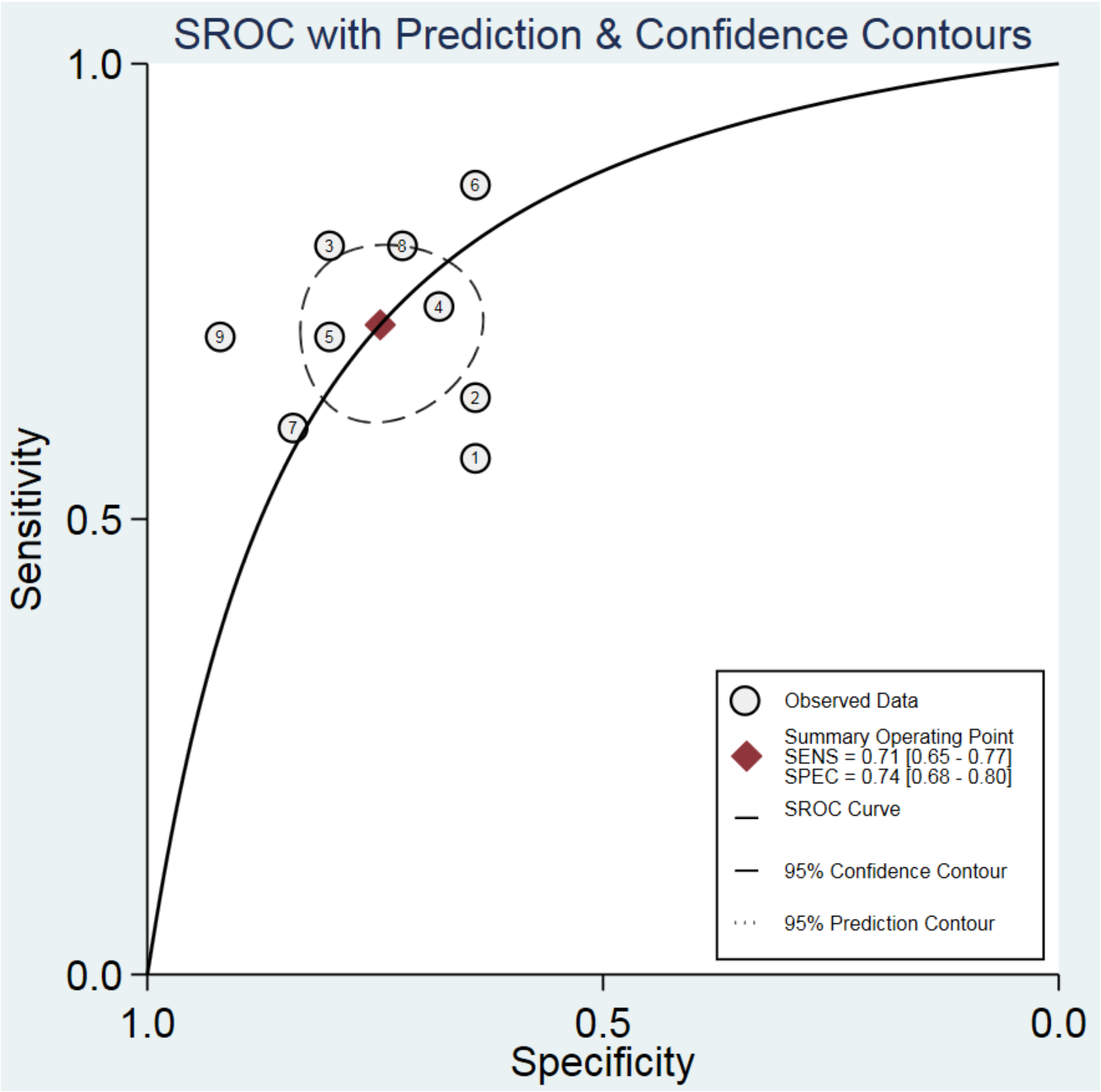

Fig S35: SROC curve of the meta-analysis of oral microbiome in the diagnosis of PC using order levels.

Bacterial taxonomy, Family level

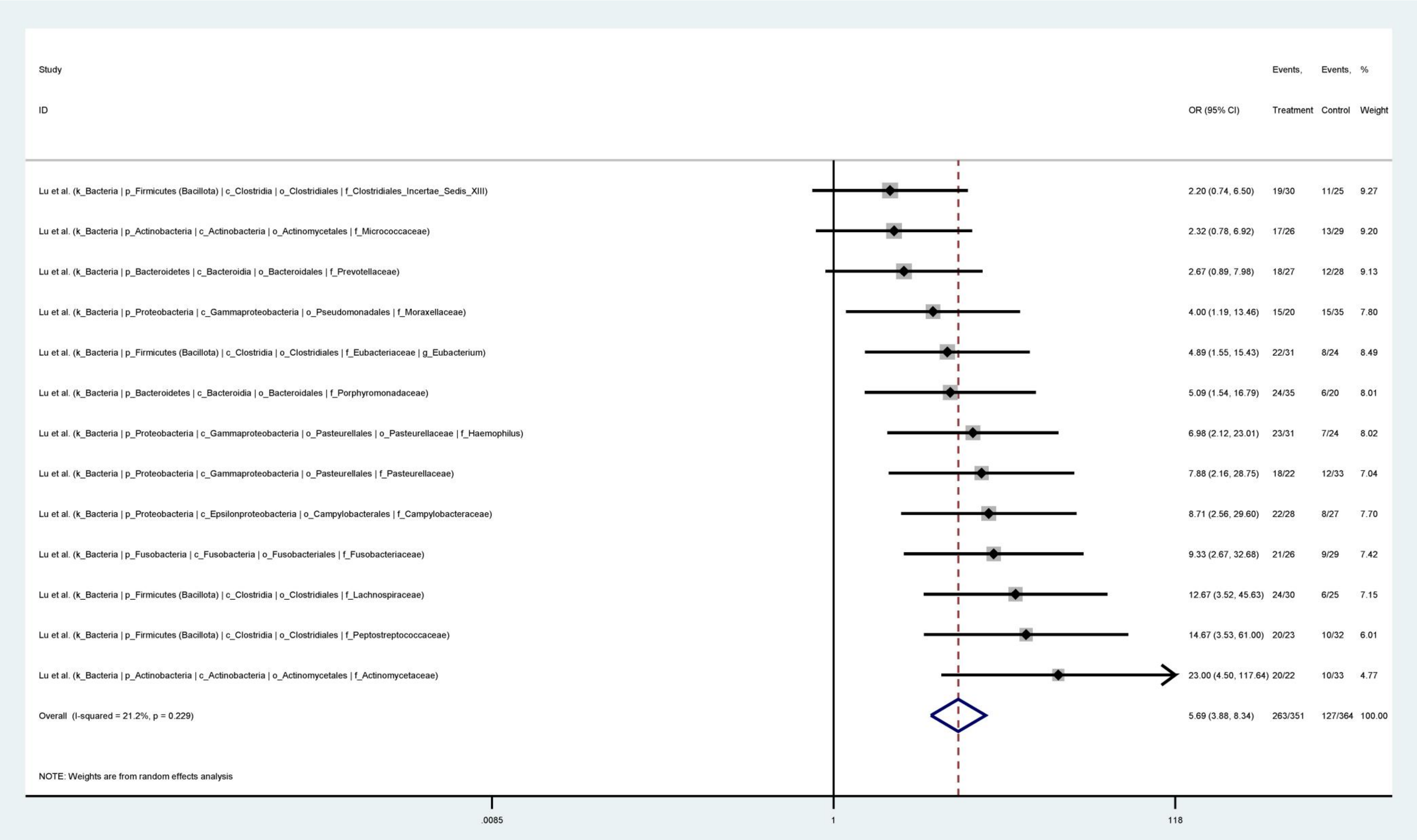

Fig S36: Forest plot of the meta-analysis of oral microbiome in the diagnosis of PC using family-levels.

Bacterial taxonomy, Family level

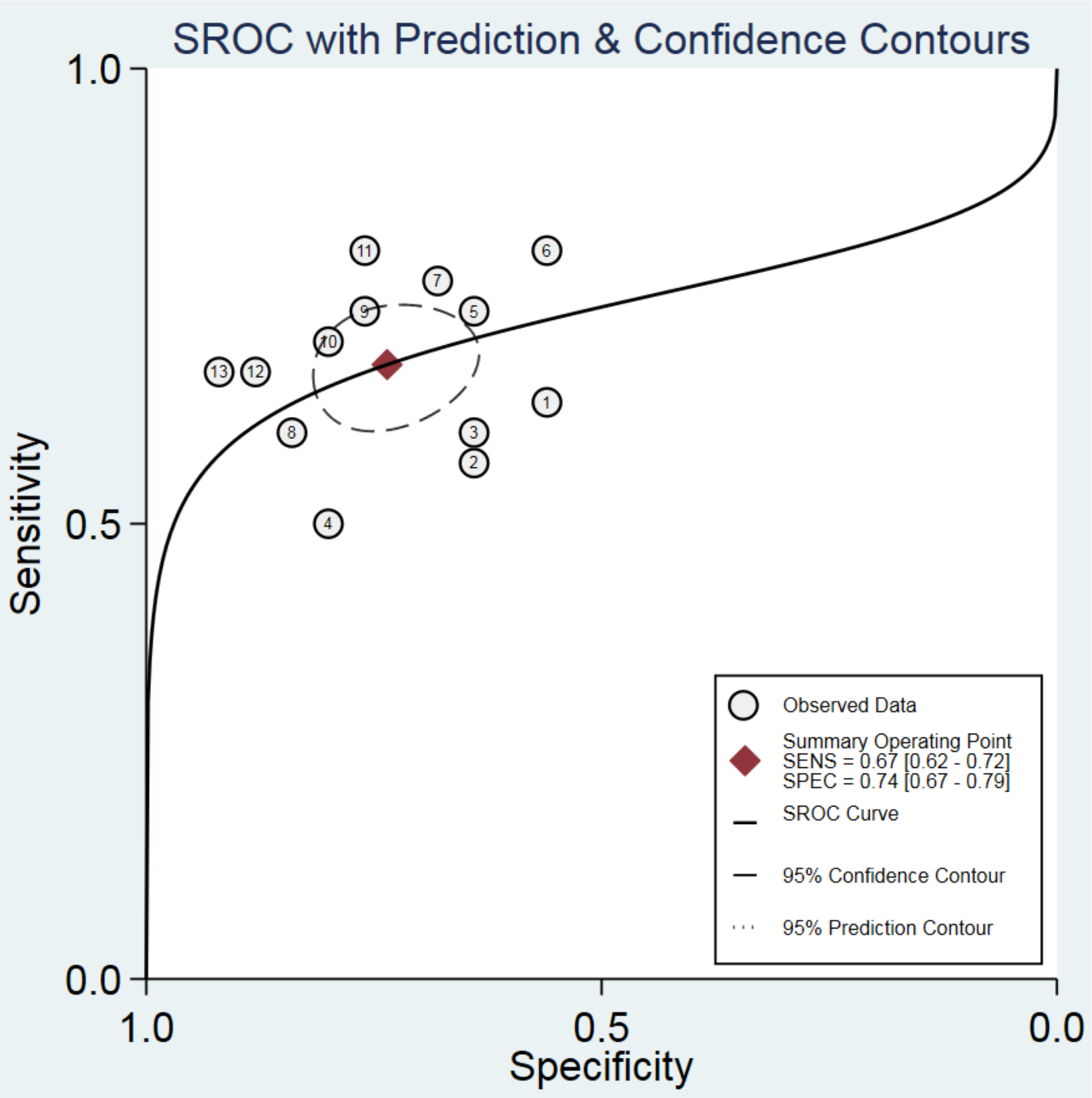

Fig S37: SROC curve of the meta-analysis of oral microbiome in the diagnosis of PC using family-levels.

Bacterial taxonomy, Genus level

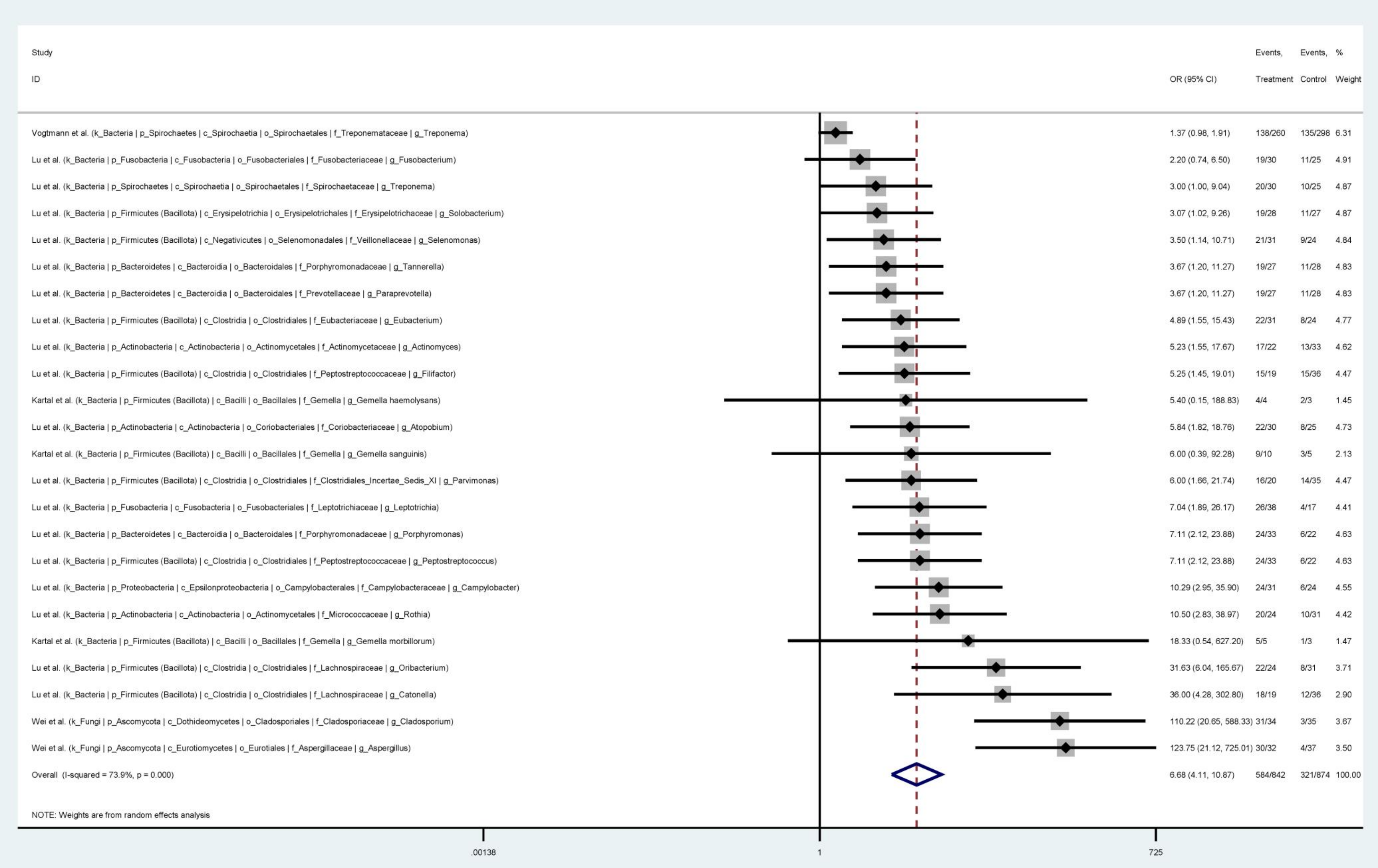

Fig S38: Forest plot of the meta-analysis of oral microbiome in the diagnosis of PC using genus levels.

Bacterial taxonomy, Genus level

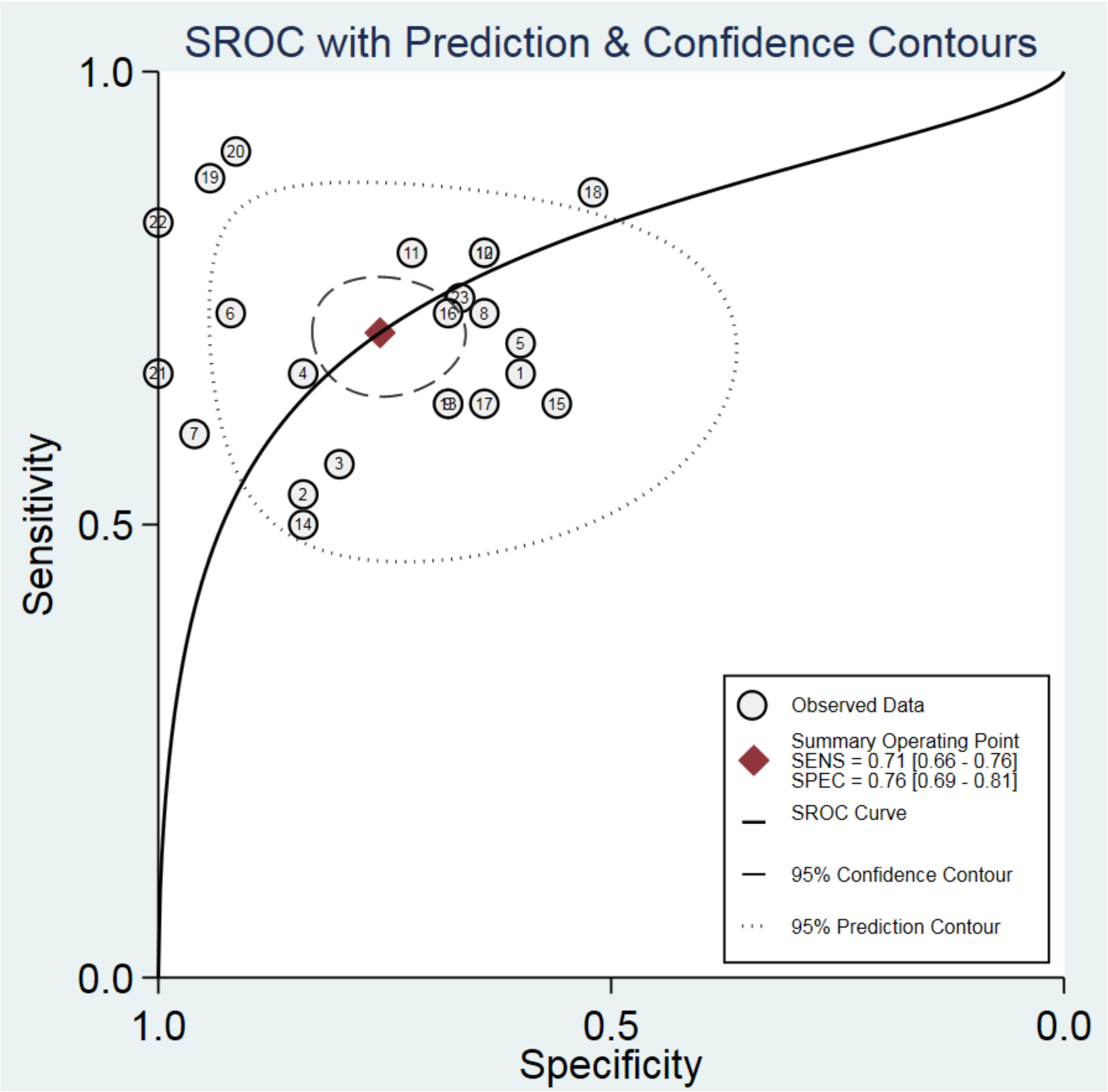

Fig S39: SROC curve of the meta-analysis of oral microbiome in the diagnosis of PC using genus-levels.

Bacterial taxonomy, Species level

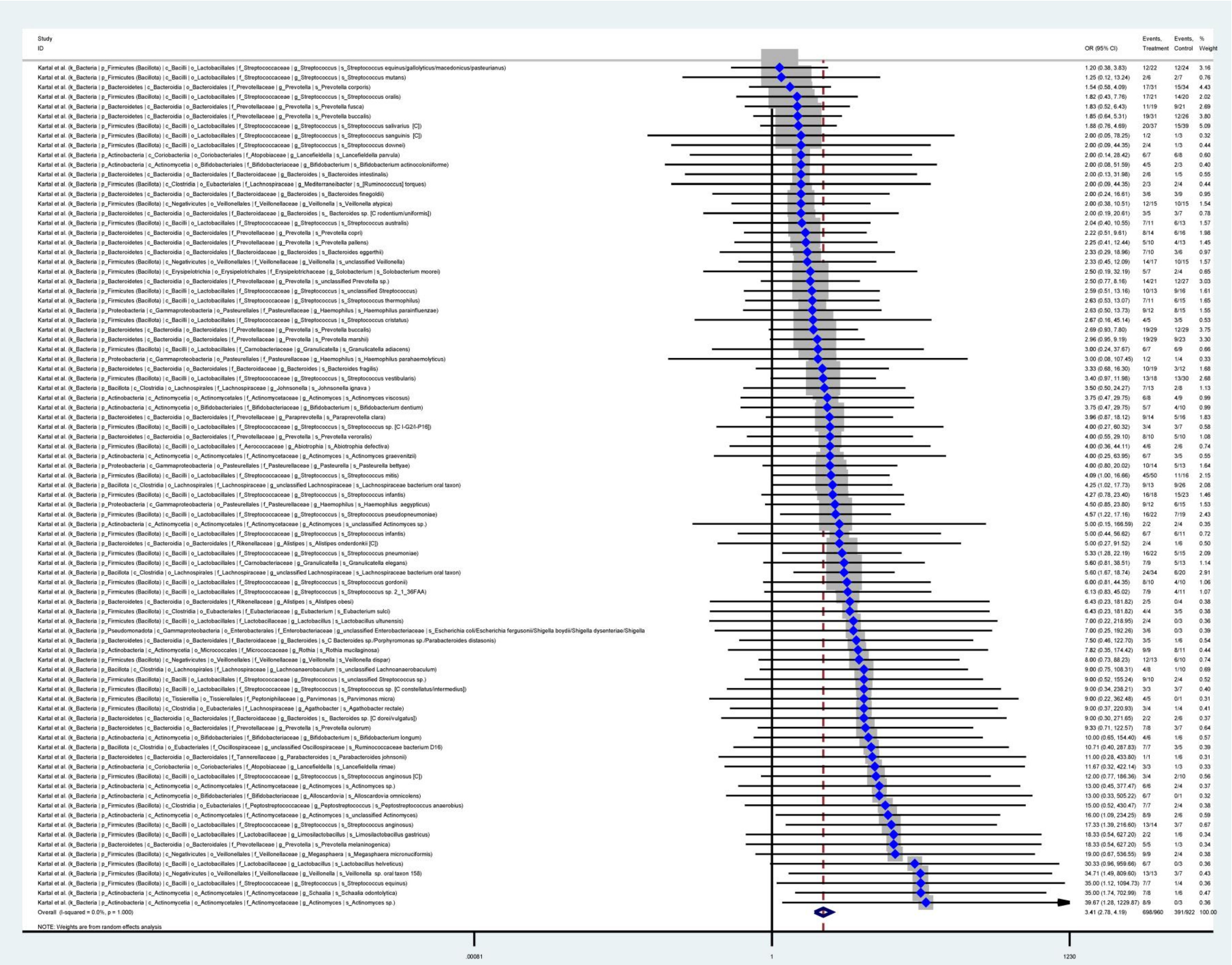

Fig S40: Forest plot of the meta-analysis of oral microbiome in the diagnosis of PC using species levels.

Bacterial taxonomy, Species level

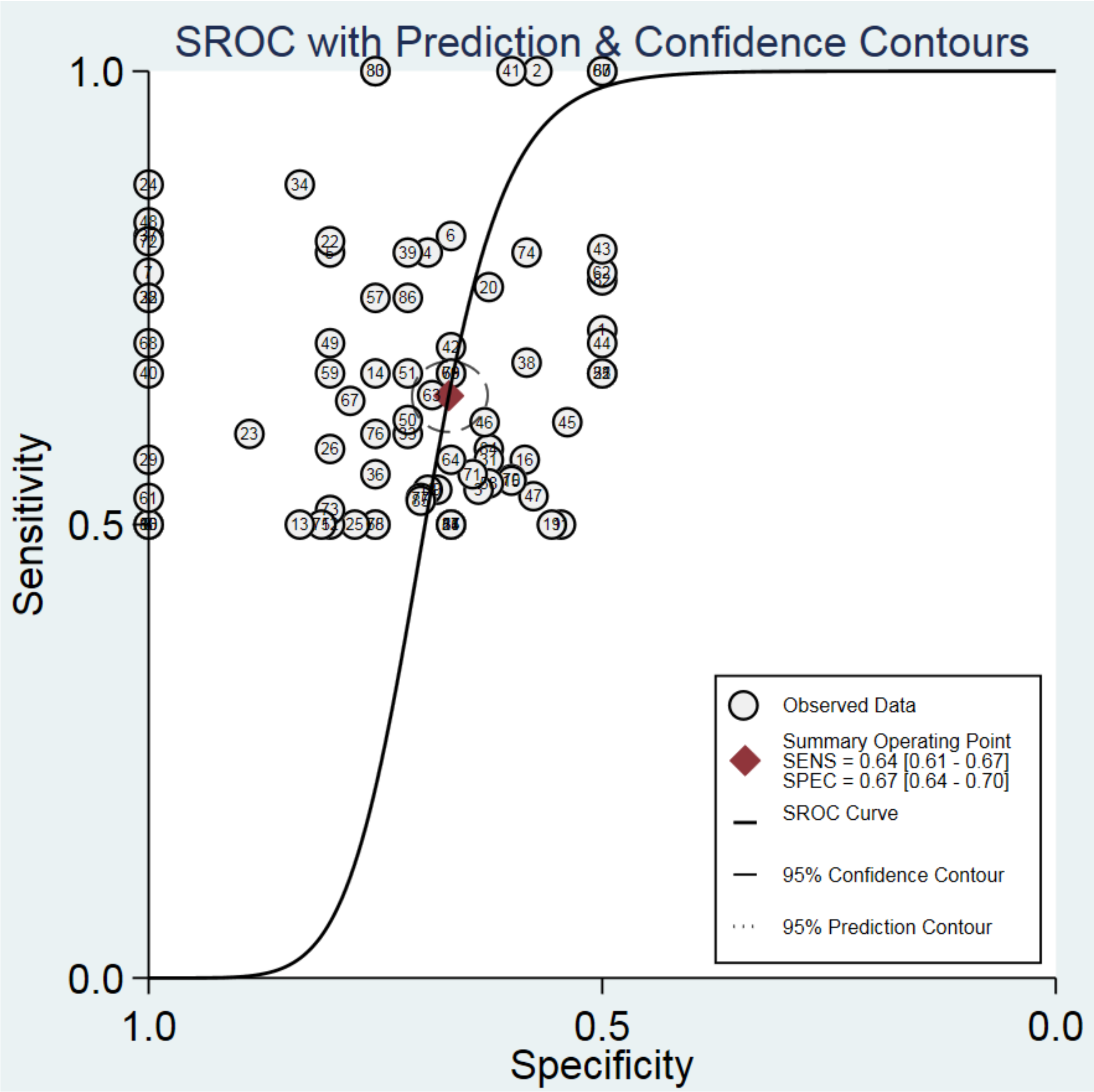

Fig S41: SROC curve of the meta-analysis of oral microbiome in the diagnosis of PC using species levels.

Subgenus-level taxonomy *(k_Bacteria | p_Firmicutes (Bacillota) | c_Bacilli | o_Lactobacillales | f_Streptococcaceae | g_Streptococcus)*

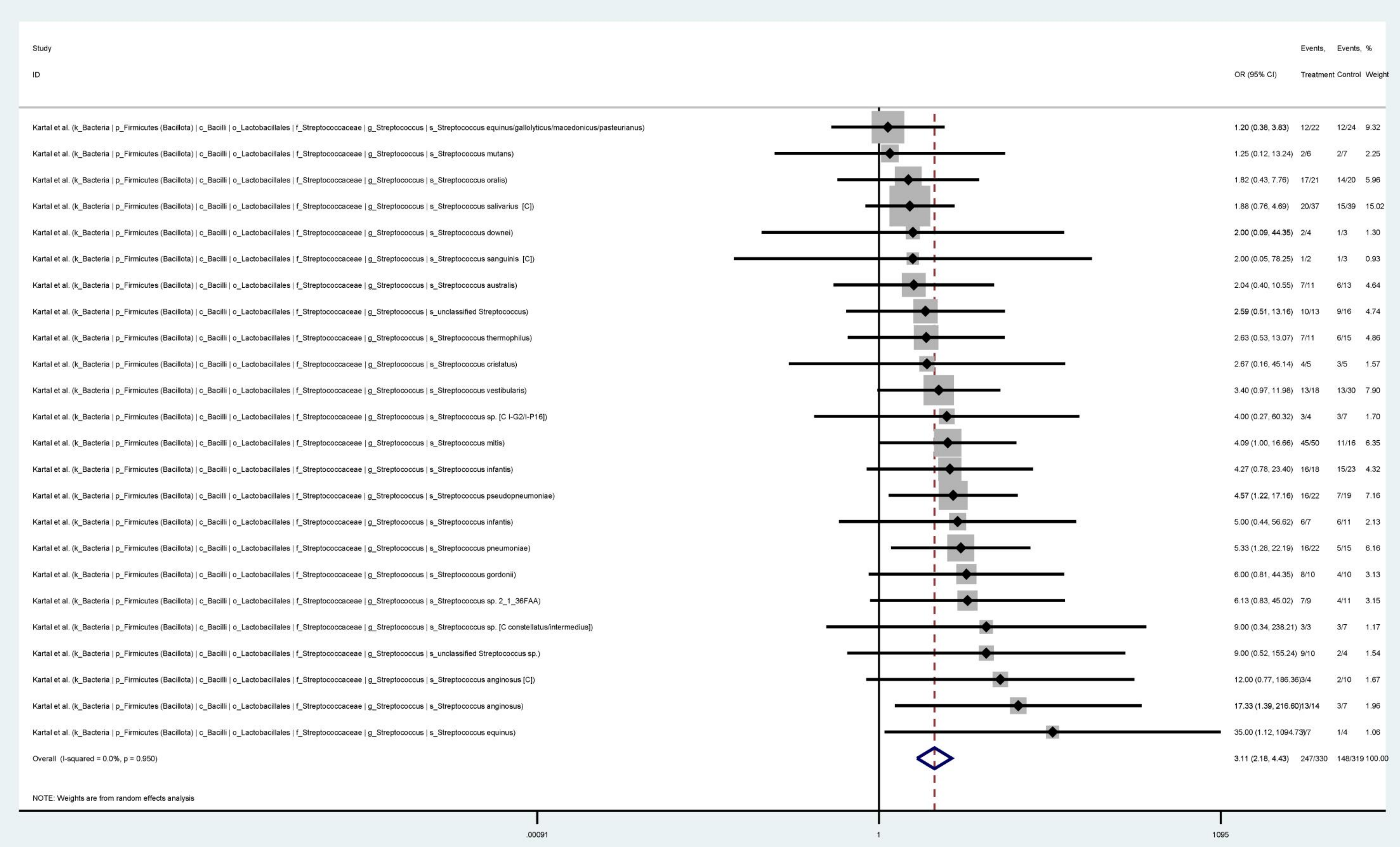

Fig S42: Forest plot of the meta-analysis of oral microbiome in the diagnosis of PC using subgenus-level taxonomy (*g_Streptococcus*).

Subgenus-level taxonomy ***(k_Bacteria | p_Firmicutes (Bacillota) | c_Bacilli | o_Lactobacillales | f_Streptococcaceae | g_Streptococcus)***

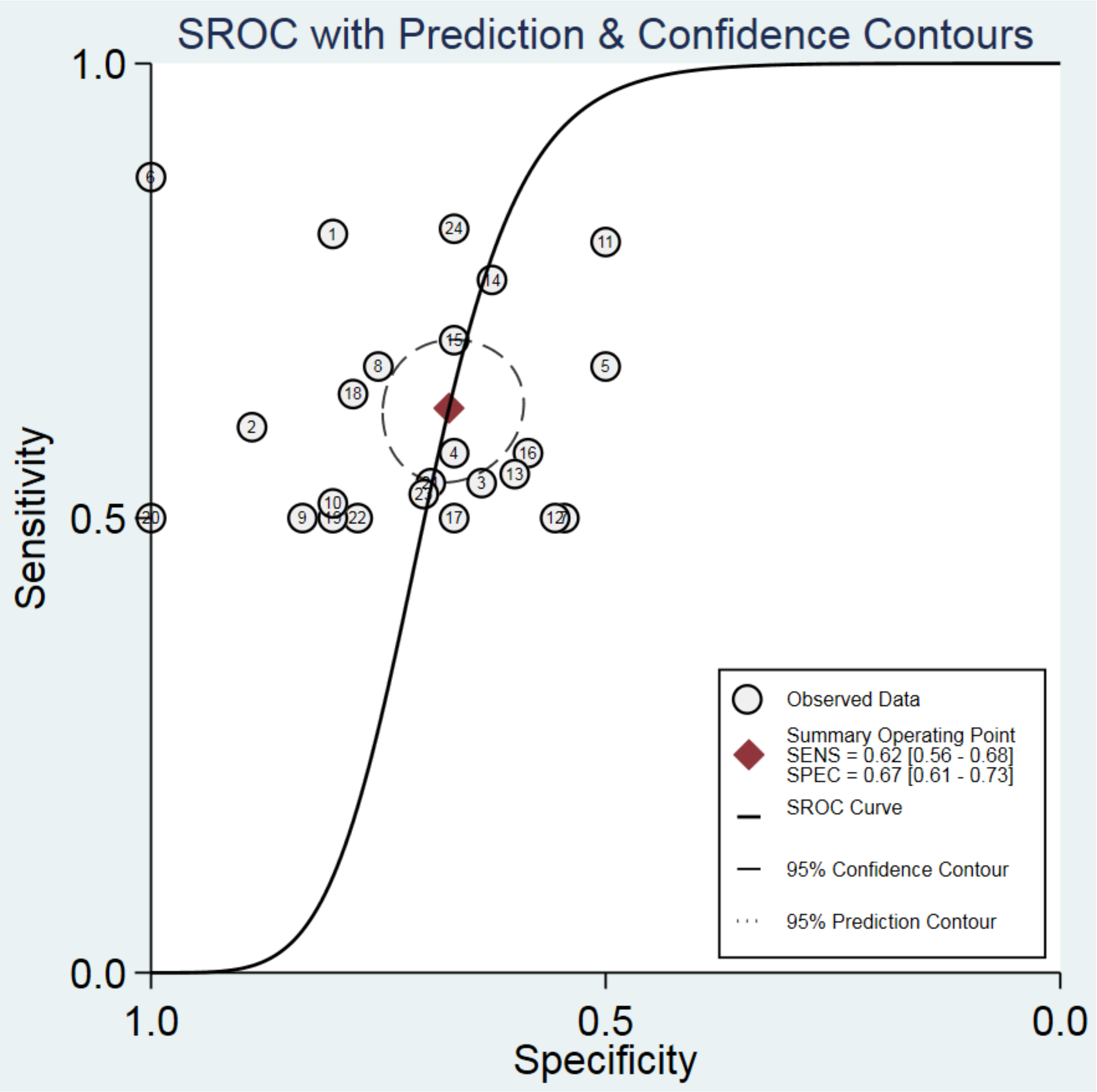

Fig S43: SROC curve of the meta-analysis of oral microbiome in the diagnosis of PC using subgenus-level taxonomy (*g_Streptococcus*).

Subgenus-level taxonomy *(k_Bacteria | p_Bacteroidetes | c_Bacteroidia | o_Bacteroidales | f_Prevotellaceae | g_Prevotella)*

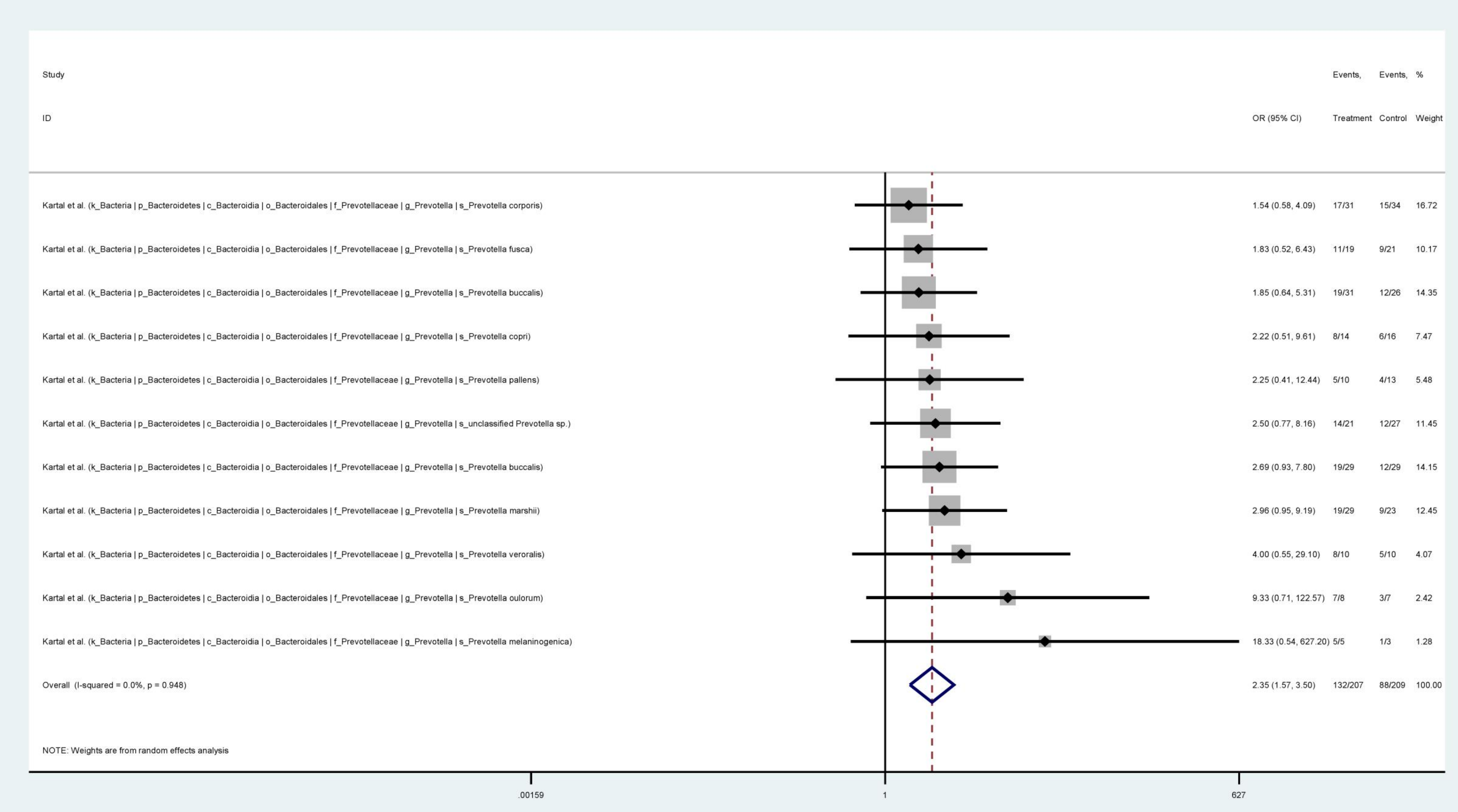

Fig S44: Forest plot of the meta-analysis of oral microbiome in the diagnosis of PC using subgenus-level taxonomy (*g_Prevotella*).

Subgenus-level taxonomy ***(k_Bacteria | p_Bacteroidetes | c_Bacteroidia | o_Bacteroidales | f_Prevotellaceae | g_Prevotella)***

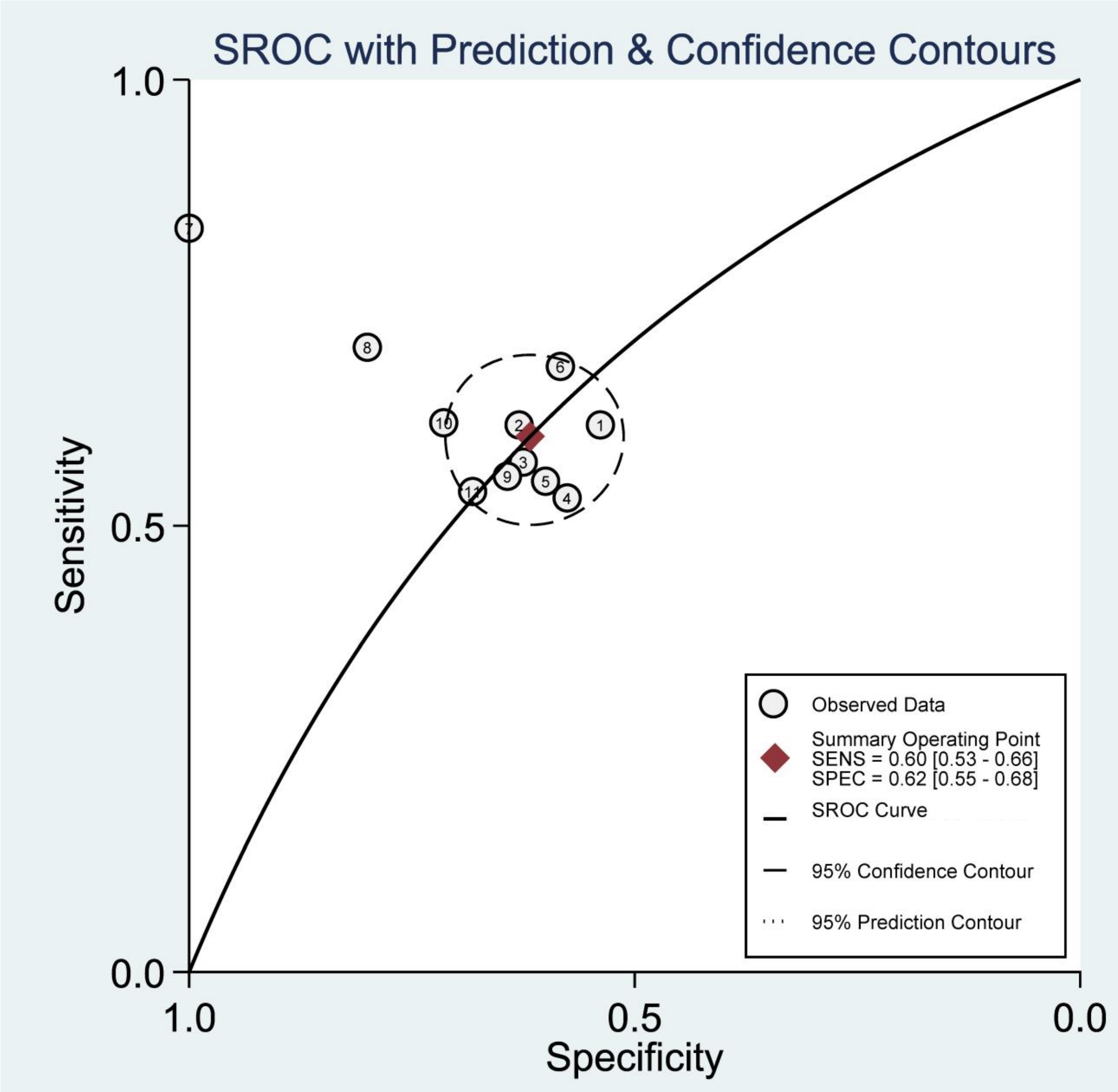

Fig S45: SROC curve of the meta-analysis of oral microbiome in the diagnosis of PC using subgenus-level taxonomy (*g_Prevotella*).

Pancreatic cancer (PC) versus chronic pancreatitis (CP)

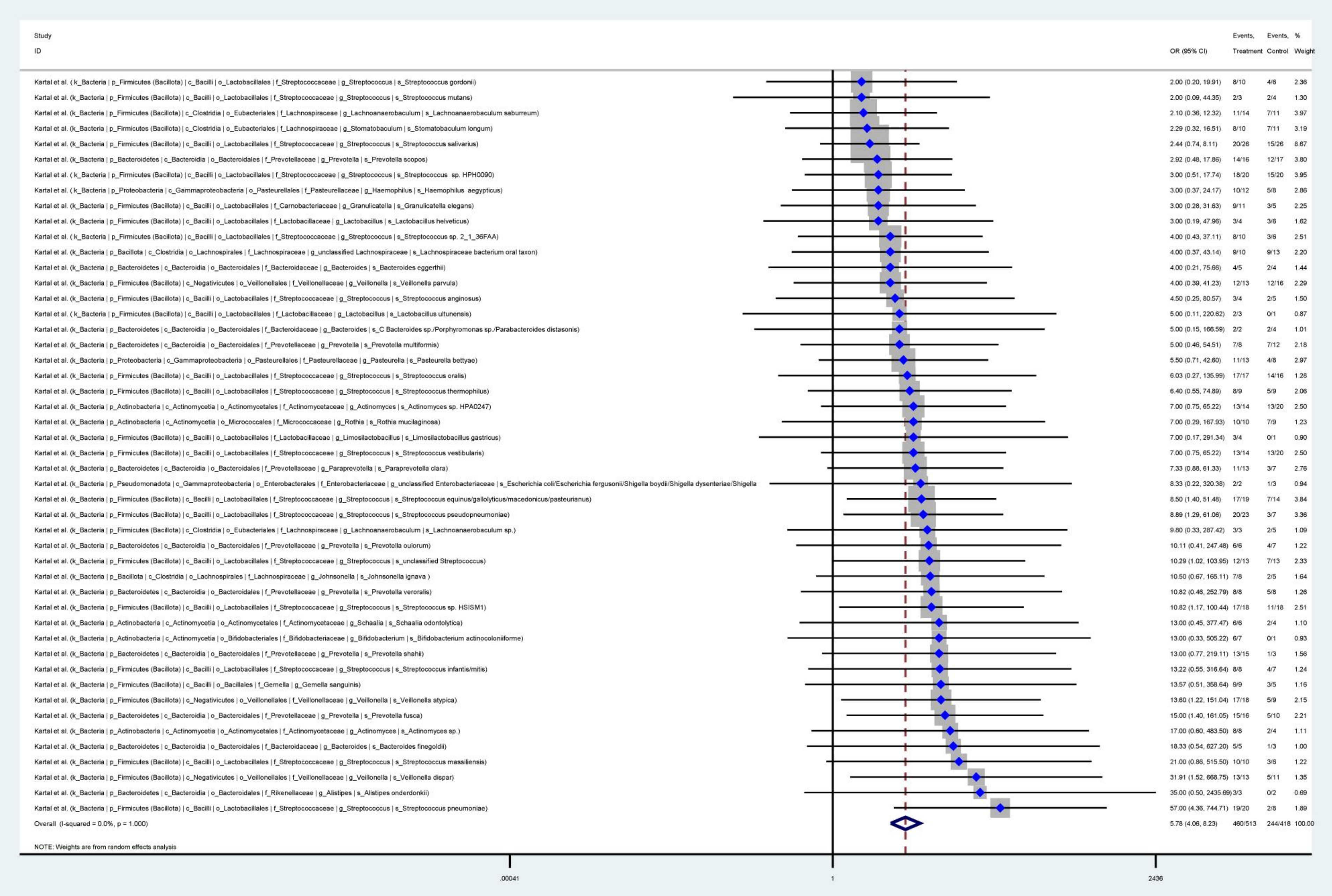

Fig S46: Forest plot of the meta-analysis of single oral microbiome in diagnosis of pancreatic cancer (PC) versus chronic pancreatitis (CP).

Pancreatic cancer (PC) versus chronic pancreatitis (CP)

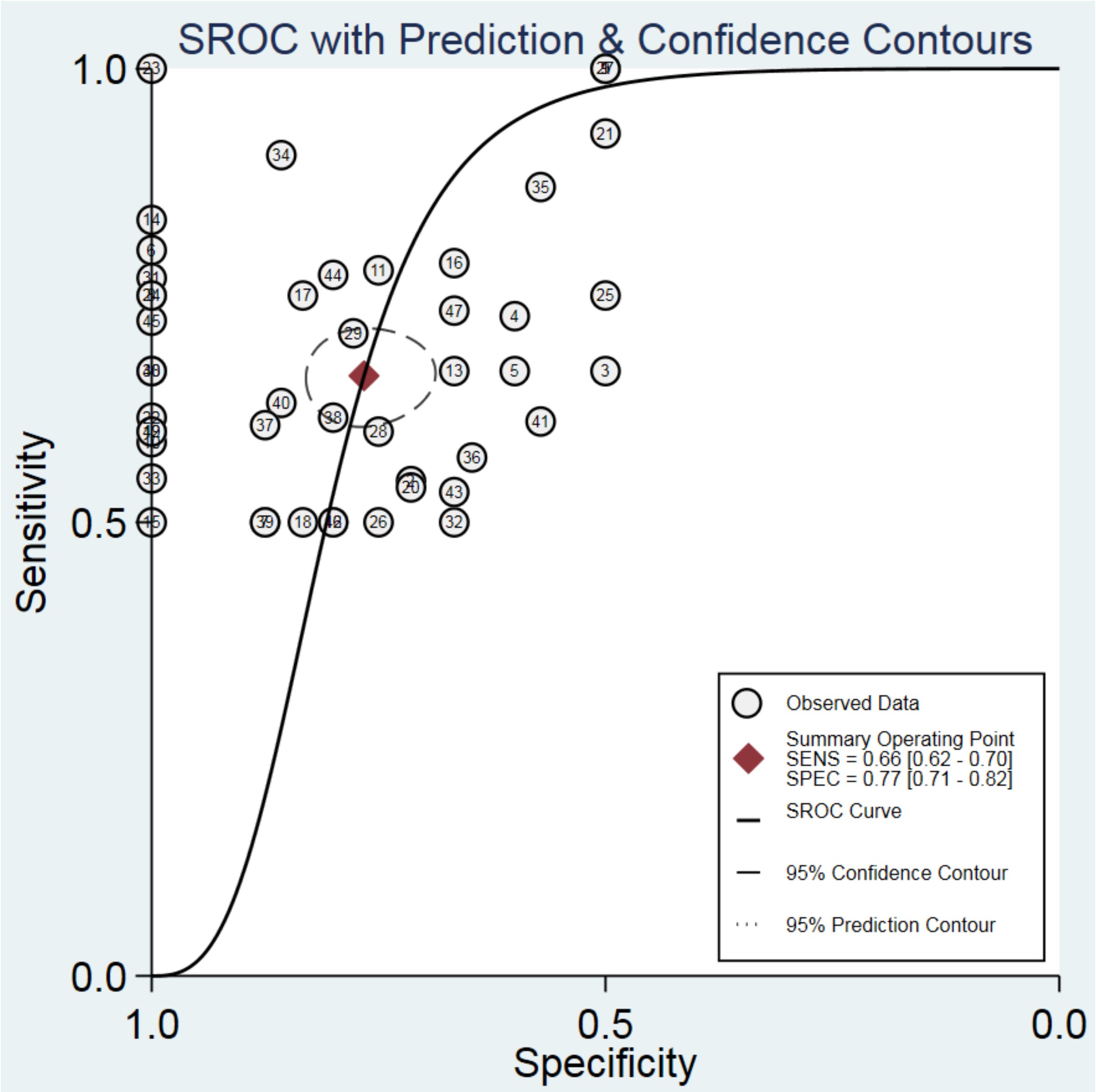

Fig S47: SROC curve of the meta-analysis of oral microbiome in the diagnosis of PC versus chronic pancreatitis (CP).

Subgenus-level taxonomy (k_Bacteria | p_Firmicutes (Bacillota) | c_Bacilli | o_Lactobacillales | f_Streptococcaceae | g_Streptococcus)

Pancreatic cancer (PC) versus chronic pancreatitis (CP)

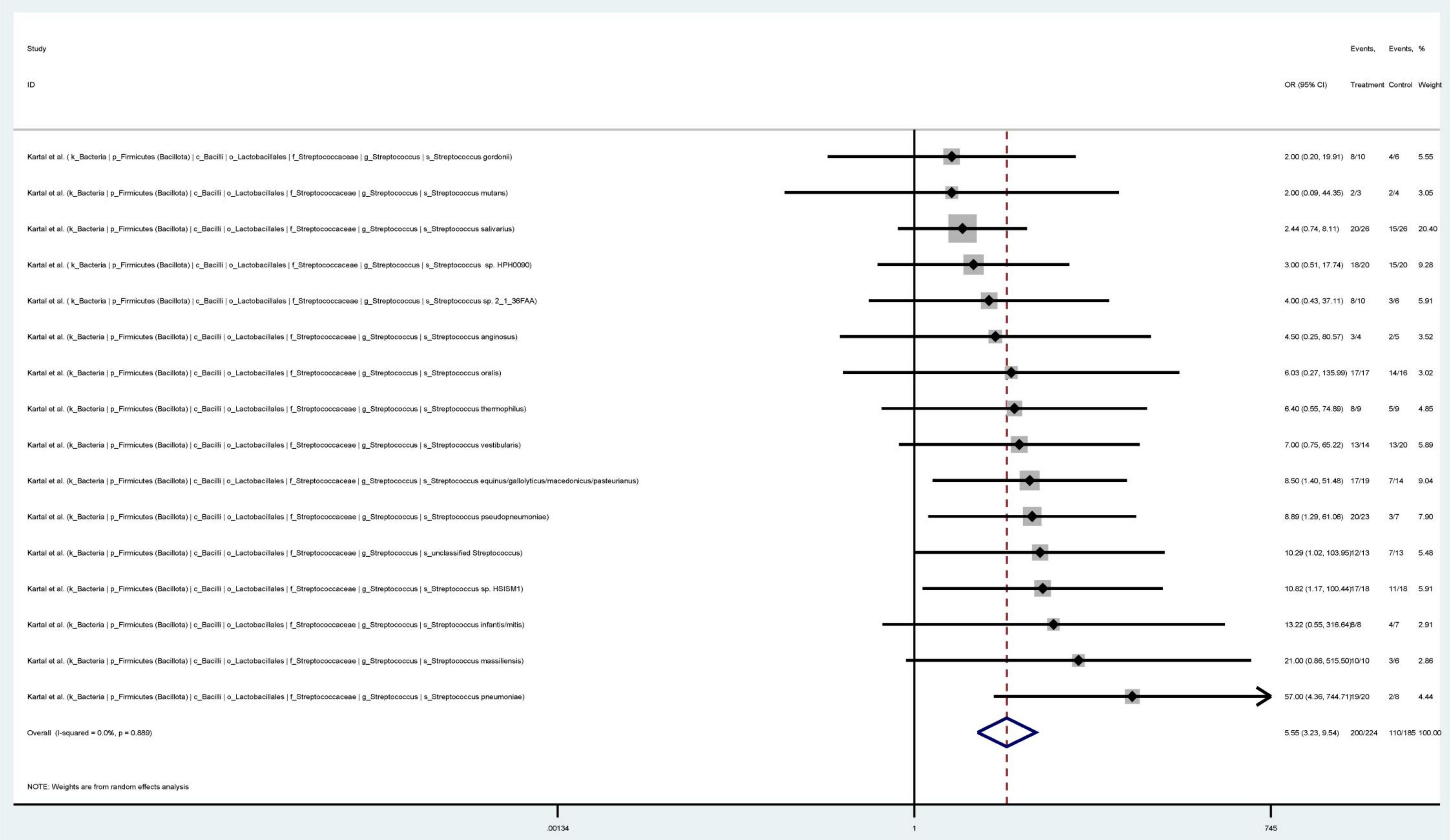

Fig S48: Forest plot of the meta-analysis of single oral microbiome (*g_Streptococcus*) in diagnosis of pancreatic cancer (PC) versus chronic pancreatitis (CP).

Subgenus-level taxonomy (k_Bacteria | p_Firmicutes (Bacillota) | c_Bacilli | o_Lactobacillales | f_Streptococcaceae | g_Streptococcus)

Pancreatic cancer (PC) versus chronic pancreatitis (CP)

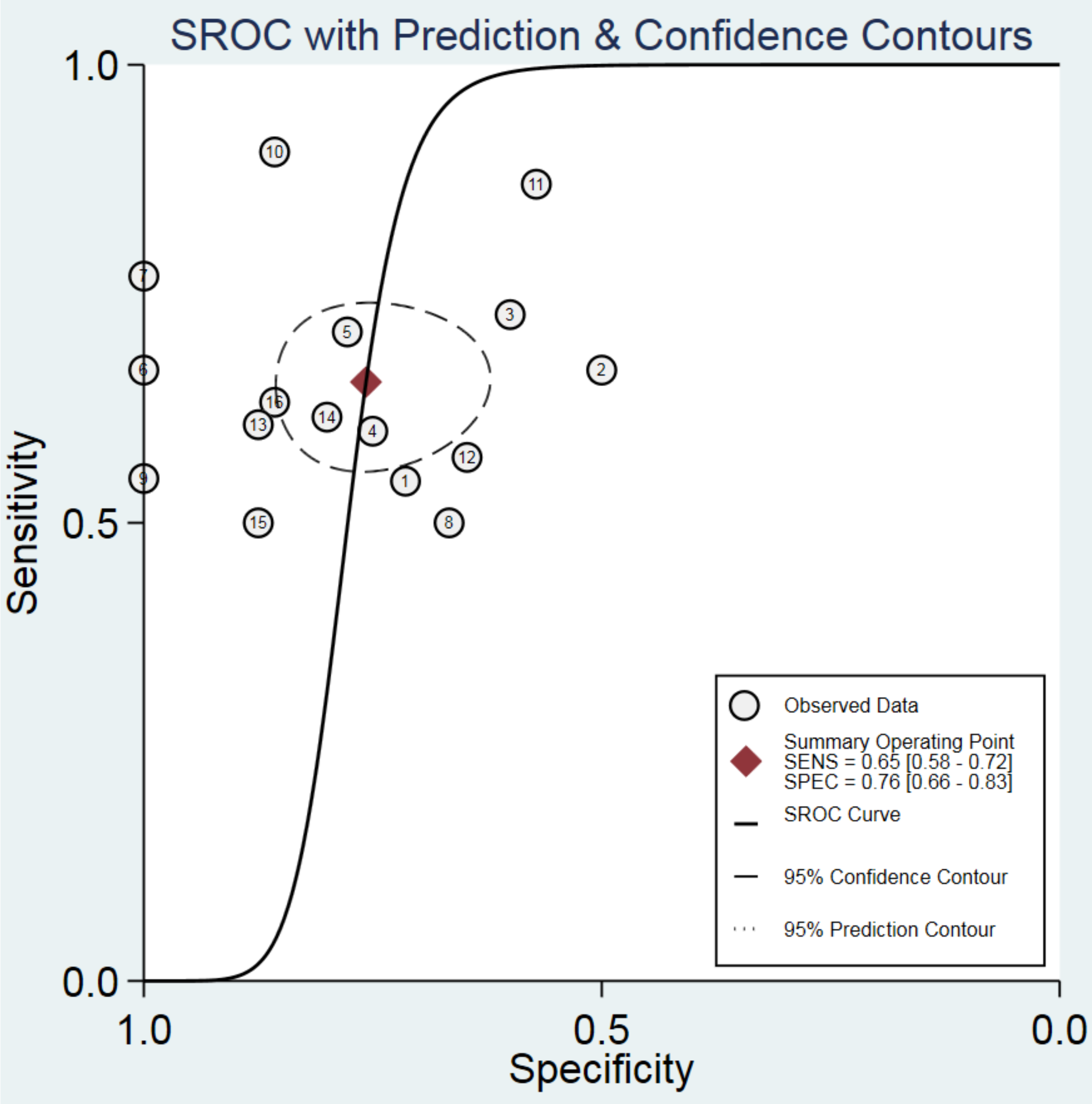

Fig S49: SROC curve of the meta-analysis of oral microbiome (*g_Streptococcus*) in the diagnosis of PC versus chronic pancreatitis (CP).

Subgenus-level taxonomy *(k_Bacteria | p_Bacteroidetes | c_Bacteroidia | o_Bacteroidales | f_Prevotellaceae | g_Prevotella)*
Pancreatic cancer (PC) versus chronic pancreatitis (CP)

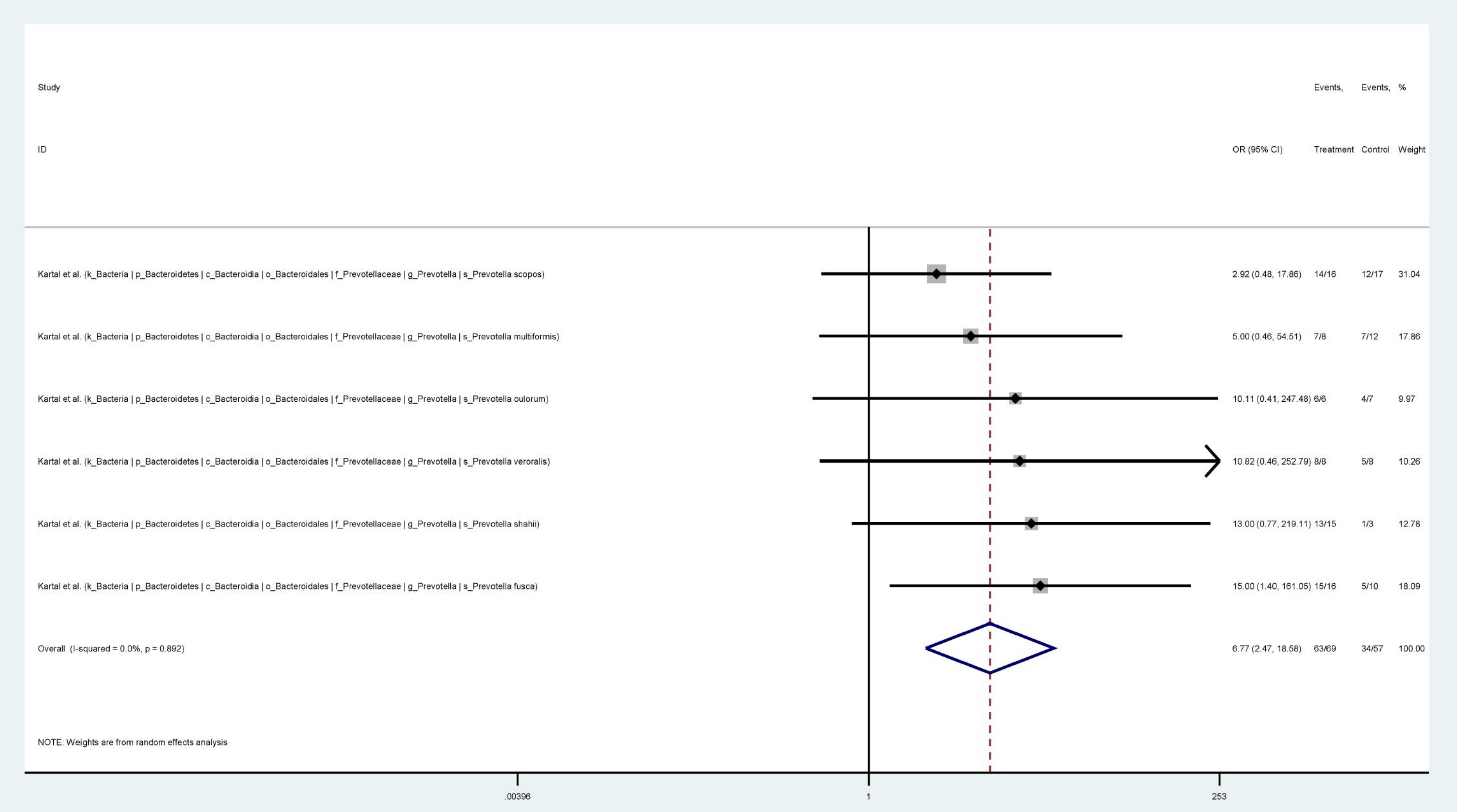

Fig S50: Forest plot of the meta-analysis of oral microbiome (*g_Prevotella*) in diagnosis of pancreatic cancer (PC) versus chronic pancreatitis (CP).

Subgenus-level taxonomy *(k_Bacteria | p_Bacteroidetes | c_Bacteroidia | o_Bacteroidales | f_Prevotellaceae | g_Prevotella)*
Pancreatic cancer (PC) versus chronic pancreatitis (CP)

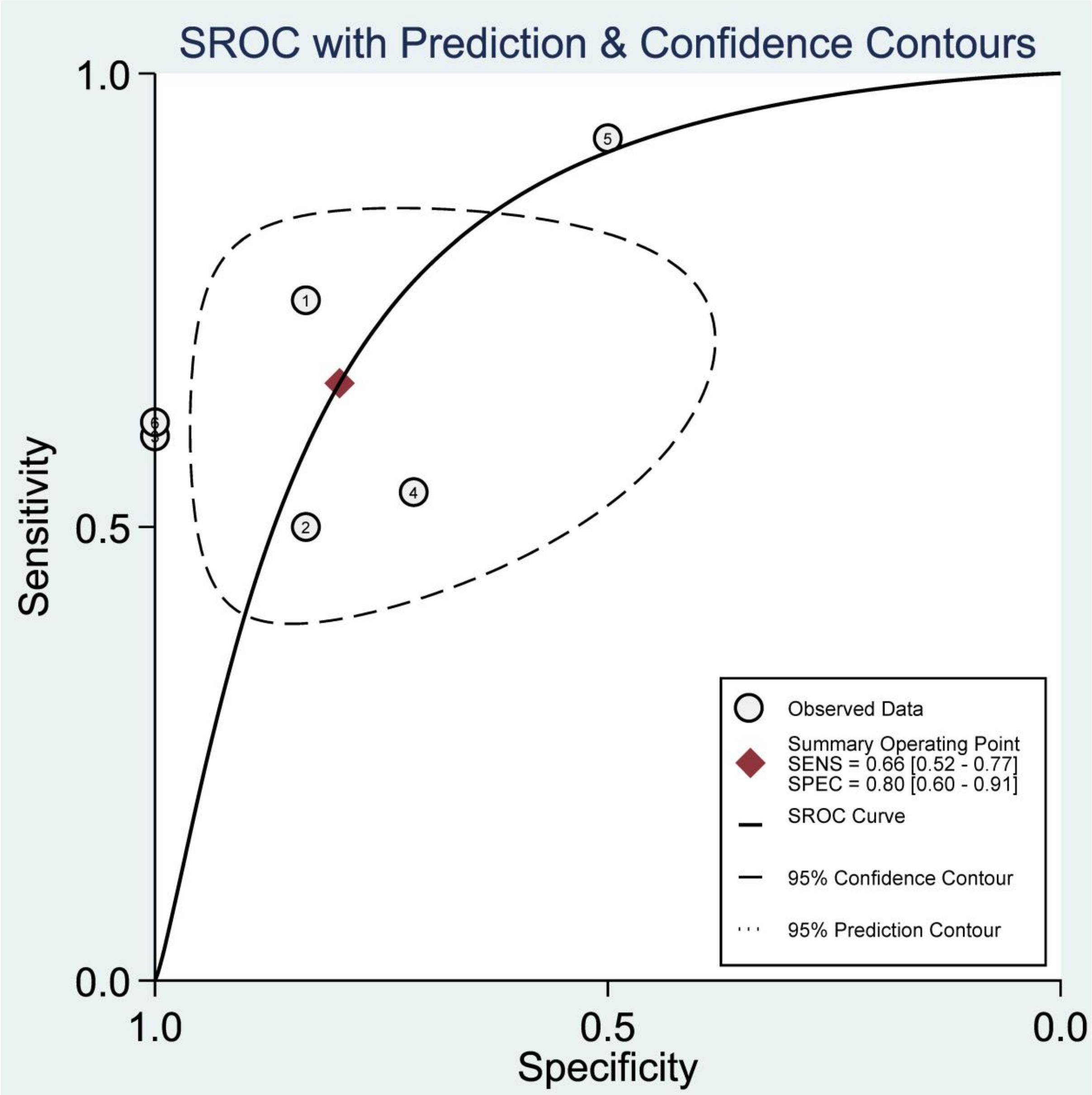

Fig S51: SROC curve of the meta-analysis of oral microbiome (*g_Prevotella*) in the diagnosis of PC versus chronic pancreatitis (CP).
